# An Ultramicroporous Physisorbent Sustained by a Trifecta of Directional Supramolecular Interactions

**DOI:** 10.1021/jacs.4c13797

**Published:** 2025-01-02

**Authors:** Alan C. Eaby, Shaza Darwish, Shi-Qiang Wang, Andrey A. Bezrukov, Debobroto Sensharma, Angela Shipman, Carlos J. Solanilla, Brian Space, Soumya Mukherjee, Michael J. Zaworotko

**Affiliations:** † Bernal Institute and Department of Chemical Sciences, University of Limerick, Limerick V94 T9PX, Ireland; ‡ Department of Chemistry, North Carolina State University, Raleigh, North Carolina 27607, United States

## Abstract

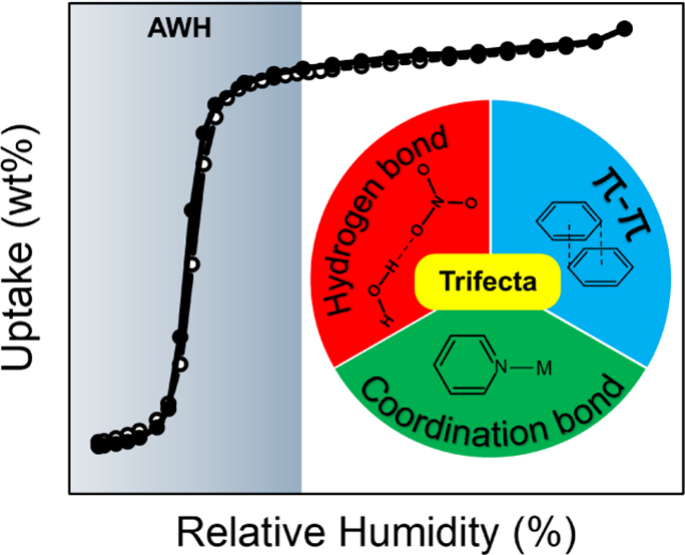

2D and 3D porous coordination networks (PCNs) as exemplified by metal–organic frameworks, MOFs, have garnered interest for their potential utility as sorbents for molecular separations and storage. The inherent modularity of PCNs has enabled the development of crystal engineering strategies for systematic fine-tuning of pore size and chemistry in families of related PCNs. The same cannot be said about one-dimensional (1D) coordination polymers, CPs, which are understudied with respect to porosity. Here, we report that permanent porosity is exhibited by the previously reported family of linear (L) 1D porous CPs, PCPs, of formula [M(bipy)(NO_3_)_2_(H_2_O)_2_]n (L-chn-1-M-NO3: M = Co, Ni; bipy = 4,4′-bipyridine). Their pore structure comprises 1D channels sustained by three types of directional interaction: coordination bonds; hydrogen bonds; offset π–π interactions. Heating L-chn-1-M-NO3 in vacuo or above 383 K resulted in removal of the aqua ligands and concomitant transformation to nonporous anhydrate phases ZZ-chn-1-Co-NO3 (ZZ = zigzag) and HT-Ni. Exposure of these anhydrate phases to ambient humidity resulted in regeneration of L-chn-1-M-NO3. That L-chn-1-M-NO3 exhibits permanent porosity was supported by CO_2_ and water sorption measurements, which afforded reversible type I and stepped (S-shaped) isotherm profiles, respectively, making this work the first demonstration of reversible water sorption in a 1D PCP. The water sorption properties are pertinent to atmospheric water harvesting: onset of uptake at ca. 12% relative humidity; activation required only mild heat or vacuum; relatively fast adsorption/desorption kinetics; performance retained over >100 adsorption/desorption cycles. We project water harvesting productivity of L-chn-1-M-NO3 of 3.3 L kg^–1^ d^–1^, on par with some leading MOF desiccants. DFT and Monte Carlo simulations provide insights into the structure of water molecules in the channels, provide their influence on the host framework, and provide a plausible argument for the experimental water vapor isotherms. This work demonstrates that easily scalable 1D PCPs, a potentially vast class of materials, can exhibit porous structures sustained by three types of directional supramolecular synthons and offer desirable water sorption properties.

## Introduction

Porous coordination polymers^1^ (PCPs) exemplified by metal–organic frameworks^2^ (MOFs), porous coordination networks (PCNs),^3^ or metal–organic materials^4^ (MOMs) are recognized as having the potential to solve global challenges such as removal of anthropogenic gases (CO_2_,^5^ CH_4_^6^) from the atmosphere, enabling efficient chemical commodity separations (C2,^7^ C3,^8^ and C8^9^ hydrocarbon separations^10^), and addressing water scarcity through atmospheric water harvesting (AWH).^11^ The three decades-long interest in PCPs with desirable sorption properties has provided insight into underlying structure–property relationships.^1,12^ Coordination network dimensionality has emerged as a parameter that can dictate the suitability of a PCP for sorption applications.^13^ High-dimensional (HD) 2D and 3D PCNs can be designed through self-assembly of linker ligands that connect nodes to afford predictable and robust network topologies.^14^ This makes them attractive targets for systematic crystal engineering^15^ studies that address small molecule capture, separation, and storage,^1,2,16^ as their sorption properties can be fine-tuned by modifying pore size and chemistry using the node-and-linker approach introduced by Hoskins and Robson.^14b^ Indeed, the design principles that afford permanent porosity in HD materials are now well-defined: the extent of porosity in 2D PCNs,^17^ such as those that possess a square-lattice topology (**sql**), exemplified by Hofmann clathrates,^18^ can be precisely tuned by modifying the flexibility and/or length of the linkers or the degree of network entanglement.^17^ Similarly, significant advancements in pore engineering have been achieved for 3D MOMs since the first extra-large-surface-area MOMs, HKUST-1^19^ and MOF-5,^20^ were reported in 1999. The most notable families are isoreticular MOFs,^21^ metal–azolate frameworks (MAFs),^22^ zeolitic–imidazolate frameworks (ZIFs),^23^ zeolite-like MOFs,^24^ and hybrid ultramicroporous materials (HUMs).^5^ The situation is not as well-developed for low dimensionality (LD) materials, as discovery of permanently porous molecular (0D) materials has been largely empirical, as exemplified by Dianin’s compound,^25^ tris(*o*-phenylenedioxy)phosphonitrile trimer,^26^ pillar[*n*]arenes,^27^ molecular porous materials,^28^ hydrogen-bonded organic frameworks (e.g., HOF-1),^29^ supramolecular organic frameworks (e.g., SOF-1),^30^ and molecular cages with 2D and 3D pores.^31^ Nevertheless, porosity can be engineered by targeting awkward molecular structures that pack inefficiently in the solid state to form extrinsic pores^32^ or through the aforementioned cages with intrinsic porosity. 1D PCPs whose structures also rely on weaker, noncovalent interactions are long recognized from a crystal engineering perspective.^33^ Such materials can form dense solids without accessible pores and remain understudied as porous sorbents,^34^ perhaps in part because those 1D CPs that exhibit solvent-containing interstitials often collapse upon guest release.^35^ 1D CPs have therefore largely been overlooked in gas sorption and storage, and an effective approach to design 1D PCPs *ab initio* has not yet been established.^36^

The potential for 1D CPs in sorption applications is not limited by synthetic accessibility, scalability, or lack of structural diversity. Indeed, 1D CPs are inherently modular and can exhibit structural diversity like their HD counterparts owing to the myriad of possible molecular building block (MBB) combinations that can arise from using different linker ligands, terminal ligands, metal ions, counterions, and solvents.^36^ This is evident from the number of 1D CPs that have been deposited in the Cambridge Structure Database^37^ (CSD, v.5.45; database updates: Mar and Jun 2024) of the 126,800 structures in the MOF subset,^38^ and 35,743 are 1D CPs. These structures form several prominent structural motifs which have the potential to form porous structures:^36^ linear chain (**chn**); zigzag (**ZZ**); ladder; ribbon; helix; rotaxanes. Despite the relatively large number of 1D CPs, only a handful have been shown to demonstrate permanent porosity (Table S1). Porosity in these structures is maintained by a combination of directional noncovalent interactions (hydrogen bonds, π–π, CH-π) between interwoven or slanted polymer strands that form extrinsic voids or connect intrinsic voids that are maintained during solvent release. It stands to reason that if we are to crystal engineer robust 1D PCPs, then strong and directional noncovalent interactions are necessary to construct platforms or families of isostructural materials. The hydrogen bond^39^ is perhaps the most studied owing to its directionality and relative strength^40^ and has been successfully used to construct porous molecular materials.^41^ However, only a few hydrogen-bonded PCPs (HBPCPs) have been studied (Table S1).

**Figure 1:**
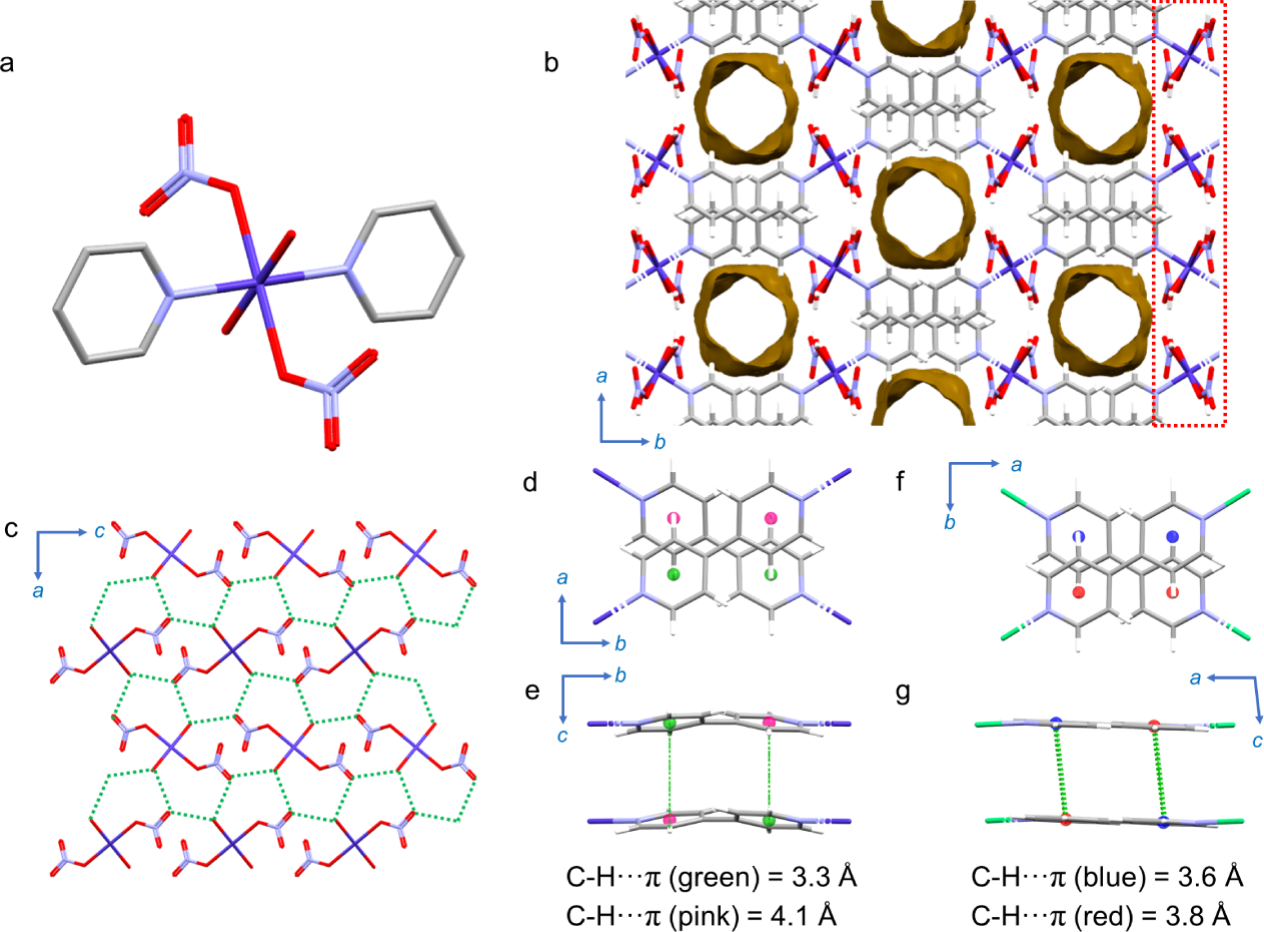
Capped-stick models of **L-chn-1-Co-NO3** showing the (a) MBB, its porous crystal structure (b), and noncovalent interactions (c–e). (a) Projection along [11] showing the mononuclear MBB. (b) Projection along [001] displaying the 1D channels with guest-accessible voids shown as yellow surfaces (Mercury,^42^ 1.2 Å probe-radius). Atoms that are involved in hydrogen-bond interactions in the plane (010) are shown with a red-dotted rectangle. (c) Projection along [010] showing close contacts of O–O (green-dotted line) that are involved in hydrogen-bond interactions. C–H···π interactions between bipy moieties shown (green-dotted line) along (d) [001] and (e) [100] for **L-chn-1-Co-NO3** and along (f) [001] and (g) [100] for **L-chn-1-Ni-NO3** with symmetry equivalent aryl ring centroids shown as green and pink (d, e) and blue and red (f, g) spheres. Atom colors: gray, carbon; white, hydrogen; blue, nitrogen; red, oxygen; purple, cobalt; light green, nickel. Some atoms have been omitted for the sake of clarity.

As a part of our ongoing studies on ultramicroporous solids,^42^ we studied and report herein the sorption properties of the 1D CP family: [M(bipy)(NO_3_)_2_(H_2_O)_*x*_]_n_ (M = Co, Ni; bipy = 4,4′-bipyridine; x = 0, 2). These compounds can be prepared from the reaction of hydrated metal nitrates and bipy in a 1:1 ratio, giving a mononuclear MBB (Figure 1a). The 1D chains pack in an ABAB stacking arrangement that are bridged by hydrogen-bond interactions between adject nitrate and aqua ligands forming ultramicroporous 1D channels. We recently revisited these compounds and demonstrated that they could be synthesized using the easily scalable methods of mechanochemistry and water slurry making them suitable for scale-up.^43^ Therein, the general classification of **chn-1-M-NO3** (**chn** = chain; **1** = bipy; M = Co, Ni, Cu, Zn) was introduced, and we now extend this to include the type of structural motif (**Y-chn-1-M-NO3**; Y = L, linear; ZZ, zigzag). Mauger-Sonnek et al.^44^ conducted the first structure–property analysis on [Co(bipy)(NO_3_)_2_(H_2_O)_2_]_n_ and [Ni(bipy)(NO_3_)_2_(H_2_O)_2_]_n_ and reported that heating led to the removal of the aqua ligands forming unknown microcrystalline solids with formulas [Co(bipy)(NO_3_)_2_]_n_ and [Ni(bipy)(NO_3_)_2_]_n_, respectively. N_2_ (77 K) sorption analyses indicated that the phases obtained by activation under a dynamic vacuum were nonporous. However, owing to the activation conditions used, these sorption analyses were conducted upon the anhydrous nonporous phases rather than the guest-free [Co(bipy)(NO_3_)_2_(H_2_O)_2_]_n_ and [Ni(bipy)(NO_3_)_2_(H_2_O)_2_]_n_ structures. Hence, the sorption properties of this family of compounds remain largely unexplored. Recently, Sakamato et al.^45^ demonstrated that a reversible guest-induced structural conversion can occur in **L-chn-1-Co-NO3**: **L-chn-1-Co-NO3** was transformed into a 2D tongue-and-groove bilayer structure after exposure to bipy and EtOH and regenerated after exposure to water vapor. Herein, we present an experimental and theoretical investigation of the H_2_O sorption properties of these 1D CPs and compare them to benchmark desiccants.

## Results and Discussion

While the crystal structures of **L-chn-1-Co-NO3** and **L-chn-1-Ni-NO3** were previously reported,^44^ the crystallographic locations of sorbed water molecules in the channels were not analyzed. Single-crystal X-ray diffraction (SCXRD) analysis of the as-synthesized crystals (see Supporting Information) confirmed that the crystal structures are consistent with the literature. The linear 1D chains in both **L-chn-1-Co-NO3** and **L-chn-1-Ni-NO3** stack in an ABAB packing arrangement slanted at ca. 60° with respect to one another. This inefficient packing results in extrinsic voids in the form of 1D channels (Figure 1b) that propagate along [001] and account for ca. 18.8% and 19.9% of the unit cell volumes in **L-chn-1-Co-NO3** and **L-chn-1-Ni-NO3**, respectively. The minor differences in their crystal structures can be attributed to the difference in orientation of the aqua and nitrate ligands along the 1D chain (Figure S1). The channels in both **L-chn-1-Co-NO3** and **L-chn-1-Ni-NO3** are enabled by two types of directional noncovalent interactions between the chains: aqua-ligand hydrogen atoms hydrogen-bond with the neighboring nitrate oxygen atoms along the *ac* plane (Figure 1c);^46^ π–π interactions are between adjacent aryl rings along [001] (Figures 1d–g). Analysis of the difference electron density maps indicates that lattice water molecules are located throughout the channel (Figure S2): ca. 2H_2_O molecules per asymmetric unit (ASU, equivalent to the formula units, i.e., [M(bipy)(NO_3_)_2_(H_2_O)_2_]_n_) in the channels of **L-chn-1-Co-NO3** (PLATON^47^ SQUEEZE;^48^ 77 e^–^ per unit cell, UC) and ca. 1.4 H_2_O/ASU (55 e^–^/UC) in those of **L-chn-1-Ni-NO3**, whereas H_2_O oxygen atoms were observed in the channel (Figure S3), and they were found to have a low site occupancy because of disorder over several crystallographic positions (see Supporting Information). The channels in **L-chn-1-Co-NO3** and **L-chn-1-Ni-NO3** are narrow enough to accommodate two water molecules side-by-side and thus likely form a hydrogen-bonded network of one-dimensional chains or tapes.^49^ However, because of the positional disorder, a reasonable model for the water network could not be obtained from either crystal structure.

**Figure 2:**
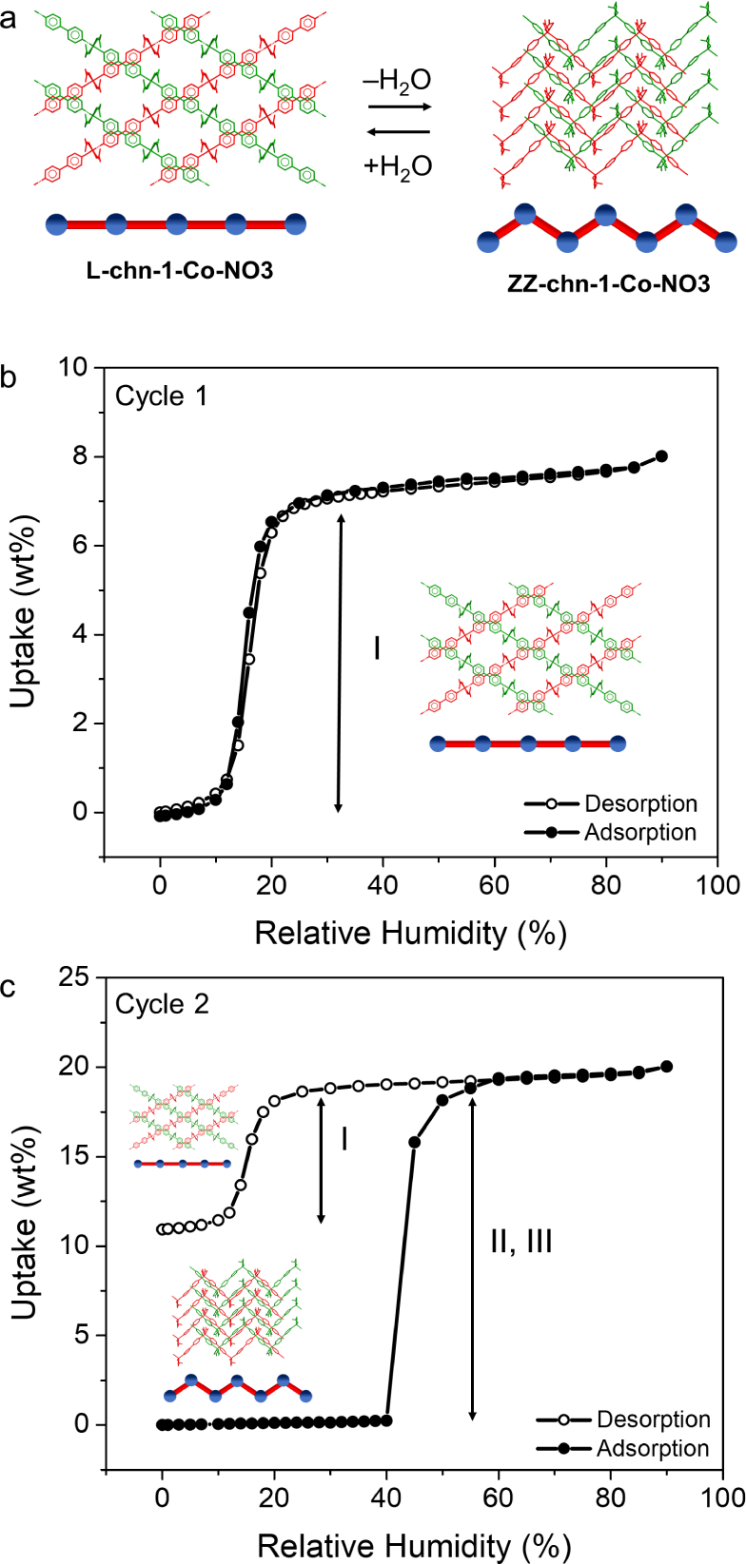
(a) Illustration of the structural transformation to **ZZ-chn-1-Co-NO3** that occurred during the removal of aqua ligands from **L-chn-1-Co-NO3**. Projections are shown along [001] and [010] for **L-chn-1-Co-NO3** and **ZZ-chn-1-Co-NO3**, respectively, perpendicular to their ABAB packing arrangements. Atoms in each layer are shown as green or red. Equilibrium water adsorption (closed circles) and desorption (open circles) isotherms of (b) **L-chn-1-Co-NO3** and (c) **ZZ-chn-1-Co-NO3** measured on a DVS instrument at 298 K. The features of the sorption profiles are indicated with Roman numerals: I, pore-filling and pore emptying; II, recoordination of aqua ligands; and III, pore-filling that occurs after recoordination of aqua ligands.

Bulk microcrystalline powder samples were prepared from slurrying in ethanol (see Supporting Information for details). Bulk-phase purity was established from powder X-ray diffraction (PXRD, Figure S4) patterns. Thermogravimetric analysis (TGA, Figure S5) of **L-chn-1-Co-NO3** shows two distinct weight-loss steps of 7.3 wt % (1.5 H_2_O/ASU, heating from RT) and 8.9 wt % (1.9 H_2_O/ASU onset temperature *T* = 371 K). Similarly, **L-chn-1-Ni-NO3** displays two weight-loss steps of 7.9 wt % (1.6 H_2_O/ASU, *T* = 298 K) and 9.0 wt % (1.9 H_2_O/ASU, *T* = 395 K). These steps correspond to the sequential removal of channel water molecules and aqua ligands, respectively. Variable-temperature PXRD (VT-PXRD) analysis (Figures S6 and S7) revealed that removal of the aqua ligands induced a structural transformation with a corresponding color change (Figure S8). Comparison of these PXRD patterns with those calculated from the bipy- and nitrate-containing 1D crystal structures in the CSD^37^ indicates that the high-temperature phase of **L-chn-1-Co-NO3** is isostructural to **ZZ**-**chn-1-Zn-NO3**.^43^ Conversely, the high-temperature **L-chn-1-Ni-NO3** phase (**HT-Ni**) does not match any structures archived in the CSD (v.5.45).^30^**ZZ-chn-1-Co-NO3** and **HT-Ni** are metastable at room temperature, however, as exposure to ambient water vapor (ca. 55% relative humidity, RH) resulted in recoordination of aqua ligands and regeneration of **L-chn-1-Co-NO3** (Figures 2a and S6) and **L-chn-1-Ni-NO3** (Figure S7). Several attempts to obtain a crystal structure for **ZZ-chn-1-Co-NO3** were made by heating single crystals of **L-chn-1-Co-NO3** in situ but resulted in a single-crystal-to-polycrystalline conversion, and a structural model could not be obtained (Figure S10). To evaluate the porosity of each phase, CO_2_ sorption analysis was conducted from 0 to 1 bar at 195 and 298 K (Figures S11 and S12). **L-chn-1-Co-NO3** and **L-chn-1-Ni-NO3** maintained crystallinity during removal of channel water molecules when activated under dynamic vacuum (10^–6^ mmHg) at 298 K^50^ and displayed moderate CO_2_ uptake at 1 bar. Conversely, **ZZ-chn-1-Co-NO3** and **HT-Ni** (383 K, 10^–6^ mmHg) displayed negligible CO_2_ uptake. **L-chn-1-Ni-NO3** registered a higher relative uptake (1.55 mmol g^–1^, 195 K; 1.12 mmol g^–1^, 298 K) than **L-chn-1-Co-NO3** (0.58 mmol g^–1^, 195 K; 0.36 mmol g^–1^, 298 K) despite their similar channel dimensions. It should be noted that activation of a sorbent is usually conducted at temperatures higher than the onset temperature of desorption (determined by TGA) and often under reduced pressure.^51^ Had such activation been conducted, the guest-free porous phases would likely have been overlooked. Of the few CO_2_ sorption studies reported on 1D PCPs, the majority were conducted in the temperature range of 273–298 K (Table S1), yielding uptakes of 0.5–2.2 mmol g^–1^ CO_2_; only [Ni(C_12_H_26_N_6_)][Ni(CN)_4_]^52^ was studied at 195 K, affording a CO_2_ capacity of 2.2 mmol g^–1^ at 1 bar. The highest CO_2_ uptake was demonstrated by Cd(C_38_H_32_N_2_)_2_(ClO_4_)_2_,^53^ which has the largest pore dimensions of these 1D PCPs, 3.1 mmol g^–1^ at 1 bar (273 K).

The effect of humidity on **L-chn-1-Co-NO3** and **L-chn-1-Ni-NO3** motivated us to evaluate their water sorption properties. Since the water sorption properties of 1D HBPCPs have not been previously reported, there was no expectation of properties suitable for AWH or desiccation. For AWH, regeneration-optimized sorbents (ROSs)^54^ should possess an S-shaped isotherm with an inflection <30% RH for utility in arid regions, hydrolytic stability, low regeneration temperatures (<333 K), little hysteresis, and rapid sorption kinetics.^55^ Desiccation does not require low regeneration temperatures and can have type I^56^ sorption isotherms that would be undesirable for AWH. Establishing the structure–property relationships that govern the stability of hydrates also impacts the pharmaceutical industry, where spontaneous hydration is typically undesirable but sometimes unavoidable.^57^ The water vapor sorption isotherms of **L-chn-1-Co-NO3**, **L-chn-1-Ni-NO3**, **ZZ-chn-1-Co-NO3**, and **HT-Ni** were collected by dynamic vapor sorption (DVS). Three consecutive adsorption/desorption cycles (cycles 1–3), measured between 0 and 90% RH at 298 K, were conducted with differing activation (act) temperatures to determine the water vapor sorption profiles of the porous phases (cycle 1: act 298 K), nonporous phases (cycle 2: act 423 K), and the porous phases after regeneration (cycle 3: act 298 K) (see Supporting Information for details). Both **L-chn-1-Co-NO3** and **L-chn-1-Ni-NO3** exhibited S-shaped isotherms during cycle 1 (Figures 2b and S13a) with a sharp step starting at ca. 12% RH. These isotherms revealed uptakes of 7.6 wt % (106 g cm^–3^, 1.6 H_2_O/ASU) and 7.2 wt % (107 g cm^–3^, 1.5 H_2_O/ASU), respectively. The S-shaped isotherm profiles are consistent with either a pore-filling^58^ (type V^56^) or a structural transformation (phase switching^59^) mechanism. Since **L-chn-1-Co-NO3** and **L-chn-1-Ni-NO3** maintained their structures during and after the removal of channel water, as evidenced by VT-PXRD and TGA analysis (Figures S5–S7), we can assert that sorption had occurred by a pore-filling mechanism (I). The enthalpy of adsorption at first loading Δ*h*_ads,zero_ was determined to be −45.0 ± 0.1 kJ mol^–1^ and −45.4 ± 0.1 kJ mol^–1^ for **L-chn-1-Co-NO3** and **L-chn-1-Ni-NO3**, respectively (Figure S20). These values compare well with other MOFs that have Δ*h*_ads_ values with magnitudes that range from 36 to 75 kJ mol^–1^.^11a^ The desorption profile of **L-chn-1-Co-NO3** exhibited negligible hysteresis, whereas **L-chn-1-Ni-NO3** displayed modest hysteresis. For cycle 2, following activation at 423 K, **ZZ-chn-1-Co-NO3** displayed an adsorption isotherm (Figure 2c) with negligible uptake until 40% RH where an abrupt step was observed reaching a maximum loading of 19.6 wt %. The latter was found to correspond to 3.7 molecules of H_2_O/ASU.^60^ The desorption profile exhibits a large hysteresis loop with a step in the range of 20 to 10% RH reaching a minimum loading of 10.9 wt % (2.1 H_2_O/ASU). **HT-Ni** showed a gradual uptake until 45% RH, reaching 10.3 wt % (1.9 H_2_O/ASU), plateaued until 55% RH where a step occurred reaching a maximum loading of 19.6 wt %, corresponding to 3.7 H_2_O/ASU (Figure S13b).^60^ The desorption trace also displayed a large hysteresis loop reaching a minimum loading of 9.6 wt % (1.8 H_2_O/ASU). The adsorption isotherm of cycle 3 (Figure S14) traced the desorption isotherm of cycle 2 achieving the same relative uptakes (maximum and minimum for adsorption and desorption, respectively) for both **L-chn-1-Co-NO3** and **L-chn-1-Ni-NO3**. The relatively large uptake during cycle 2 is consistent with simultaneous recoordination of the aqua ligands (II) to form the porous phase followed by pore-filling (III) into the channels. Notably, this process was found to be reversible (Figure S15). The enthalpies associated with the pore emptying mechanism Δ*h*_des,I_ (I, Figures 2 and S13) and loss of aqua ligands and concomitant structural rearrangements Δ*h*_des,II_ (II, Figures 2 and S13) were determined using temperature-ramp differential scanning calorimetry (DSC) analysis (Figure S21). The values of Δ*h*_des,I_ = −51.8 and −48.0 kJ mol^–1^, for **L-chn-1-Co-NO3** and **L-chn-1-Ni-NO3**, respectively, are in agreement with the Δ*h*_ads_ values associated with pore-filling determined from DVS analysis (Figure S20): Δ*h*_ads_ values at the half uptake are −49.6 ± 0.2 kJ mol^–1^ (3.8 wt %) and −49.6 ± 0.4 kJ mol^–1^ (3.6 wt %) for **L-chn-1-Co-NO3** and **L-chn-1-Ni-NO3**, respectively. The values of Δ*h*_des,II_ = −50.8 kJ mol^–1^ and −51.4 kJ mol^–1^ for transformations **L-chn-1-Co-NO3** → **ZZ-chn-1-Co-NO3** and **L-chn-1-Ni-NO3** → **HT-Ni**, respectively.

Water adsorption onto unsaturated metal centers in rigid materials tends to occur at lower RH than pore-filling owing to the stronger binding affinity of water. However, **ZZ-chn-1-Co-NO3** and **HT-Ni** are nonporous, and water sorption in these phases involves recoordination of the aqua ligands and a large structural rearrangement (II) to form the porous phases **L-chn-1-Co-NO3** and **L-chn-1-Ni-NO3**, respectively. Regenerating the porous phases requires higher partial pressures (i.e., RH) than the pressures required to cause pore-filling.

**Figure 3:**
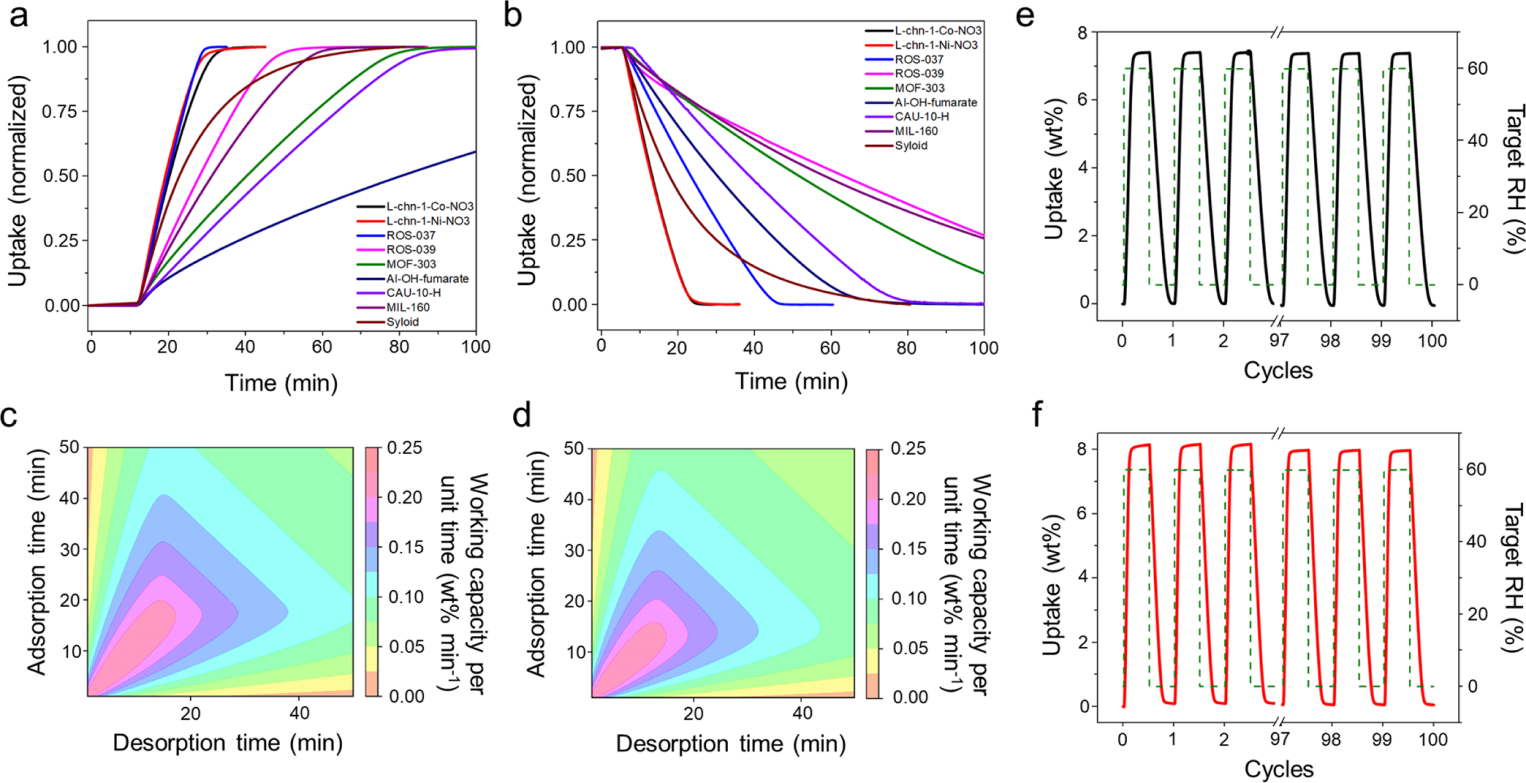
Water vapor (a) adsorption (0–30% RH) and (b) desorption (30–0% RH) kinetic plots measured for **L-chn-1-Co-NO3** (black), **L-chn-1-Ni-NO3** (red), and benchmark sorbents^54^ with a particle size of 50–100 μm using a DVS Adventure instrument at RT. Water vapor sorption productivity “heatmaps” calculated from the equilibrium adsorption isotherms of (c) **L-chn-1-Co-NO3** and (d) **L-chn-1-Ni-NO3** measured at RT. Water vapor sorption cycling experiments carried out on (e) **L-chn-1-Co-NO3** and (f) **L-chn-1-Ni-NO3** using a DVS Intrinsic instrument at RT: 100 consecutive 0–60% RH swing experiments for **L-chn-1-Co-NO3** (target RH of 20 min ads, 20 min des) and **L-chn-1-Ni-NO3** (target RH of 30 min ads, 30 min des).

While the sorption capacities of **ZZ-chn-1-Co-NO3** and **HT-Ni** are almost double those of **L-chn-1-Co-NO3** and **L-chn-1-Ni-NO3**, the high activation temperatures required to regenerate these phases after each water vapor sorption cycle (which involves the concomitant removal of the aqua ligands and structural transformations) make them unsuitable for AWH. Conversely, the S-shaped isotherms exhibited by **L-chn-1-Co-NO3** and **L-chn-1-Ni-NO3** are desirable for AWH which prompted a more in-depth analysis. We evaluated the following: 1) rates of water adsorption and desorption; 2) water harvesting productivity; and 3) hydrolytic stability. Powdered samples (10.9–11.1 mg dry mass, sieved 50–100 μm fraction) were tested in six consecutive RH-swing DVS experiments at 298 K (Figures S25 and S26): RH-swing cycles (cycles) 1–3 were conducted at 0–30% RH, followed by cycles 4–6 at 0–60% RH. Both adsorption and desorption kinetics curves follow the “pseudo-zero-order” kinetics typical for microporous materials with S-shaped isotherms.^54,61^ Our group has recently developed a model explaining this phenomenon and demonstrated that, under certain experimental conditions, sorption kinetics is a function of isotherm shape.^54^ Using this isotherm-based model, we were able to fit the sorption kinetics in **L-chn-1-Co-NO3** and **L-chn-1-Ni-NO3**. The kinetic coefficient *k* was consistent for each cycle, indicating good reproducibility over several cycles (*k* = 0.03, for 0–30% RH, Figures S28 and S29). To benchmark their water vapor sorption performances, **L-chn-1-Co-NO3** and **L-chn-1-Ni-NO3** were compared with benchmark sorbents (Figures 3a,b, S33 and S34, Table S2), whose kinetics were evaluated by Zaworotko and coworkers^54^ and an ROS protype, [Cu_2_(glutarate)_2_(bipy)]^62^ (**ROS-037**), which has similar channel dimensions and DVS isotherm inflection points (Figures S23 and S24): [Zn(1,2,4-triazole)F]_n_ (**ROS-039**), [Al(OH)(1*H*-pyrazole-3,5-dicarboxylate)]_n_ (**MOF-303**), [Al(OH)(fumarate)]_n_ (**Al–OH-fumarate**), [Al(OH)(benzene-1,3-dicarboxylate)]_n_ (**CAU-10-H**), [Al(OH)(2,5-furandicarboxylate)]_n_ (**MIL-160**), and **Syloid AL-1**, an industrial desiccant. Kinetics experiments for a comparable sample mass (Figure 3a,b, sample mass 10.9 mg) show that **L-chn-1-Co-NO3** and **L-chn-1-Ni-NO3** displayed the second and third fastest loading times (Figure 3a), with **ROS-037** being the fastest, and fastest unloading times when compared with these benchmark sorbents (Figure 3b). While full loading adsorption and desorption cycling provides the largest working capacity, water harvesting productivity can be optimized by using nonequilibrium cycling.^54^ The adsorption time (*t*_ads_) and desorption time (*t*_des_) required to achieve optimal water harvesting productivity (working capacity per unit time) under relevant conditions for AWH ([30% RH, 300 K] – [0% RH, 300 K]) were calculated for **L-chn-1-Co-NO3** and **L-chn-1-Ni-NO3** and plotted as “heatmaps” that identify adsorption–desorption times for optimal productivity (Figures 3c,d and S32) (see Supporting Information for calculation details). **L-chn-1-Co-NO3** and **L-chn-1-Ni-NO3** had identical maximum sorption productivity values of 0.23 wt %/min (*t*_ads_ = 1.0 min, *t*_des_ = 1.0 min), thereby yielding a projected optimal performance of 3.3 L kg^–1^ d^–1^. These water harvesting productivities are comparable with benchmark MOFs for AWH, **MOF-303**, **MOF-801**, **ROS-037,** and **ROS-039** that have reported projected performances of 1.3, 3.5, 6.6, and 7.3 L kg^–1^ d^–1^, respectively.^54,63^ To evaluate its hydrolytic stability, **L-chn-1-Co-NO3** and **L-chn-1-Ni-NO3** were subjected to 100 sorption cycles (0–60% RH swing at 298 K, Figure 3e,f). Sorption capacity was retained over 100 cycles (loss **L-chn-1-Co-NO3** = 0.02 wt %; **L-chn-1-Ni-NO3** = 0.18 wt %), and crystallinity was retained after cycling (Figure S36).

To gain further insight into the water structure in the channels of **L-chn-1-Co-NO3** and **L-chn-1-Ni-NO3**, we performed a theoretical analysis using periodic density functional theory (DFT) and grand canonical Monte Carlo (GCMC) simulations (see Supporting Information for details). After initial DFT-optimization of the guest-free frameworks, water molecules were generated in accordance with their experimental loadings (see Supporting Information), followed by molecular dynamics calculations. Radial distribution function (RDF) analysis of the equilibrated frames shows that lattice water forms a conceptually infinite hydrogen-bonded network with modal oxygen–oxygen atom distances *r*(O_H2O_-O_H2O_) = 2.7 Å and *r*(O_H2O_-O_H2O_) = 2.8 Å for **L-chn-1-Co-NO3** and **L-chn-1-Ni-NO3**, respectively (Figure S44). The results of the simulations reveal that water molecules exhibit thermal motion about their average simulated positions but maintain a network structure sustained by hydrogen-bonding interactions (Movies S1 and S2). Similarly, the host frameworks exhibit thermal motion and undergo subtle adjustments in accordance with the clustering of water molecules, with the most notable movements being the rotation of the nitrate ligands about their coordination bonds. RDF analysis of the framework–water interactions reveals that water molecules form hydrogen bonds with the most exposed O_NO3_ atoms, O_NO3_’, in both **L-chn-1-Co-NO3** and **L-chn-1-Ni-NO3**, with *r*(H_H2O_-O_NO3_’) = 1.9 Å and *r*(H_H2O_-O_NO3_’) = 1.9 Å, respectively (Figure S46). The absence of any distinguishable peaks in the O_H2O_-H_aqua_, H_H2O_-O_aqua_, and O_H2O_-H_bipy_ RDFs indicates that water does not form strong interactions with the rest of the framework.

**Figure 4:**
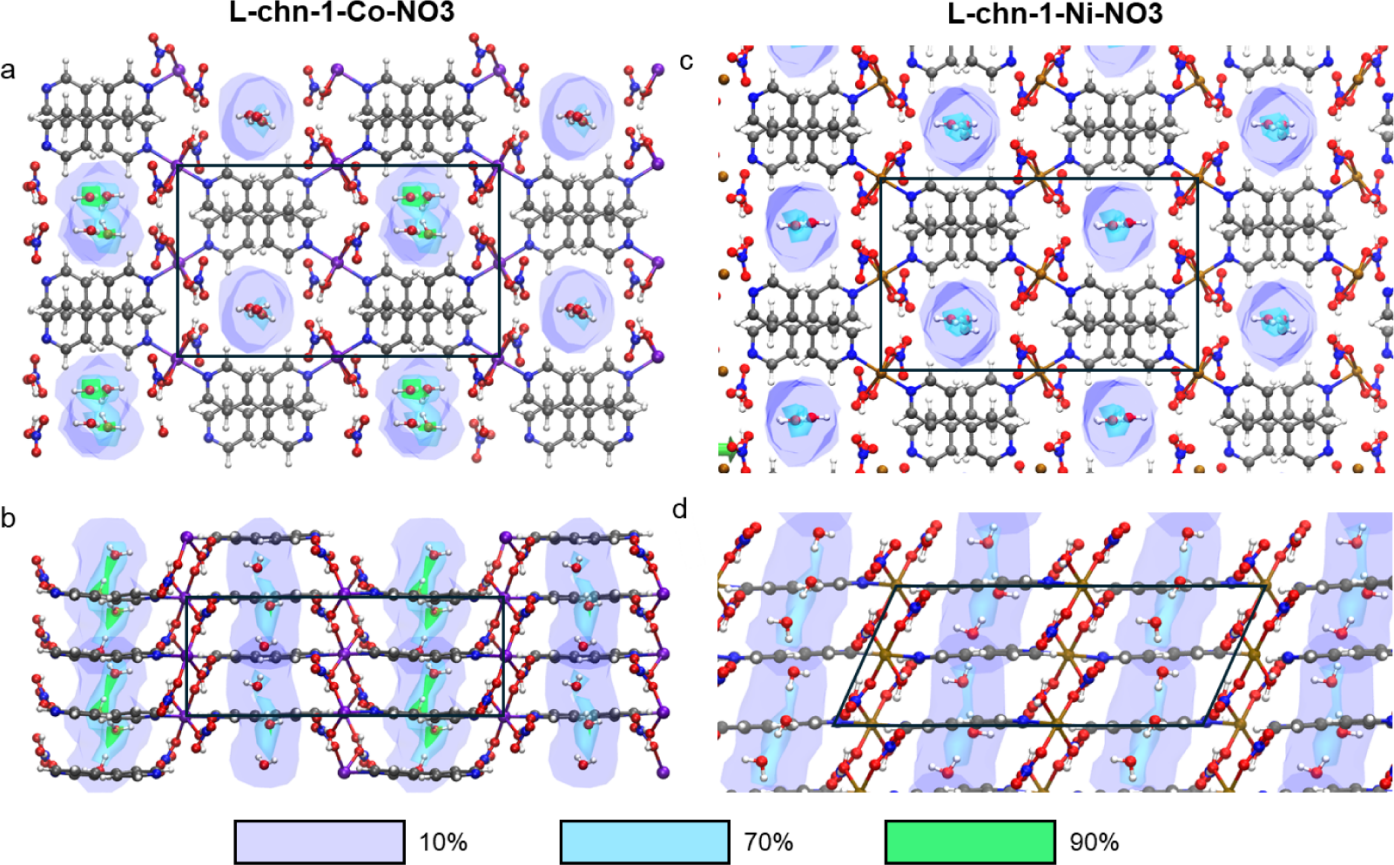
Structure from DFT simulations of the single unit cell with corresponding O_H2O_ occupancy maps of **L-chn-1-Co-NO3** along the (a) *c* axis and (b) *a* axis and **L-chn-1-Ni-NO3** along the (c) *c* axis and (d) *b* axis: fractional occupancies (0.1, blue; 0.7, cyan; 0.9, green) of channel water were calculated from equilibrated frames. Colors: oxygen, red; hydrogen, white; carbon, gray; nitrogen, purple; cobalt, brown; nickel.

Occupancy percentage distribution (OPD) maps (Figures 4 and S47–S49) were generated by determining how often water was found at specific coordinates, providing insight into the preferred locations in the channel. For both structures, the regions with the lowest OPD intensity (present only in 10% of the frames) are along the channel walls, therefore, suggesting weak framework–water interactions. Higher intensity peaks (70%) occur in the center of the channels but have smeared profiles, indicating that there is an array of possible locations, as opposed to discrete sorbate sites. Close inspection of the highest intensities (90%) reveal that these are located between the nearest O_NO3_’ atoms across the channel, i.e., near the centroids of the adjacent O_NO3_’ atoms (Figures 4 and S47–S49). Re-evaluation of the difference electron density maps from the crystal structures of **L-chn-1-Co-NO3** and **L-chn-1-Ni-NO3**, revealed that, while the electron densities are diffuse, the highest peaks are also situated near these centroids (Figures S53–S55). In the case of **L-chn-1-Ni-NO3**, the nearest O_NO3_’ atoms are situated across the channel such that the lines connecting their centers follow a zigzag pattern when viewed along [100]. The resultant difference electron density map has an undulating profile that traces this pattern. In the case of **L-chn-1-Co-NO3**, the centroids are more evenly distributed, with the most intense peaks also observed near these centroids. Additional localized peaks between these positions correspond to crystallographic disorder of water in **L-chn-1-Co-NO3** resulting from the close proximity of these favorable sites to one another; the centroid–centroid distances are 2.1 Å, much shorter than reasonable O_H2O_-O_H2O_ distances of 2.5–3.2 Å.^39^ While water–water interactions dominate in both **L-chn-1-Co-NO3** and **L-chn-1-Ni-NO3**, the difference in the distribution of hydrophilic sites in the crystal structures results in differing water cluster structures in the channels. It is likely that these differences are responsible for the slight but notable differences in the sorption properties of **L-chn-1-Co-NO3** and **L-chn-1-Ni-NO3**. The ease with which water molecules desorb from these structures can also be inferred from the difference electron density distribution and simulated frames. It is known that channel hydrates whose water clusters have local environments similar to bulk water or polymorphs of ice (most often observed in channel hydrates with pore diameters exceeding 20 Å^64^) will desorb less readily than those with less-ordered water clusters (similar to amorphous ice) and weak water–framework interactions.^61,65^ Owing to the geometric constraint of the channels imposed on the included water, it is feasible that the relative ease with which water is removed from **L-chn-1-Co-NO3** and **L-chn-1-Ni-NO3**, i.e., desorption under mild experimental conditions, is due to the nonordered structure of water that forms in their channels. In this regard, the slower desorption kinetics exhibited by **ROS-037** with respect to **L-chn-1-Co-NO3** and **L-chn-1-Ni-NO3** is possibly driven by the greater extent of water ordering in the **ROS-037** channels (Figure S56), as evidenced by reasonable O_H2O_-O_H2O_ distances (ca. 2.9 Å) in the reported^62^ crystal structure.

## Conclusions

1D CPs have been understudied as sorbents, perhaps because of their perceived low propensity to form robust, permanently porous structures. We herein report on the prototypical 1D HBPCP family **chn-1-M-NO3**, which is sustained by a trifecta of directional supramolecular interactions and is highly amenable to crystal engineering because of its inherent modularity. In terms of properties, **chn-1-M-NO3** exhibited water vapor sorption properties relevant to AWH: an S-shaped isotherm with a step at <30% RH; relatively fast adsorption and desorption kinetics; mild activation conditions; hydrolytic stability over 100 sorption cycles. A detailed structural and computational analysis of adsorbed water indicates that the sorption properties of **L-chn-1-Co-NO3** and **L-chn-1-Ni-NO3** are enhanced by the formation of a hydrogen-bonded network that features no long-range order during pore-filling/emptying. Despite relatively low surface areas (55 and 76 m^2^ g^–1^), the water harvesting productivities of **L-chn-1-Co-NO3** and **L-chn-1-Ni-NO3** were found to be comparable with several leading AWH desiccants. Overall, this work demonstrates not only that 1D HBPCPs can afford permanent porosity sustained by the directional nature of coordination bonds and two types of supramolecular synthons, but also that their properties are pertinent to and can provide insight into AWH.

## Supporting Information Available

Experimental details, crystallographic data, TGA, gas sorption, water vapor sorption, kinetic analysis, and molecular modeling (PDF)
(MP4)
(MP4)
(MP4)
(MP4)
(MP4)
(MP4)
(MP4)
(MP4)
(MP4)
(MP4)
