# Supplementary material for: An Ultramicroporous Physisorbent Sustained by a Trifecta of Directional Supramolecular Interactions
Source: J Am Chem Soc. 2025 Jan 2;147(2):1813–22. doi: 10.1021/jacs.4c13797 (PMC11744747; doi:10.1021/jacs.4c13797)
Supplement: Supplementary file 1 — ja4c13797_si_001.pdf [file ja4c13797_si_001.pdf]

An ultramicroporous physisorbent sustained by a trifecta of directional supramolecular interactions

Alan C. Eaby,[†] Shaza Darwish,[†] Shi-Qiang Wang,[†] Andrey A. Bezrukov,[†] Debobroto Sensharma,[†] Angela Shipman,[‡] Carlos Solanilla,[‡] Brian Space,[‡] Soumya Mukherjee,[†] Michael J. Zaworotko*[,†]

[†]Bernal Institute and Department of Chemical Sciences, University of Limerick, Limerick V94 T9PX, Ireland

[‡] Department of Chemistry, North Carolina State University, Raleigh, NC 27607, USA

*Email: xtal@ul.ie

Table of contents

1 Materials and Methods 3

1.1 Materials 3

1.2 Synthetic Procedures 3

1.3 Single Crystal X-ray Diffraction 3

1.4 Crystallographic Software 3

1.5 Powder X-ray Diffraction 3

1.6 Variable-Temperature Powder X-ray Diffraction 4

1.7 Thermal Analysis 4

1.8 Gas Sorption 4

1.9 Dynamic Vapor Sorption 5

1.9.1 Water Vapor Sorption Isotherms 5

1.9.2 Water Vapor Sorption Kinetic Analysis 5

1.9.3 Water Sorption Performance Heatmaps 6

1.9.4 Water Vapor Sorption Hydrolytic Stability (cycling) 6

2 Single-Crystal X-Ray Diffraction Analysis of **L-chn-1-Co-NO3** and **L-chn-1-Ni-NO3** 10

2.1 Crystal Structure Data of **L-chn-1-Co-NO3** and **L-chn-1-Ni-NO3** at –173 and 25 °C 10

2.2 Pore volumes, occupancy of H_2O molecules and possible structure of lattice water in **L-chn-1-Co-NO3** and **L-chn-1-Ni-NO3** at –173 and 25 °C 11

3 Estimation of Adsorption Enthalpy 25

3.1 Estimation of Adsorption Enthalpy using Clausius-Clapeyron Approach 25

3.2 Estimation of Adsorption Enthalpy using Differential Scanning Calorimetry 28

4 Molecular Modelling 44
4.1 Density Functional Theory 44
4.1.1 Spin State Determination of Co Atoms 44
4.1.2 Cell Optimization 45
4.1.3 Molecular Dynamics 46
4.1.4 Radial Distribution Function Analysis 50
4.1.5 Occupancy percentage distribution maps 54
4.2 Monte Carlo 58
5 Crystallographic Evaluation of the Favored Water Sorption Sites in **L-chn-1-Co-NO3** and **L-chn-1-Ni-NO3** 62
6 Captions for Supplementary Movies 66
References 67

1 Materials and Methods

1.1 Materials

All reagents and solvents were used as received from commercial sources without further purification.

1.2 Synthetic Procedures

Single crystals of **L-chn-1-Co-NO3** and **L-chn-1-Ni-NO3** were obtained by layering the metal nitrate salt and 4,4'-bipyridine in EtOH following a known literature procedure.[1] Bulk samples were synthesized by placing equimolar amounts of 4,4′-bipyridine (*ca.* 156 mg, 1 mmol) and a metal nitrate (*ca*. 291 mg, 1 mmol) into a 20 mL vial with 5 mL of EtOH. The slurry was stirred continuously for 1 h prior to filtering the suspended solid. Microcrystalline powder of **ROS-037** was synthesized using a known literature procedure.[2]

1.3 Single Crystal X-ray Diffraction

High-quality single crystals of **L-chn-1-Co-NO3** and **L-chn-1-Ni-NO3** were chosen for single-crystal X-ray diffraction measurements and diffraction data were collected on Bruker Quest diffractometer equipped with a IμS microfocus X-ray source (Mo Kα, λ = 0.71073 Å, Cu Kα, λ = 1.54178 Å) and CMOS detector. Data reduction was carried out using the Bruker SAINT[3] software. Absorption correction was performed by multi-scan method using in SADABS.[4] Space group determination was performed simultaneously with structure solution using SHELXT[5] intrinsic phasing methods through the X-Seed[6] graphical user interface. Atoms of the host were refined anisotropically using SHELXL,[7] using full-matrix least squares minimization. See Text S2.2 for details on modelling of included water.

1.4 Crystallographic Software

The Cambridge Structural Database[8] (CSD, v.5.45; database updates: Mar and Jun 2024) was accessed using ConQuest.[9] Pore volumes and geometries (pore limiting diameters and maximum pore diameters) were calculated using the Pore Analyser (default settings) feature in Mercury[10] (2023.3.0, Build 392256). Crystal structure models and void volumes were visualized using Mercury[10] unless otherwise specified. Difference electron density maps were calculated using OLEX2.[11]

1.5 Powder X-ray Diffraction

Powder X-ray diffraction (PXRD) experiments were carried out on microcrystalline samples using a PANalytical Empyrean diffractometer (40 kV, 40 mA, Cu $K_{\alpha1,2}$, λ = 1.5418 Å) in Bragg Brentano geometry. Instrument details: reflection transmission spinner configuration, divergence slit = 1/8°, Soller slit = 0.04 rad, Ni filter, PIXcel-1D detector. Data was collected from 5 to 60˚ 2θ with a step size of 0.0262606˚ and

a scan time of 96 seconds per step. Powder samples were evenly distributed on a glass sample holder after being ground with a mortar and pestle to minimize the effects of preferred orientation. Data analysis was carried out using X'Pert HighScore Plus (Version 2.2e).[12] Reference powder patterns were calculated from SCXRD structures using Mercury.[10]

1.6 Variable-Temperature Powder X-ray Diffraction

Diffractograms at different temperatures were recorded using a PANalytical X'Pert Pro-MPD diffractometer equipped with a PIXcel3D detector operating in scanning line detector mode. Anton Paar TTK 450 stage coupled with the Anton Paar TCU 110 Temperature Control Unit was used to record the variable temperature diffractograms. The diffractometer is outfitted with an Empyrean Cu LFF (long fine focus) HR (9430 033 7300x) tube operated at 40 kV and 40 mA and Cu K_α radiation (λ_α = 1.54056 Å) was used for diffraction experiments. Continuous scanning mode with the goniometer in the theta-theta orientation was used to collect the data. Incident beam optics included the Fixed Divergences slit, with a 1/4° divergence slit and a Soller slit (0.04 rad). Divergent beam optics included a P7.5 S7 anti-scatter slit, a Soller slit (0.04 rad), and a Ni-β filter. In a typical experiment, 15 mg of sample was ground into a fine powder and was loaded on a sample holder made for Anton Paar TTK 450 chamber. The data was collected from 5°-60° (2θ) with a step-size of 0.0167113° and a scan time of 150 seconds per step. Data were analyzed using the X'Pert HighScore Plus (Version 2.2e).[12] It should be noted that the blank diffractogram i.e. that measured without sample (Figure S9), has peaks that can be observed in the VT-PXRD patterns.

1.7 Thermal Analysis

Thermogravimetric analysis (TGA) was carried using a TA Instruments Q50 system with a standard furnace type. Each sample was equilibrated under ambient conditions (ca 55% RH, 22 °C) loaded into an aluminum pan and heated at 10 °C min^{-1} from room temperature to 500 °C under a continuous flow of N_2 gas.

1.8 Gas Sorption

For CO_2 gas sorption experiments, ultrahigh-purity CO_2 (99.995%) was used as received from BOC Gases Ireland. A Micromeritics 3Flex surface area and pore size analyzer 3500 was used for collecting the 25 and −78 °C sorption isotherms of **L-chn-1-Co-NO3**, **L-chn-1-Ni-NO3**, **ZZ-chn-1-Co-NO3** and **HT-Ni**. Before sorption measurements, two samples of **L-chn-1-Co-NO3** and **L-chn-1-Ni-NO3** were activated on a SmartVacPrep™ using dynamic vacuum either at 25 °C, giving the porous guest-free phases of **L-chn-1-Co-NO3** (dry mass 75.3 mg) and **L-chn-1-Ni-NO3** (dry mass 49.4 mg), and at 110 °C, yielding **ZZ-chn-1-Co-NO3** (dry mass 91.0 mg) and **HT-Ni** (dry mass 62.4 mg). A Julabo temperature controller was used to maintain a constant temperature in the bath throughout the experiment. The Brunauer, Emmett and Teller (BET)[13] surface areas of **L-chn-1-Co-NO3** and **L-chn-1-Ni-NO3** were calculated from CO_2 sorption data

measured at 195 K and were determined to be 15.3 and 89.2 m^2 g^{-1}, respectively. However, considering that BET is not appropriate for ultramicroporous materials[14] the surface areas were determined geometrically in Mercury[10] (Text S2.2).

1.9 Dynamic Vapor Sorption

1.9.1 Water Vapor Sorption Isotherms

All water vapor sorption analyses were carried out using an Adventure Dynamic Vapour Sorption (DVS) instrument (Surface Measurement Systems), unless specified otherwise. A total flow rate of 400 sccm (standard cubic centimeters per minute) was maintained for the two branches of the microbalance. Powdered samples of **L-chn-1-Co-NO3** (dry mass 16.75 mg) and **L-chn-1-Ni-NO3** (dry mass 13.22 mg) were loaded onto the balance pan of an analyzer cell and initially activated at 0% RH and 40 °C for 2 h. Three consecutive ad/desorption isotherms (Cycles 1–3) were measured using the following sequence: Cycle 1: the sample was activated at 0% RH at 25 °C for 2 h (initial activation); Cycle 2: the sample was activated at 0% RH at 150 °C for 2 h and equilibrated at 25 °C for 2 h; Cycle 3: the sample was activated at 0% RH at 25 °C for 2 h. In a typical experiment and following the completion of activation, the sample compartment was equilibrated to 25 °C and the RH was increased from a target value of 0% to 90% in with RH step sizes ranging from 1–5%, and then decreased to 0% with similar RH steps. During each step, the RH was maintained until the mass change (dm/dt) was < 0.01 wt% min^{-1}. The volumetric uptake (g_{H_2O} cm^{-3}_{MOF}) was calculated from gravimetric DVS data using the crystallographic density guest-free structures of **L-chn-1-Co-NO3** (1.471 g cm^{-3}, CSD Refcode: FOGGUF) and **L-chn-1-Ni-NO3** (1.504 g cm^{-3}, CSD Refcode: FOGGIT). These were found to be 106 and 107 g cm^{-}3 for **L-chn-1-Co-NO3** and **L-chn-1-Ni-NO3**, respectively.

1.9.2 Water Vapor Sorption Kinetic Analysis

Kinetic measurements were carried out on a 50-100 μm sample fraction obtained by sieving. Dry sample masses of 11.068 mg, 11.0059 mg and 10.9415 mg were used for **L-chn-1-Co-NO3**, **L-chn-1-Ni-NO3** and **ROS-037**, respectively. 400 sccm min^{-1} flow was once again used for these measurements. For adsorption and desorption kinetic curves, dm/dt < 0.01 % min^{-1} was used as criteria of reaching equilibrium. Kinetics were compared by using the isotherm-based kinetics model[2] making use of Equation S1:

$$\frac{\mathrm{d}w}{\mathrm{d}t} = k \cdot (RH_{\mathrm{flow}} - RH_{\mathrm{bed}}) \quad \text{[S1]}$$

where w is uptake (wt.%), k is sorption coefficient, RH_{flow} is relative humidity in the flow and RH_{bed} is relative humidity in sample bed. Adsorption and desorption kinetics was modelled using Equation S1. RH_{bed} was determined from adsorption branch of the isotherm at the corresponding uptake. Two parameters were fitted: k and t_0, where t_0 was fitted in 0-3 minutes range.

1.9.3 Water Sorption Performance Heatmaps

Water sorption performance heatmaps were calculated using method adapted from Bezrukov *et al*.[2] The sorption kinetics of **L-chn-1-Co-NO3**, **L-chn-1-Ni-NO3** and **ROS-037** was modelled via isotherm-based kinetics model using k = 0.3, m_{sample} = 10 mg and adsorption branch of the isotherm as input parameters. Adsorption-desorption cycling in 0–30 % RH humidity swing was calculated for times in a range 1–50 min with 1 min step size.

1.9.4 Water Vapor Sorption Hydrolytic Stability (cycling)

Water vapor sorption cycling was performed at 25 °C on a DVS Intrinsic gravimetric instrument (Surface Measurement Systems) using dry air as a carrier gas. The mass of the sample was determined by comparison to an empty reference pan and recorded by a high-resolution microbalance with a precision of 0.1 µg where dry masses of 13.996 mg and 10.418 mg were used for **L-chn-1-Co-NO3** and **L-chn-1-Ni-NO3**, respectively. Prior to the measurement, a sample was activated *in situ* in dry air at 40 °C for 6 h. A total of 100 humidity swing cycles were carried out with each cycle consisting of an adsorption step (target RH = 60 % RH) and a desorption step (target RH = 0 % RH) where the target RH was maintained for a fixed period: target RHs were maintained for 20 min and 30 min for **L-chn-1-Co-NO3** and **L-chn-1-Ni-NO3**, respectively.

Table S1 Permanently porous 1D CPs reported in the literature and selected geometric and sorption properties.

As-synthesized composition	Structural Motif†	Chain packing arrangement	Pore dimension	Pore limiting diameter (Å)	Maximum pore diameter (Å)	Key interactions (excluding coordination bond)	Sorption	CSD Refcode	Year	Reference
**Hydrogen-bonded PCPs**										
$[Cd(bpea)(phen)(H_2O)]_n \cdot nH_2O$	Zig-zag	Polythreaded	1D	3.59*	4.24*	HB, π–π, CH-π	None	PEPTIO	2006	15
$[Zn(H_2L^1)(L^2)]\cdot DMF\cdot 2H_2O$	Ribbon/ Fishbone	Collinear (linked intrinsic pores)	1D	3.28	4.96	HB, π–π	CO_2 (273 K)	VIWTUS	2014	16
$Ni(NCS)_2(L^3)_2\cdot MeCN$	Ribbon	Collinear (linked intrinsic pores)	1D	2.54	4.15	HB	N_2 (77 K)	SAWKUZ	2017	17
L-chn-1-Co-NO3	Chain	Slanted (ABAB, 120°)	1D	3.8	4.6	HB, π–π	CO_2 (195 K & 298 K), H_2O (298 K)			This work
L-chn-1-Ni-NO3				3.6	4.5					This work
**Permanently Porous 1D CPs**										
$[Ni(cyclam)(bpydc)]\cdot 5H_2O$	Chain	Slanted (ABCABC, 120°)	1D	5.99	7.39	C-H–π	N_2, H_2 (77 K)	BEVYUX	2004	18
$CdL^4{}_2(ClO_4)_2\cdot 11EtOH\cdot 6H_2O$	Ribbon	Slanted (ABCABC, 120°)	1D	8.76*	10.28*	π–π	CO_2 (273 K)	LAKHAH	2005	19
$[NiL^4{}_2Cl_2]\cdot 3EtOH\cdot 4H_2O$	Ribbon	Slanted (ABCABC, 120°)	3D	4.13	6.74	π–π	CO_2 (273 K)	XEPMIP	2006	20
$Zn(phen)(L^5)\cdot DMSO$§	Zig-zag	Polythreaded (entangled warp-and-woof weaves)	1D	3.77	5.02	π–π	None	PETTAK	2007	21
$[Cu(2\text{-}pySO_3)_2(bipy)]\cdot 5H_2O$	Chain	Collinear	1D	3.87*	4.14*	C-H-π, C-H-O-SO3	None‡	DOGJUF	2008	22
$Zn(phen)(L^5)\cdot DMSO$§	Zig-zag	Polythreaded (entangled warp-and-woof weaves)	1D	3.77	5.02	π–π	H_2 (77 K & 87 K)	VOCSUC	2010	23
$[Ni(TAU)_2(bipy)]\cdot 2H_2O$	Helical	Collinear (helices)	1D	3.21*	3.55*	π–π	None	OYEJUY	2010	24
$[Ag(BIPS)]_{0.5}[Ag(BIPS)]_2 \cdot 2.5ClO_4\cdot 5H_2O$	Zig-zag	Polythreaded (entangled warp-and-woof weaves)	1D	3.15*	5.37*	π–π	None	NUVGUH	2010	25

Table S1 continued

As-synthesized composition	Structural Motif[†]	Chain packing arrangement	Pore dimension	Pore limiting diameter (Å)	Maximum pore diameter (Å)	Key interactions (excluding coordination bond)	Sorption	CSD Refcode	Year	Reference
[Ni(cyclam)Pd(2,4-pydc)$_2$·4H_2O[‡]	Chain	Slanted (ABAB)	1D	NA	NA	C-H–π	CO_2, N_2, CH_4 (273 K & 298 K)	NA	2013	26
[Zn(cyclam)Pd(2,4-pydc)2·4H_2O[‡]	Chain	Slanted (ABAB)	1D	5.14	6.13	C-H–π	CO_2, N_2, CH_4 (273 K & 298 K)	FEPKOC		
Co(cyclam)]$_2$[Ni(2,5- pydc)$_3$] ·xH_2O	Ladder	Collinear	1D	2.91*	3.88*	C-H–π	CO_2 (273 K & 298 K)	FEPKUI		
Ni(cyclam)]$_2$[Ni(2,5- pydc)$_3$] ·xH_2O	Ladder	Collinear	1D	2.73*	3.86*	C-H–π	CO_2, N_2, CH_4 (273 K & 298 K)	FEPLAP		
Zn(cyclam)]$_2$[Ni(2,5- pydc)$_3$] ·xH_2O	Ladder	Collinear	1D	2.90*	3.87*	C-H–π	CO_2 (273 K & 298 K)	FEPLET		
Ni(cyclam)]$_2$[Zn(2,5- pydc)$_3$] ·xH_2O	Ladder	Collinear	1D	2.80*	3.98*	C-H–π	CO_2 (273 K & 298 K)	FEPLIX		
Zn(cyclam)]$_2$[Zn(2,5- pydc)$_3$] ·xH_2O	Ladder	Collinear	1D	2.78*	3.93*	C-H–π	CO_2 (273 K & 298 K)	FEPLOD		
[NiL^6][Ni(CN)$_4$]·3H_2O	Chain	Slanted (ABCABC, 120°)	1D	6.27	7.84	VdW	CO_2 (195 K)	YUBHEK	2015	27
[Cd(NDI-py)(OH_2)$_4$](NO_3)$_{1.3±0.1}$ ·nDMA	Chain	Slanted (ABCABC, 120°)	1D	5.07	5.62	π–π	None	JOGYOW	2019	28

*Calculated from reported structure after solvent removed *in silico*

[†]According to ref [29]

[⸸]The sorption isotherms reported contain inflections indicating structural flexibility during guest ad/desorption. Reported PXRD of the hydrate matches the anhydrate.

[‡]Reported as isostructural to other analogues in ref [26]

[§]Isostructural, structures determined from SCXRD data collected at 298 and 223 K for ref [21] and ref [23], respectively

Abbreviations: **bpea** = biphenylethene-4,4'-dicarboxylate; **phen** = 1,10-phenanthroline; cyclam = 1,4,8,11-tetraazacyclotetradecane; **bpydc^{2-}** = 2,2'-bipyridyl-5,5'-dicarboxylate; **2-pySO$_3$** = 2-pyridine-sulfonate; **bipy** = 4,4′-bipyridine; **TAU** = taurine; **BIPS**= bis(4-(imidazol-1-yl)phenyl)dimethylsilane; **2,4-H$_2$pydc** = pyridine-2,4-dicarboxylate acid; **NDI-py** = N,N′-di(4-pyridyl)-1,4,5,8-naphthalenetetracarboxdiimide; **H$_4$L^1** = tetracarboxydiphenylmethane; **L^2** = 3,6-di-pyridin-4-yl-[1,2,4,5]tetrazine; **L^3** = 4-(Boc-amino)pyridine; **L^4** = (*S*)-2,2'-diethoxy-1,1'-binaphthyl-6,6'-bis(4-vinylpyridine); **H$_2$L^5** = trans-4,40 - stilbenedicarboxylic acid; **L^6** =1,4,7,9,12,14-hexaazatricyclo[12.2.1.1^{4,7}]octadecane

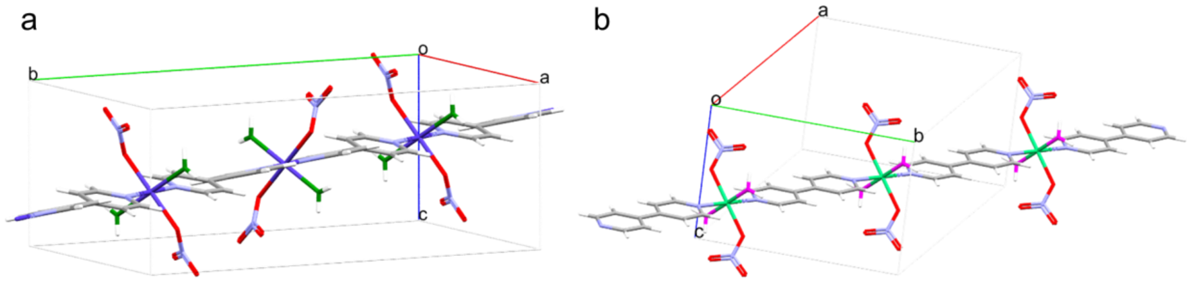

Figure S1 Comparison of the orientation of the MBB of (a) **L-chn-1-Co-NO3**, also observed for **L-chn-1-M-NO3** analogs (M = Fe, Cu, Zn), and (b) **L-chn-1-Ni-NO3**. Aqua ligand oxygen atoms are shown in green (a) and magenta (b). Colors: carbon, gray; nitrogen, blue; oxygen, red; hydrogen, white; cobalt, purple; nickel, light green.

2 Single-Crystal X-Ray Diffraction Analysis of **L-chn-1-Co-NO3** and **L-chn-1-Ni-NO3**

2.1 Crystal Structure Data of **L-chn-1-Co-NO3** and **L-chn-1-Ni-NO3** at –173 and 25 °C

Crystal data for **L-chn-1-Co-NO3** at –173 °C**:** $C_{10}H_{12}CoN_4O_{10.04}$, *M* = 407.77, orange block, 0.265 × 0.150 × 0.142 mm^3, orthorhombic, space group *Pccn* (No. 56), *a* = 11.7388(11), *b* = 19.4810(17), *c* = 7.3784(6) Å, *V* = 1687.3(3) Å^3, *Z* = 4, D_c = 1.605 g cm^{-3}, F_{000} = 829, MoKa radiation, l = 0.71073 Å, *T* = 100(2) K, $2q_{max}$ = 56.7º, 47484 reflections collected, 2106 unique (R_{int} = 0.0449). Final *GooF* = 1.115, *R*1 = 0.0363, *wR*2 = 0.0842, *R* indices based on 1850 reflections with *I* > 2s(*I*) (refinement on F^2), 140 parameters, 0 restraints. Lp and absorption corrections applied, *m* = 1.076 mm^{-1}.

Crystal data for **L-chn-1-Co-NO3** at 25 °C**:** $C_{10}H_{12}CoN_4O_8$, *M* = 375.17, orange block, 0.162 × 0.156 × 0.064 mm^3, orthorhombic, space group *Pccn* (No. 56), *a* = 11.8178(3), *b* = 19.4851(5), *c* = 7.4024(2) Å, *V* = 1704.56(8) Å^3, *Z* = 4, D_c = 1.462 g cm^{-3}, F_{000} = 764, Bruker APEX-II CCD, MoKα radiation, λ = 0.71073 Å, *T* = 298(2) K, $2\theta_{max}$ = 54.2º, 23620 reflections collected, 1885 unique (R_{int} = 0.0450). Final *GooF* = 1.146, *R*1 = 0.0564, *wR*2 = 0.1553, *R* indices based on 1614 reflections with *I* > 2σ(*I*) (refinement on F^2), 114 parameters, 0 restraints. Lp and absorption corrections applied, μ = 1.049 mm^{-1}.

Crystal data for **L-chn-1-Ni-NO3** at –173 °C**:** $C_{10}H_{12}N_4NiO_{9.66}$, *M* = 401.55, blue block, 0.157 × 0.094 × 0.080 mm^3, monoclinic, space group *C*2/*c* (No. 15), *a* = 19.3449(9), *b* = 11.5729(5), *c* = 7.3774(3) Å, β = 97.466(2)°, *V* = 1637.62(12) Å^3, *Z* = 4, D_c = 1.629 g cm^{-3}, F_{000} = 821, CuKα radiation, λ = 1.54178 Å, *T* = 446(2) K, $2\theta_{max}$ = 140.0º, 17073 reflections collected, 1552 unique (R_{int} = 0.0522). Final *GooF* = 1.122, *R*1 = 0.0349, *wR*2 = 0.0900, *R* indices based on 1464 reflections with *I* > 2σ(*I*) (refinement on F^2), 129 parameters, 0 restraints. Lp and absorption corrections applied, μ = 2.271 mm^{-1}.

Crystal data for **L-chn-1-Co-NO3** at 25 °C**:** $C_{10}H_{12}N_4NiO_8$, *M* = 374.95, blue block, 0.121 × 0.077 × 0.061 mm^3, monoclinic, space group *C*2/*c* (No. 15), *a* = 19.3283(6), *b* = 11.6882(4), *c* = 7.4137(3) Å, β = 94.488(2)°, *V* = 1669.72(10) Å^3, *Z* = 4, D_c = 1.492 g cm^{-3}, F_{000} = 768, CuKα radiation, λ = 1.54178 Å, *T* = 298(2) K, $2\theta_{max}$ = 136.2º, 10293 reflections collected, 1520 unique (R_{int} = 0.0539). Final *GooF* = 1.171, *R*1 = 0.0830, *wR*2 = 0.2496, *R* indices based on 1394 reflections with *I* > 2σ(*I*) (refinement on F^2), 102 parameters, 0 restraints. Lp and absorption corrections applied, μ = 2.106 mm^{-1}.

2.2 Pore volumes, occupancy of H_2O molecules and possible structure of lattice water in **L-chn-1-Co-NO3** and **L-chn-1-Ni-NO3** at –173 and 25 °C

After all the atoms of the host were modelled and refined to convergence, residual electron density was observed in the difference electron density map. The number of electrons in the channels of the crystals structures modelled from −173 and 25 °C data was determined using PLATON[30] SQUEEZE[31] using a 1.2 Å probe radius: Solvent accessible volumes (SAV) and electron density counts were consistent with TGA and sorption data: **L-chn-1-Co-NO3**: SAV (–173 °C) = 335 $Å^3$ with 76 $e^–$ found; SAV (25 °C) = 345 $Å^3$ with 51 $e^–$ found; **L-chn-1-Ni-NO3**: SAV (–173 °C) = 303 $Å^3$ with 55 $e^–$ found; SAV (25 °C) = 312 $Å^3$ with 40 $e^–$ found;. It is likely that the lower relative electron count found in the crystal structure determined from data collected at 25 °C is due more diffuse electron density and possibly partial desorption that occurred under the dry nitrogen stream.

The difference electron density maps indicate that lattice water is located throughout the channel and possibly forms a conceptually infinite hydrogen-bonded network. To evaluate the structure of water in the channels of **L-chn-1-Co-NO3** and **L-chn-1-Ni-NO3**, significant peaks (>0.5 $e^- Å^{-3}$) were modelled as H_2O oxygen atoms (Figure S3), labelled OW*n* (*n* = 1–6 for **L-chn-1-Co-NO3** and *n* = 1–3 for **L-chn-1-Ni-NO3**), and the final site-occupancy factor (S. O. F.) determined sequentially by constraining the atomic displacement parameter to 0.14 $Å^2$ (i.e., the U_{eqiv} value of coordinated oxygen atoms) and an unconstrained S. O. F. of 0.5. **L-chn-1-Co-NO3:** The final S. O. F.s were found to be 0.33, 0.19, 0.14, 0.16, 0.10 and 0.10 for OW1-OW6, respectively giving a total of 2 O_{water}/ASU. **L-chn-1-Ni-NO3:** The final S. O. F.s were found to be 0.23, 0.38 and 0.22 for OW1-OW3, respectively, giving a total of 0.83 O_{water}/ASU. Low S. O. F.s indicate that water molecules are positionally disordered. While it is possible to construct a hydrogen-bonded network structure from the modelled oxygen atoms, it is challenging to suggest the most probable hydrogen-bonded network structure.

L-chn-1-Co-NO3 has a pore limiting diameter of d_{min} = 3.8 Å and maximum cavity diameter d_{max} = 4.6 Å, whereas **L-chn-1-Ni-NO3** has d_{min} = 3.6 Å and d_{max} = 4.5 Å. The total surface areas of **L-chn-1-Co-NO3** and **L-chn-1-Ni-NO3** were calculated geometrically using the Pore Analyser feature in Mercury[10] and were found to be 55 and 76 $m^2 g^{-1}$, respectively.

L-chn-1-Co-NO3

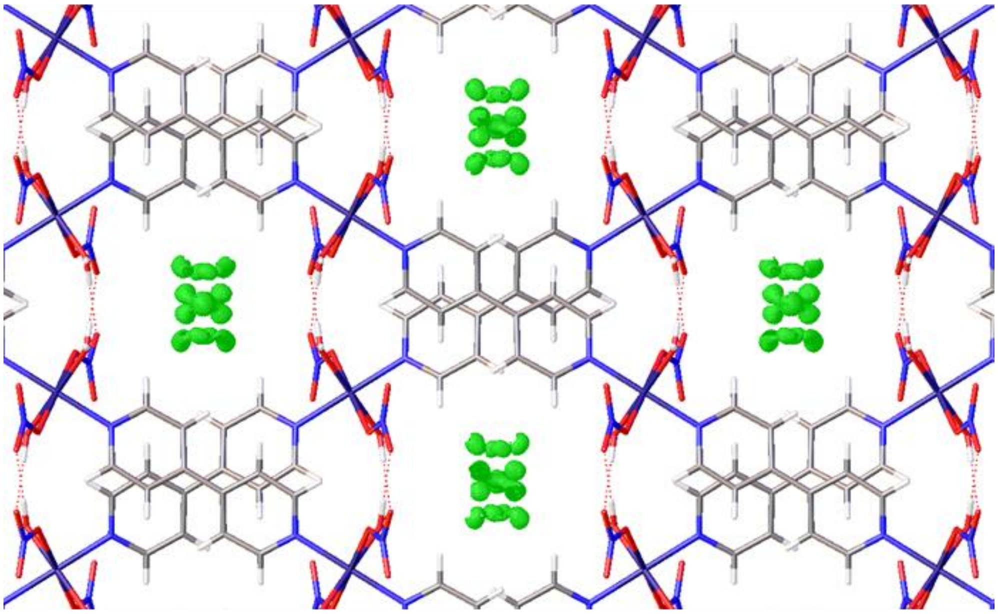

L-chn-1-Ni-NO3

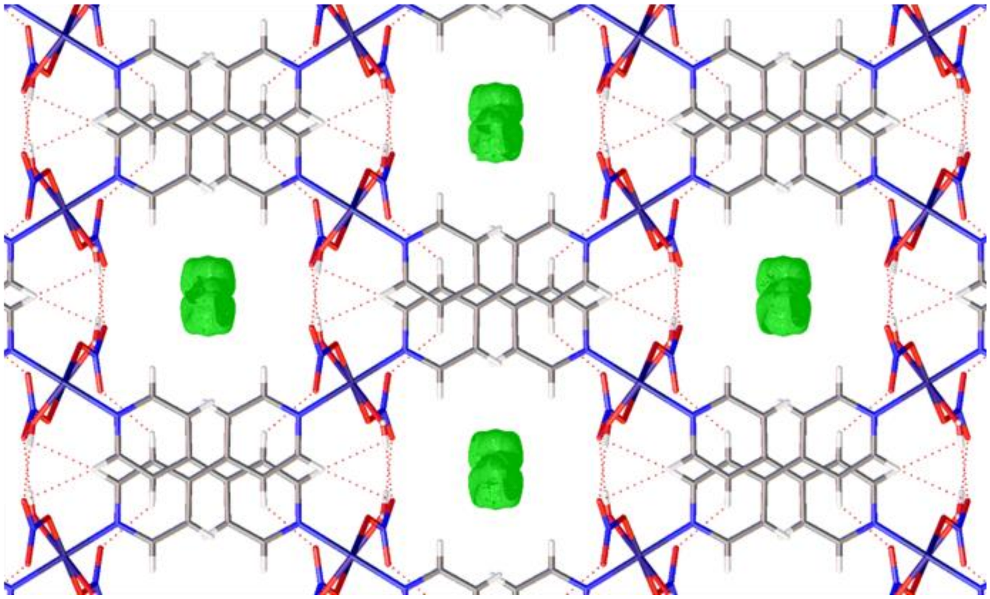

Figure S2 Projections of **L-chn-1-Co-NO3** (top) and **L-chn-1-Ni-NO3** (bottom) along [001] determined from refined crystal structures at –173 °C. Difference electron density maps (0.75 e^{-} $Å^{-3}$, green) calculated using OLEX2.[11] Colors: carbon, gray; nitrogen, blue; oxygen, red; hydrogen, white; metal atoms, dark blue.

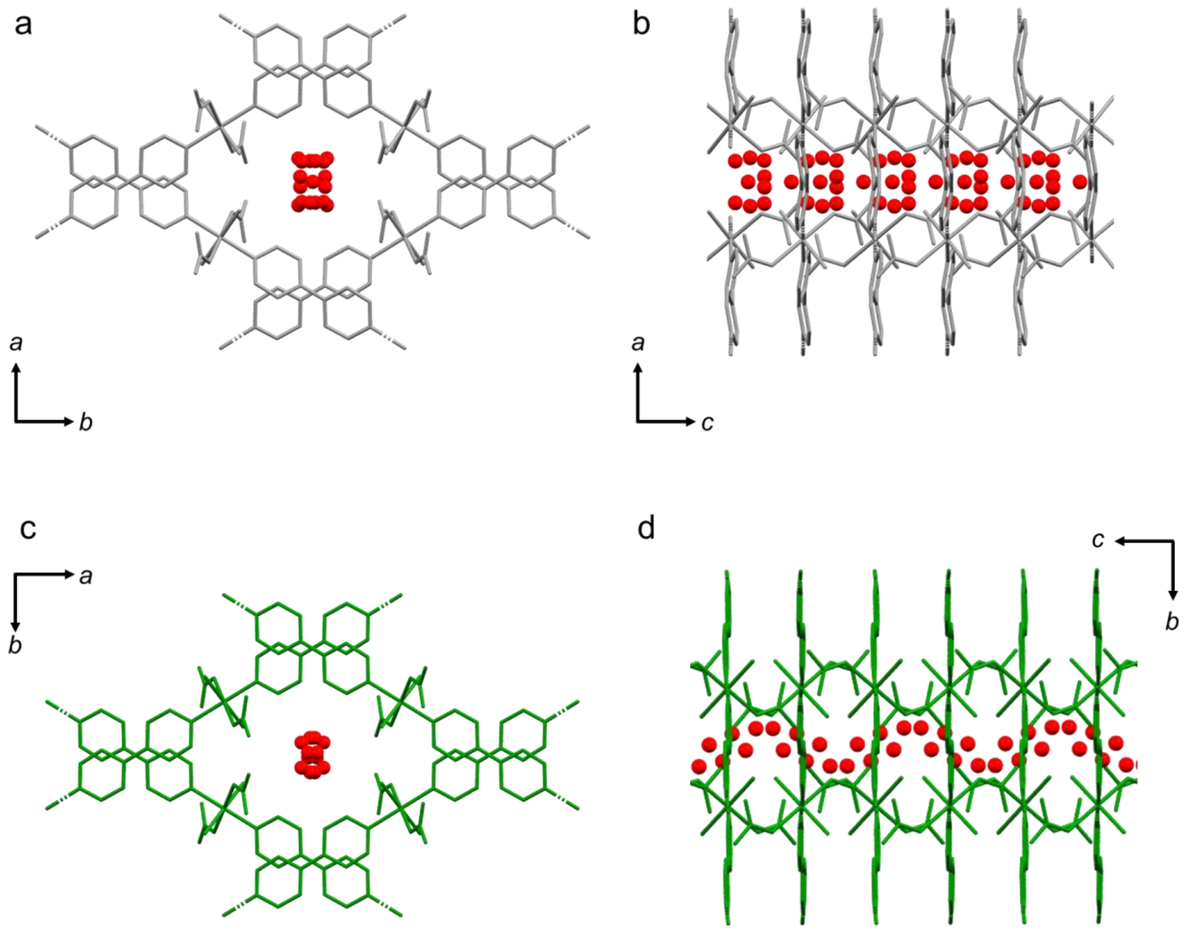

Figure S3 Projections along (a) [001] and (b) [010] for **L-chn-1-Co-NO3** and along (c) [001] and (d) [100] for **L-chn-1-Ni-NO3** modelled from SCXRD data measured at −173 °C. **L-chn-1-Co-NO3** host framework atoms (gray, capped sticks), **L-chn-1-Ni-NO3** host framework atoms (green, capped sticks) and H_2O oxygen atoms (red, ball-and-stick) are shown. Hydrogen atoms have been omitted for clarity.

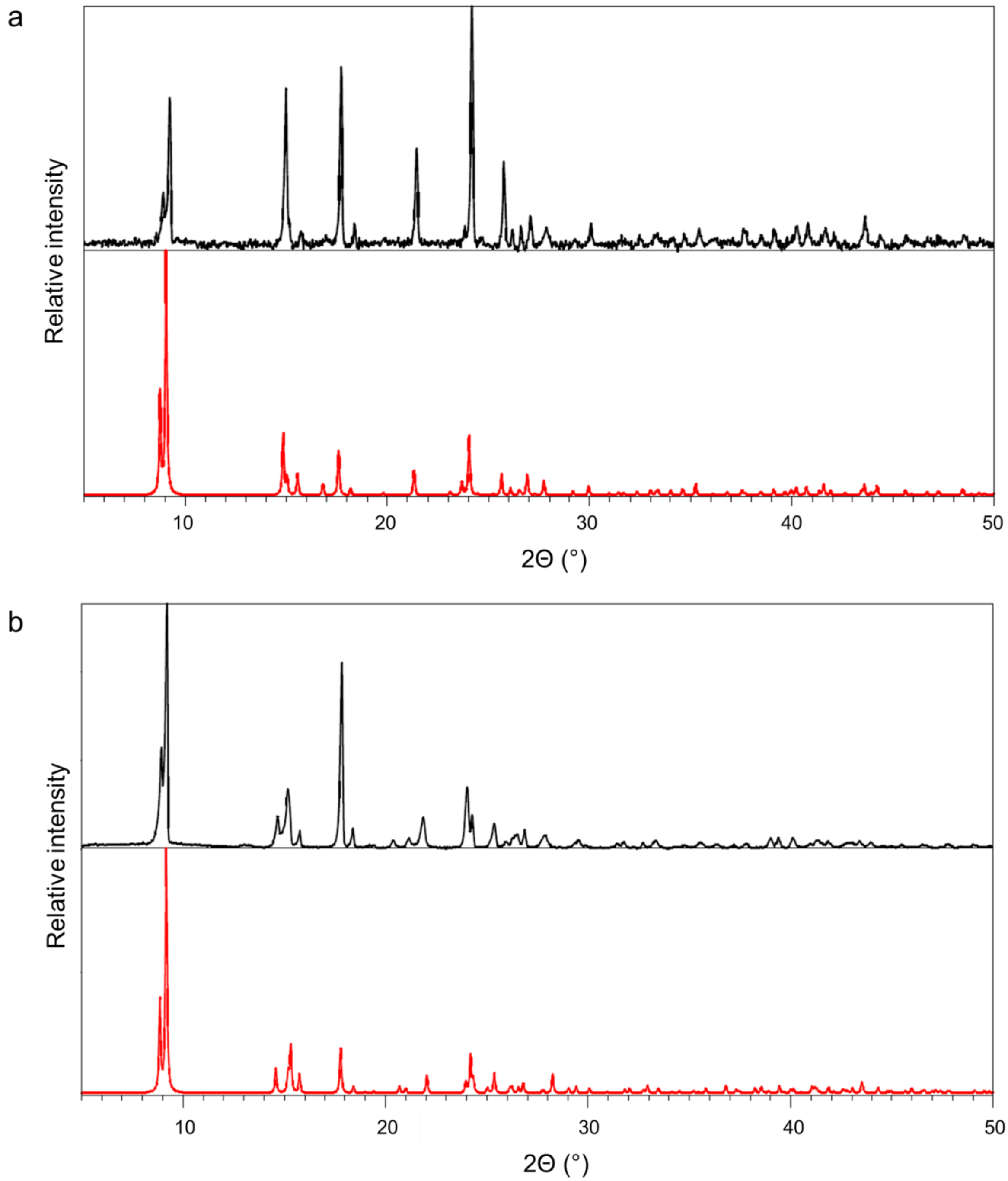

Figure S4 Powder X-ray diffractograms of (a) **L-chn-1-Co-NO3** and (b) **L-chn-1-Co-NO3** obtained from slurry (black) and calculated (red) from a crystal structure retrieved from the CSD using Mercury[12] Refcodes: (a) FOGGIT, (b) FOGGUF.

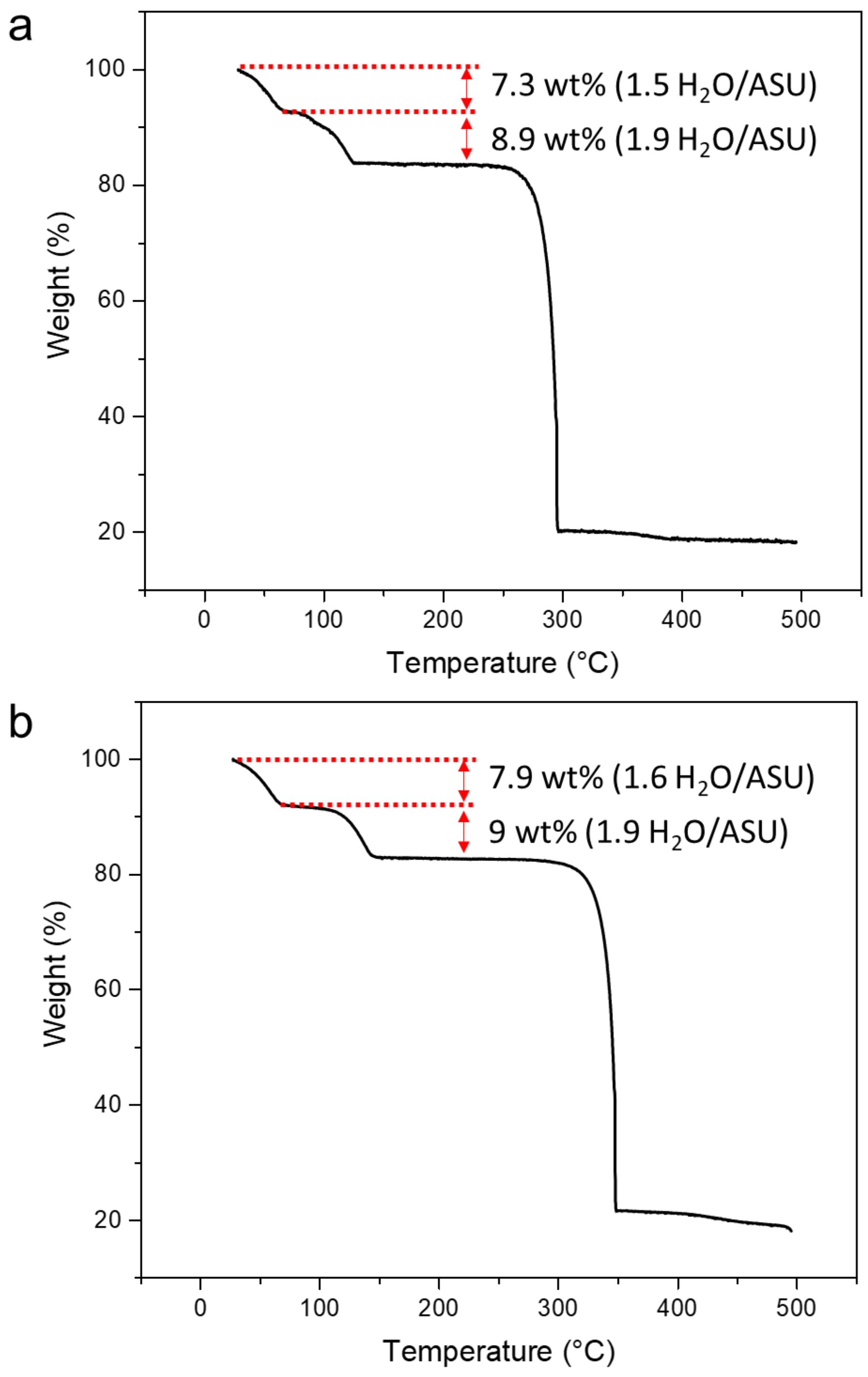

Figure S5 Thermogravimetric analysis measured by heating (a) **L-chn-1-Co-NO3** and (b) **L-chn-1-Ni-NO3** from room temperature to 500 °C at 10 °C min^{-1}. Two distinct mass losses, associated with the desorption of channel water and removal of coordinated water were observed.

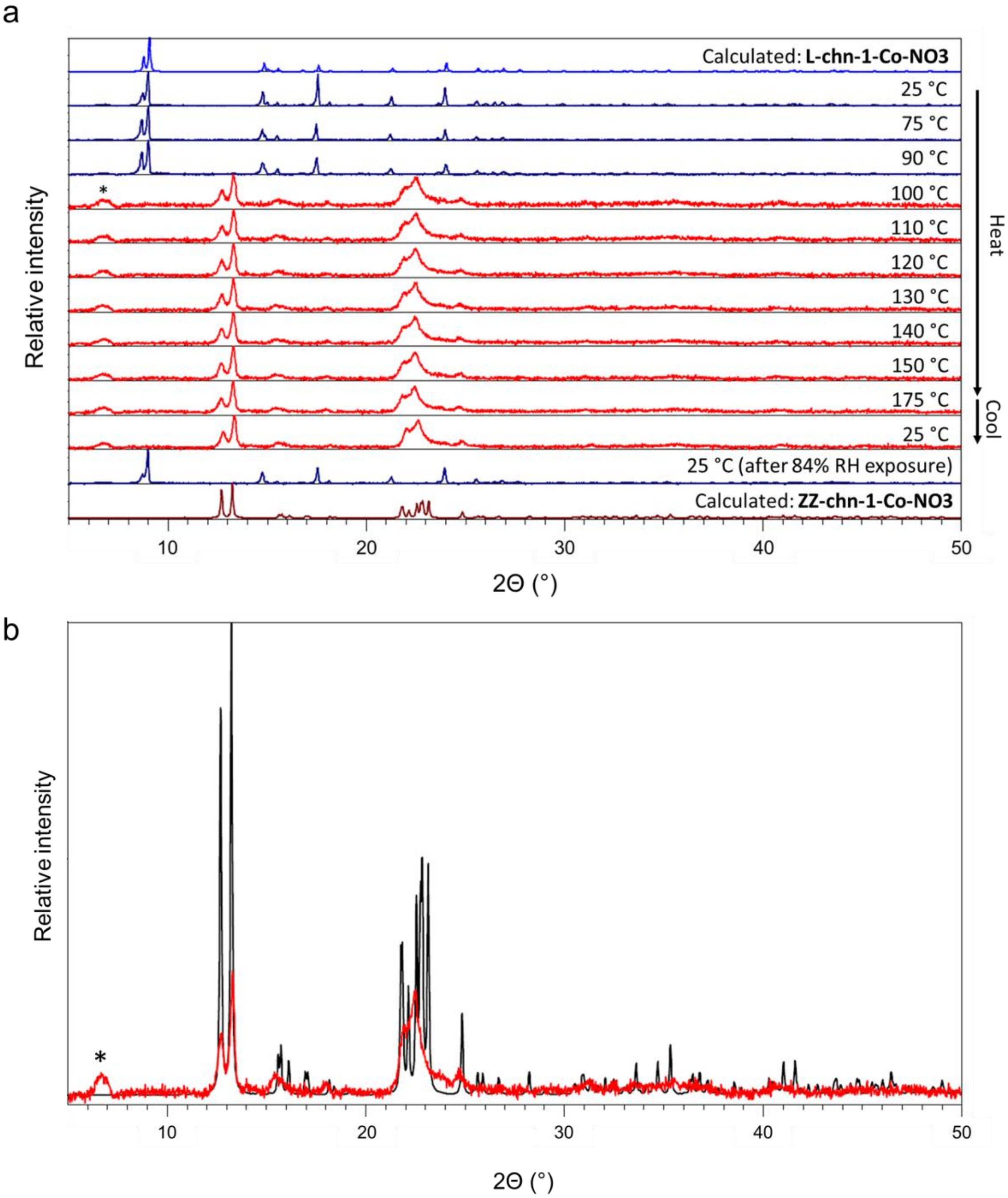

Figure S6 (a) Variable-temperature powder X-ray diffractograms of the conversion of **L-chn-1-Co-NO3** (blue) to **ZZ-chn-1-Co-NO3** (red) with experimental sequence progressing from top to bottom. Asterisk indicates additional peak contribution from zero-background holder (Figure S9). (b) Overlay of PXRD patterns of experimental **ZZ-chn-1-Co-NO3** (130 °C) and **ZZ-chn-1-Zn-NO3** calculated from a crystal structure retrieved from the CSD[10] using Mercury.[12] Refcode: DUFWIN.

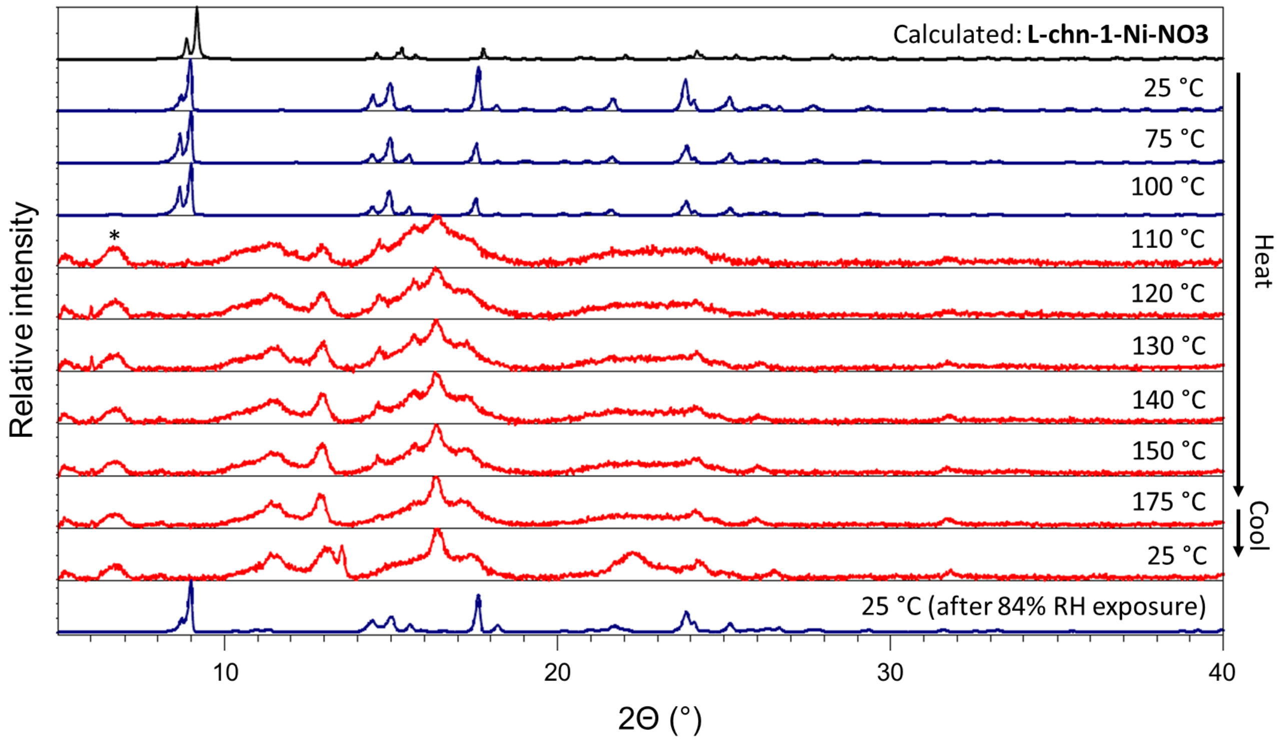

Figure S7 Reversable structural conversion of **L-chn-1-Ni-NO3** to **HT-Ni** during heating. Variable-temperature powder X-ray diffractograms of the conversion of **L-chn-1-Ni-NO3** (blue) to **HT-Ni** (red) with experimental sequence progressing from top to bottom. Asterisk indicates additional peak contribution from zero-background holder (Figure S9). Reference diffractogram was determined from a crystal structure retrieved from the CSD (Refcodes: FOGGUF).

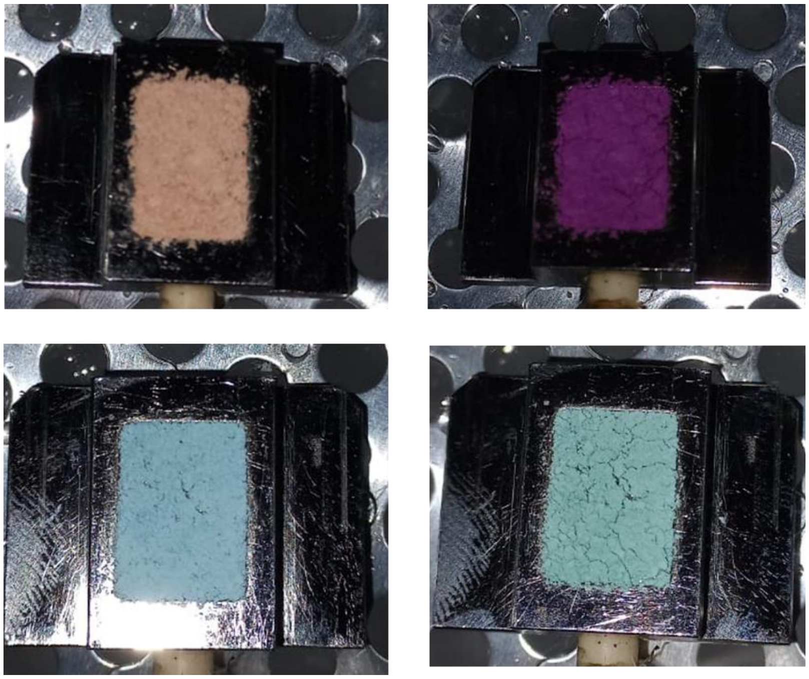

Figure S8 Powdered samples of **L-chn-1-Co-NO3** (top) and **L-chn-1-Ni-NO3** (bottom) before (left) and after (right) heating from RT to 175 °C.

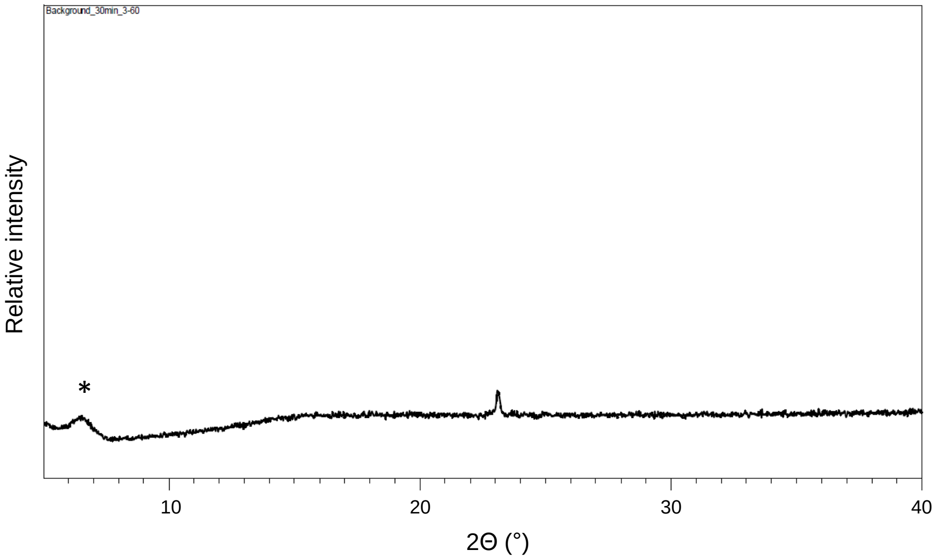

Figure S9 Powder X-ray diffractogram on PANalytical X'Pert Pro-MPD diffractometer with Anton Paar TTK 450 chamber without sample after cleaning with pure EtOH.

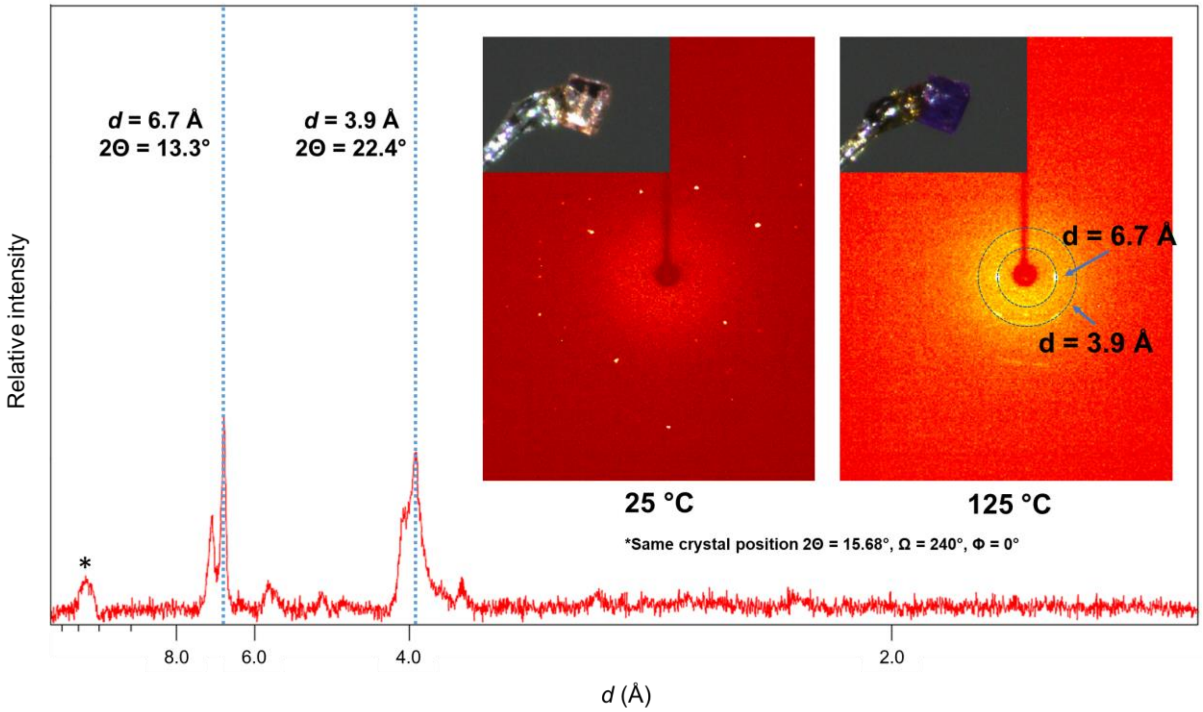

Figure S10 Powder X-ray diffractogram of **ZZ-chn-1-Co-NO3** obtained by integrating Debye rings in 2D image after heating **L-chn-1-Co-NO3** to 130 °C. Inset: Photomicrograph of a single crystal of **L-chn-1-Co-NO3** overlayed on a diffraction frame from SCXRD analysis at (left) 25 °C and (right) 125 °C. The reduction in diffraction quality indicates that the structural conversion to **ZZ-chn-1-Co-NO3** has occurred after heating. The *d*-spacing values of the largest Bragg peaks in the diffraction frame match those from high temperature PXRD. Asterisk indicates a peak caused by the zero-background holder (Figure S9).

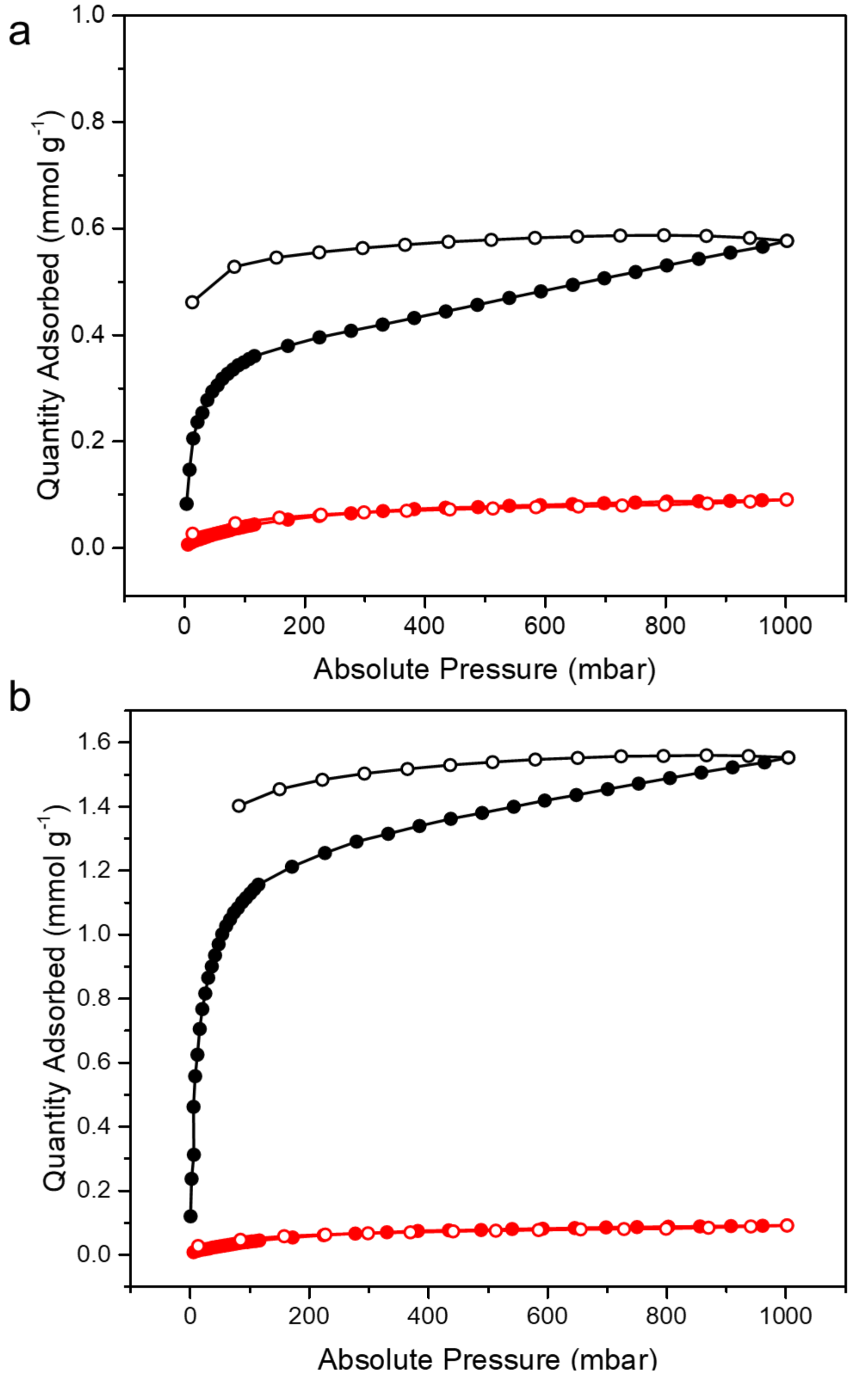

Figure S11 (a) Equilibrium CO_2 adsorption (closed circles) and desorption (open circles) isotherms of **L-chn-1-Co-NO3** (black) and **ZZ-chn-1-Co-NO3** (red) carried out at –78 °C. b) Equilibrium CO_2 adsorption (closed circles) and desorption (open circles) isotherms of **L-chn-1-Ni-NO3** (black) and **HT-Ni** (red) carried out at –78 °C . Degassing was done under dynamic vacuum (10^{-6} mmHg) at 25 °C and 110 °C for porous (black) and nonporous phases (red), respectively.

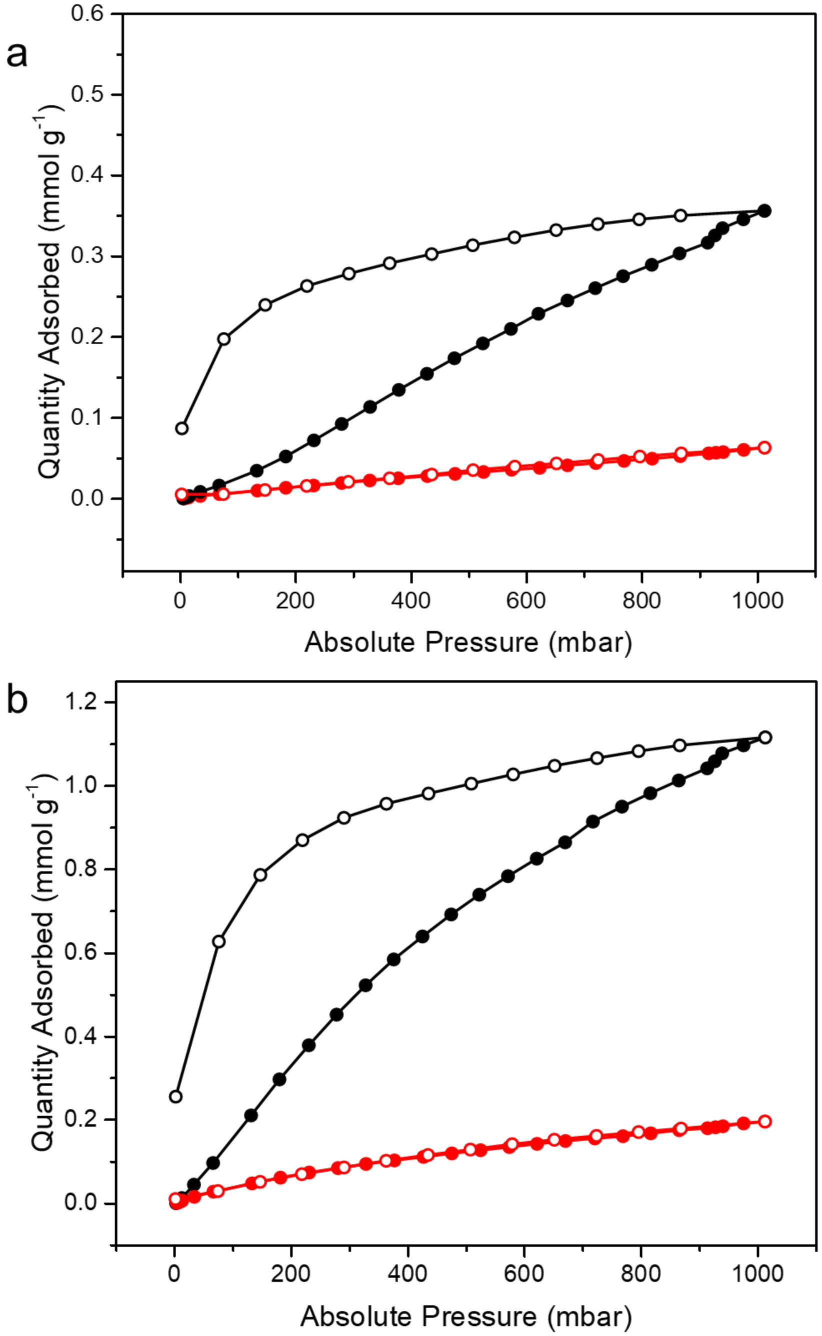

Figure S12 (a) Equilibrium CO_2 adsorption (closed circles) and desorption (open circles) isotherms of **L-chn-1-Co-NO3** (black) and **ZZ-chn-1-Co-NO3** (red) carried out at 25 °C. b) Equilibrium CO_2 adsorption (closed circles) and desorption (open circles) isotherms of **L-chn-1-Ni-NO3** (black) and **HT-Ni** (red) carried out at 25 °C . Degassing was done under dynamic vacuum (10^{-6} mmHg) at 25 °C and 110 °C for porous (black) and nonporous phases (red), respectively.

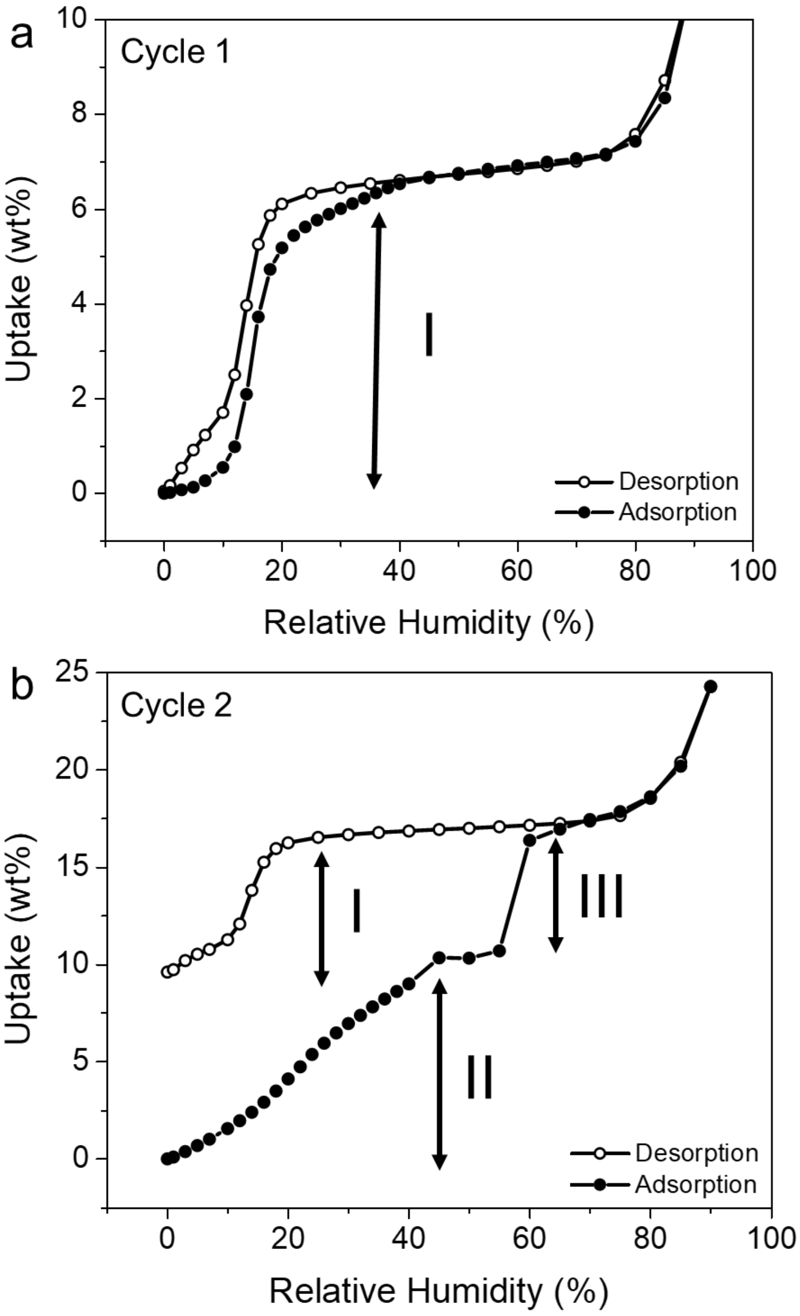

Figure S13 Equilibrium water adsorption (closed circles) and desorption (open circles) isotherms of (a) **L-chn-1-Ni-NO3** and (b) **Ni-HT** measured on a DVS instrument at 25 °C. The features of the sorption profiles are indicated with Roman numerals: I, pore-filling and pore emptying; II, recoordination of aqua ligands; III, pore-filling that occurs after recoordination of aqua ligands. Note that the upper limit displayed for isotherm in (b) has been truncated for comparison with (a) and that this sharp uptake results from condensation between particles.

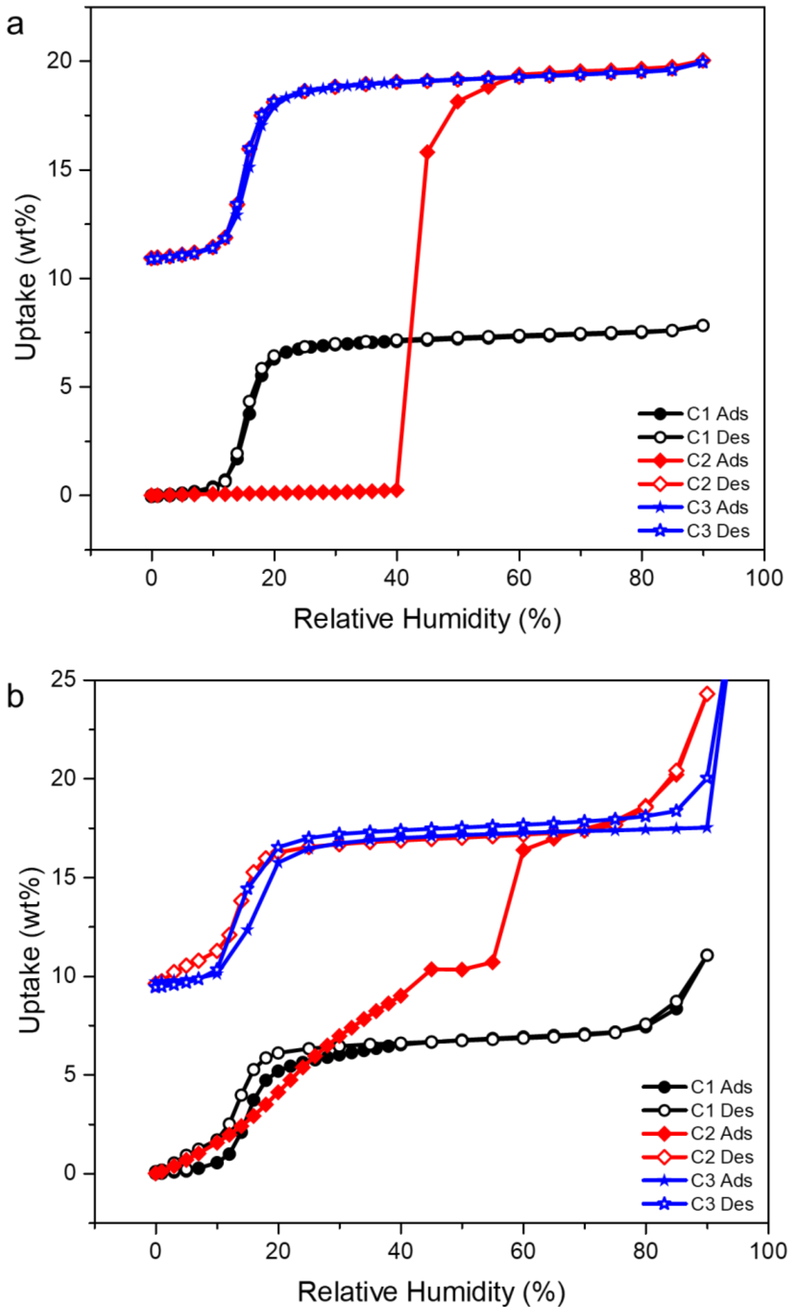

Figure S14 Equilibrium water vapor adsorption (closed symbols) and desorption (open symbols) isotherms measured on a DVS Adventure instrument at 25 °C. Three consecutive sorption cycles (C1, C2 and C3) were carried out starting with (a) **L-chn-1-Co-NO3** and (b) **L-chn-1-Ni-NO3** that was activated at (a) 25 °C (C1, black circles), (b) 150 °C (C2, red diamonds) and (c) 25 °C (C3, blue stars). Note that the upper limit displayed for isotherm in (b) has been truncated for comparison with (a) and that this sharp uptake results from condensation between particles.

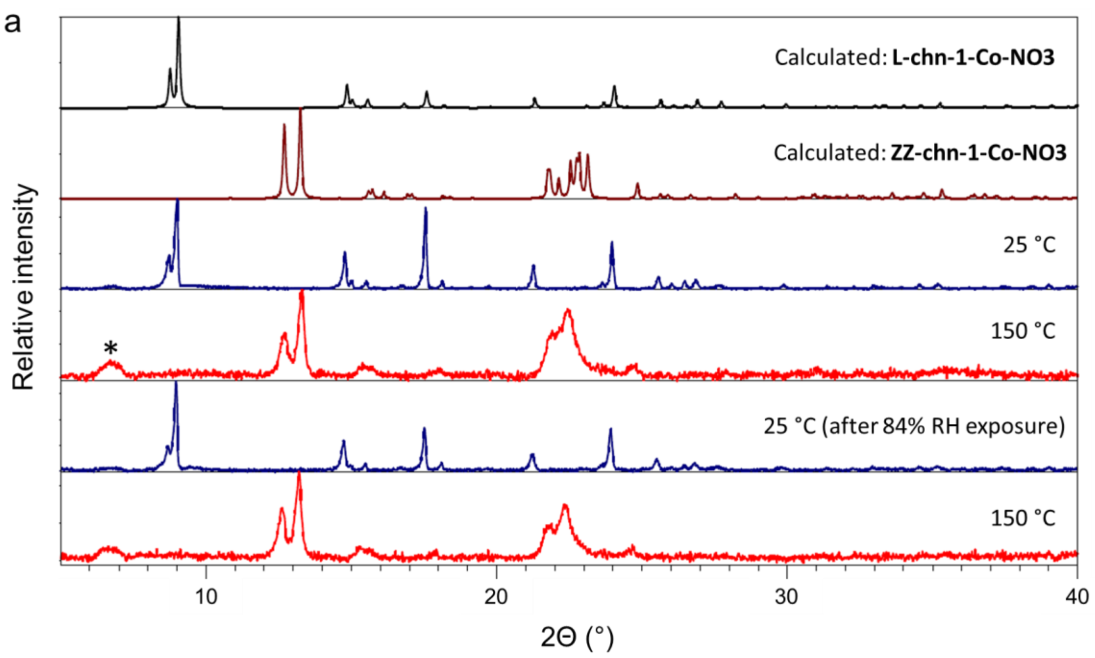

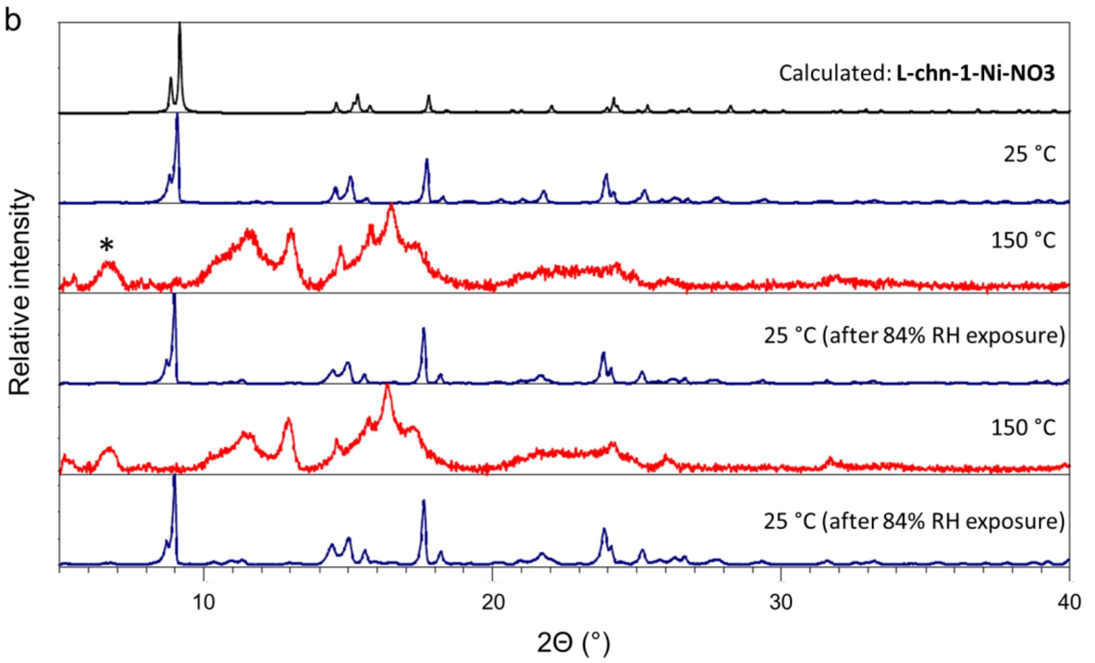

Figure S15 Variable-temperature powder X-ray diffractograms demonstrating the repeatable removal of aqua ligands from (a) **L-chn-1-Co-NO3** and (b) **L-chn-1-Ni-NO3** when heated to 150 °C, followed by regeneration after exposure to 84% RH at 25 °C. Asterisk indicates additional peak contribution from zero-background holder (Figure S9). Reference diffractograms were determined from crystal structures retrieved from the CSD[10] using Mercury.[12] Refcodes: FOGGIT, DUFWIN, FOGGUF.

3 Estimation of Adsorption Enthalpy

3.1 Estimation of Adsorption Enthalpy using Clausius-Clapeyron Approach

The enthalpy of adsorption Δh_{ads} (per mole of host) with respect to H_2O loading (wt%) was determined using the Clausius-Clapeyron relation

$$lnP = \frac{\Delta h_{ads}}{R} \cdot \frac{1}{T} + C$$

where the P is the partial pressure (Pa) of water at temperature T (K), R is the gas constant (8.314 kJ mol^{-1} K^{-1}), and C is a constant.[32] The values of Δh_{ads} were obtained from the isosteres of adsorption (Figure S19) derived from the first cycle of the three sorption isotherms at 15, 27 and 40 °C (Figures S16-S18). It should be noted that the Clausius-Clapeyron relation might give inaccurate results at low vapor pressures,[33] hence, we chose the minimum partial pressure considered for these calculations to such that they correspond to 0.1% RH (0.7 wt%). The value of Δh_{ads} at zero coverage was estimated from these low coverage data to be Δh_{ads} = −45.0 ± 0.1 kJ mol^{-1} and −45.4 ± 0.1 kJ mol^{-1} for **L-chn-1-Co-NO3** and **L-chn-1-Ni-NO3**, respectively (Figure S20).

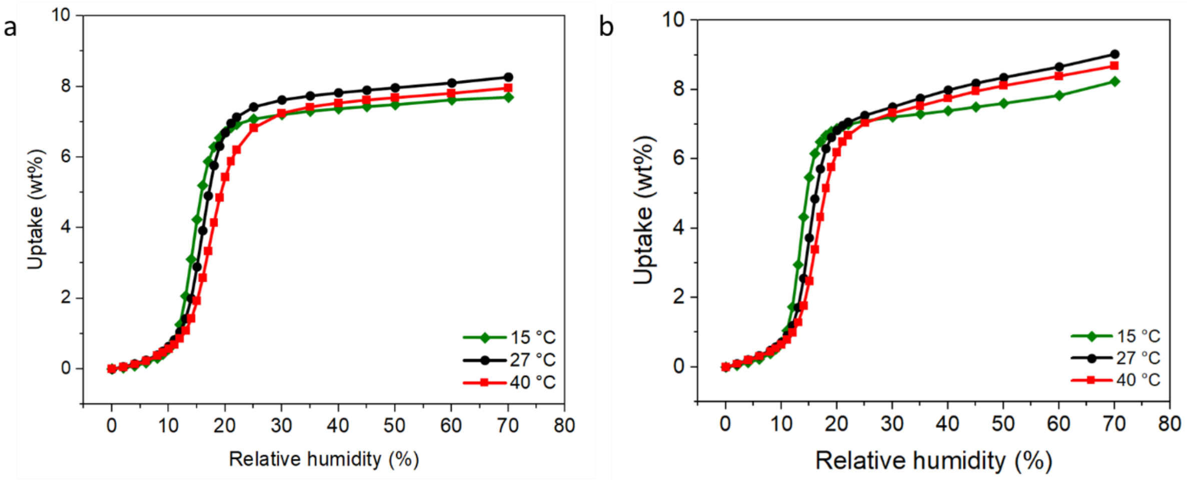

Figure S16 Water vapor adsorption isotherms of a) **L-chn-1-Co-NO3** and b) **L-chn-1-Ni-NO3** measured at 15 °C (green diamonds), 27 °C (black circles) and 40 °C (red squares) as a function of relative humidity (%).

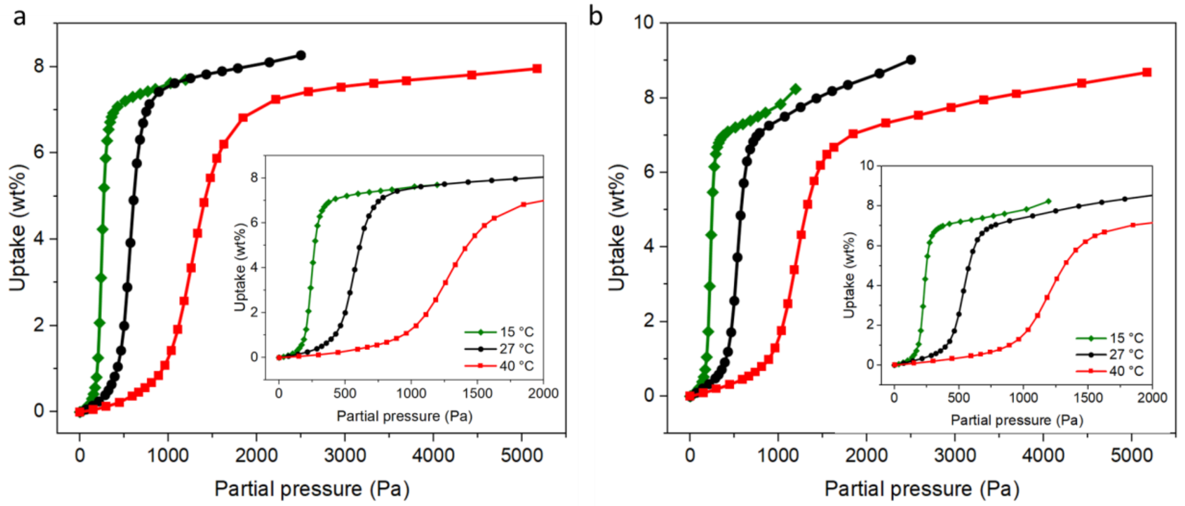

Figure S17 Water vapor adsorption isotherms of a) **L-chn-1-Co-NO3** and b) **L-chn-1-Ni-NO3** measured at 15 °C (green diamonds), 27 °C (black circles) and 40 °C (red squares) as a function of H_2O partial pressure (Pa).

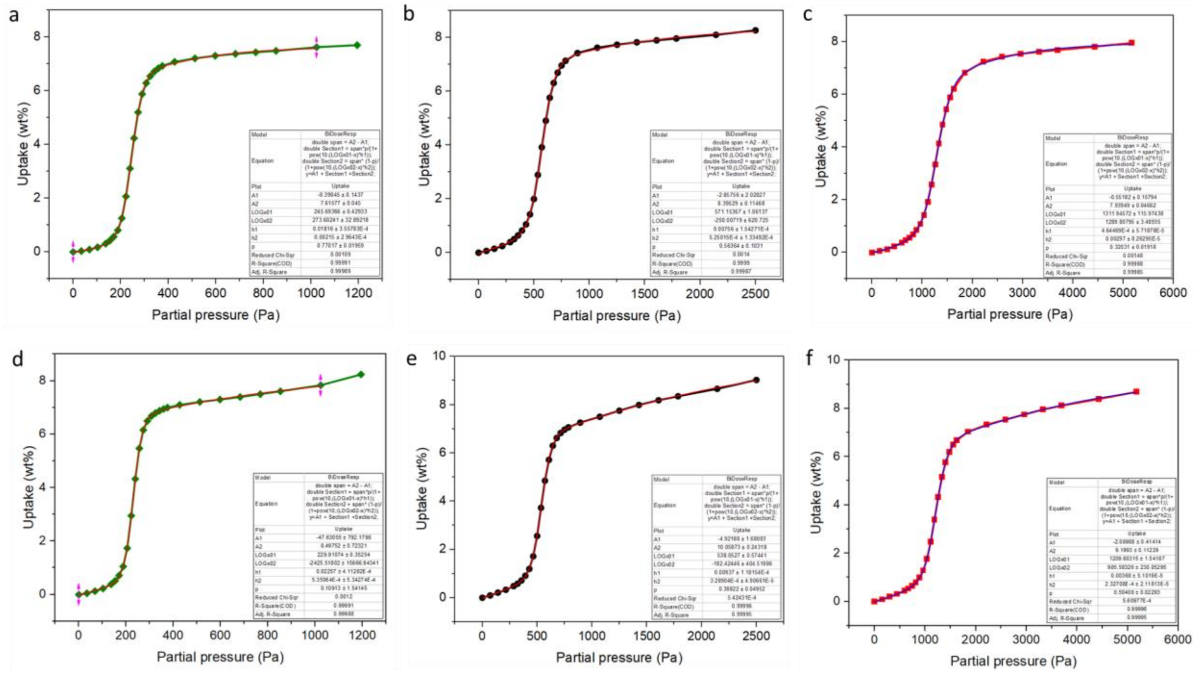

Figure S18 Water vapor adsorption isotherms used for calculating Δh_{ads} of a-c) **L-chn-1-Co-NO3** and d-f) **L-chn-1-Ni-NO3** recorded at 10 °C (left, green diamonds), 27 °C (middle, black circles) and 40 °C (right, red squares) using a dynamic vapor sorption analyser. Details regarding the fitting function to determine uptake as a function of partial pressure are shown as insets. The function is overlaid as a red line for 10 °C and 27 °C and a black line for 40 °C.

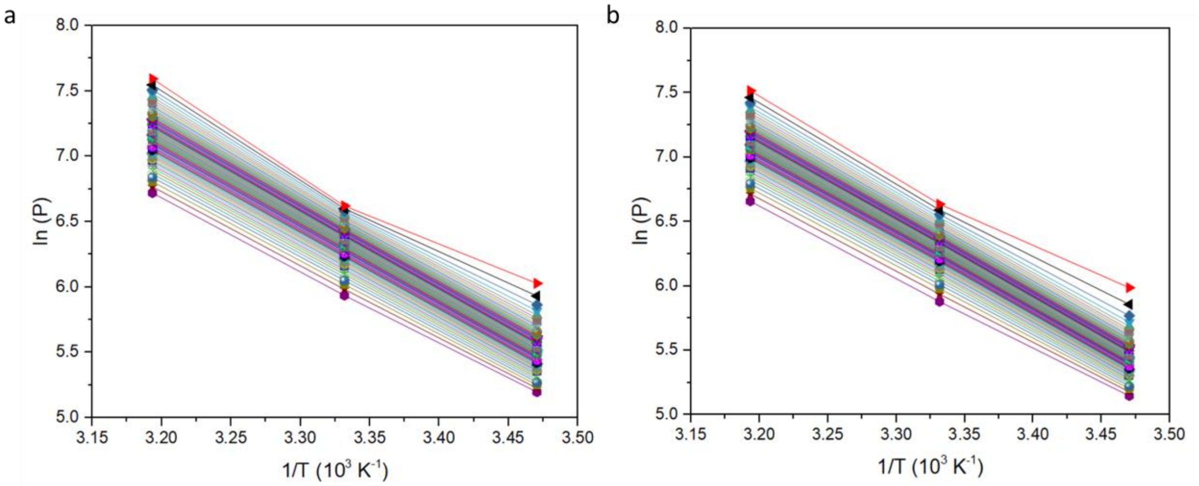

Figure S19 Adsorption isosteres used to calculate Δh_{ads} of a) **L-chn-1-Co-NO3** and b) **L-chn-1-Ni-NO3**.

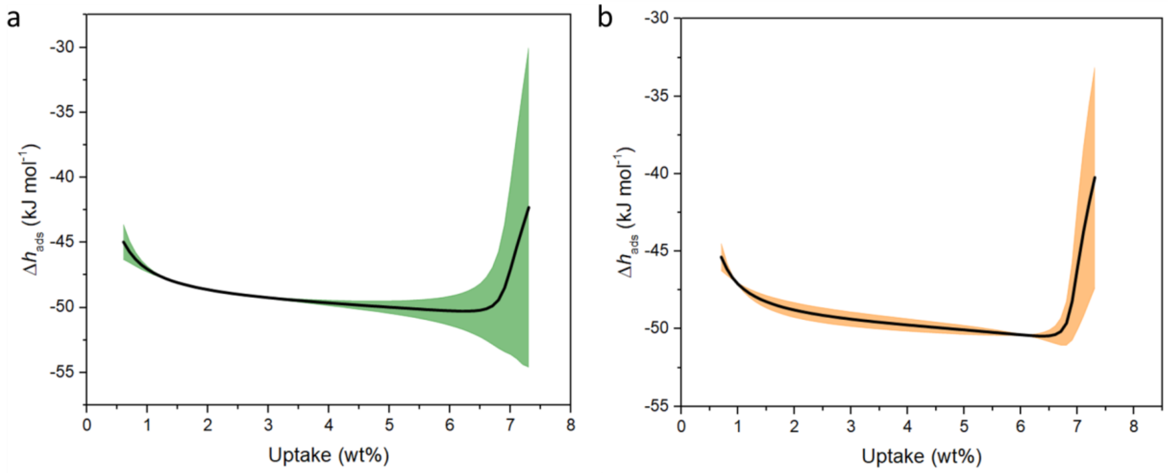

Figure S20 Enthalpy of adsorption Δh_{ads} with respect to H_2O uptake in a) **L-chn-1-Co-NO3** and b) **L-chn-1-Ni-NO3**. The standard error is shown as shaded regions.

3.2 Estimation of Adsorption Enthalpy using Differential Scanning Calorimetry

The enthalpies associated with the pore emptying mechanism (I, Figure 2, Figure S13) and loss of aqua ligands and concomitant structural rearrangements (II, Figure 2, Figure S13) were determined using temperature-ramp DSC analysis. It should be noted that these measurements allow for the approximation of the adsorption enthalpies.[34]

DSC thermograms were recorded using a TA instruments Q2000 analyzer equipped with a Refrigerated Cooling System (RCS90). During experiments, the sample compartment was purged using nitrogen gas (50 cm^3 min^{-1}). Samples of the hydrous porous phases (i.e. **L-chn-1-Co-NO3** and **L-chn-1-Ni-NO3**) were prepared by keeping them at ambient conditions (43% RH, 21 °C) for 4 h. Each sample (powdered, 8.5 mg) underwent two heating and cooling cycles using the following sequence: the sample was cooled from RT to 2 °C and kept isothermal for 3 min, followed by heating to 120 °C at a ramp rate of 1 °C min^{-1} and finally cooled to 2 °C at 1 °C min^{-1}. This temperature range was chosen to ensure that sufficient baseline could be achieved before the onset and after the completion of dehydration. In all cases, the sample was placed in an open aluminum pan to allow for the loss of water vapor during heating.

Two peaks (exothermic) corresponding to pore emptying of the porous phases $\Delta h_{des,I}$ (I, Figure 2, Figure S13) and loss of aqua ligands and structural rearrangement $\Delta h_{des,II}$ (II, Figure 2, Figure S13) were observed for both analogues the thermogram of the first cycle (Figure S21). The values of $\Delta h_{des,I}$ = -51.8 and -48.0 kJ mol^{-1}, for **L-chn-1-Co-NO3** and **L-chn-1-Ni-NO3**, respectively. These are in agreement with the Δh_{ads} values associated with pore filling determined from DVS analysis (Figure S20): Δh_{ads} values at the half uptake points are -49.6 ± 0.2 kJ mol^{-1} (3.8 wt%) and -49.6 ± 0.4 kJ mol^{-1} (3.6 wt%) for **L-chn-1-Co-NO3** and **L-chn-1-Ni-NO3**, respectively. The values of $\Delta h_{des,II}$ = -50.8 kJ mol^{-1} and -51.4 kJ mol^{-1} for transformations **L-chn-1-Co-NO3→ZZ-chn-1-Co-NO3** and **L-chn-1-Ni-NO3→HT-Ni**, respectively.

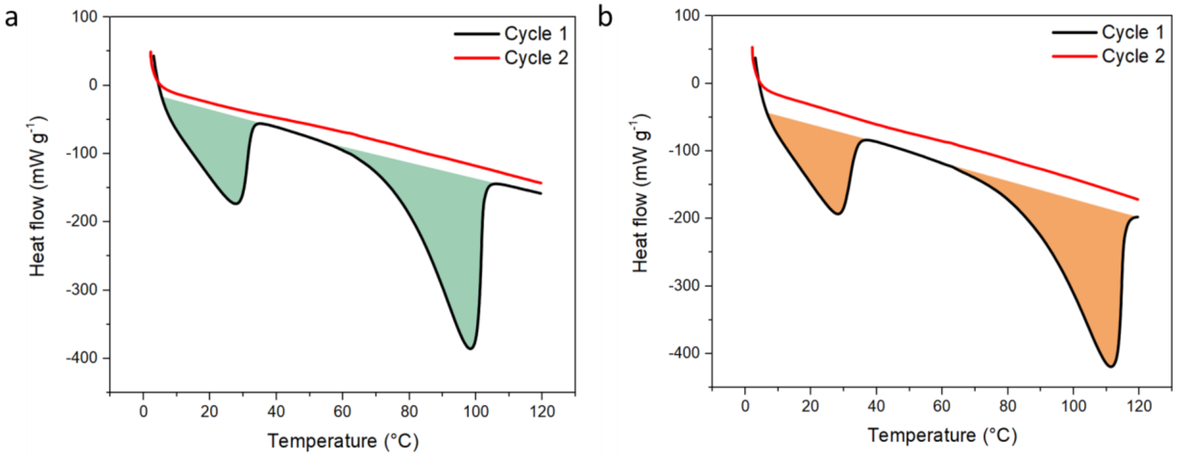

Figure S21 DSC thermograms of the heating segments for a) **L-chn-1-Co-NO3** and b) **L-chn-1-Ni-NO3** over two cycles (Cycle 1, black line; Cycle 2, red line). The values of $\Delta h_{des,I}$ and $\Delta h_{des,II}$ were determined by integration of the shaded regions (with respect to time).

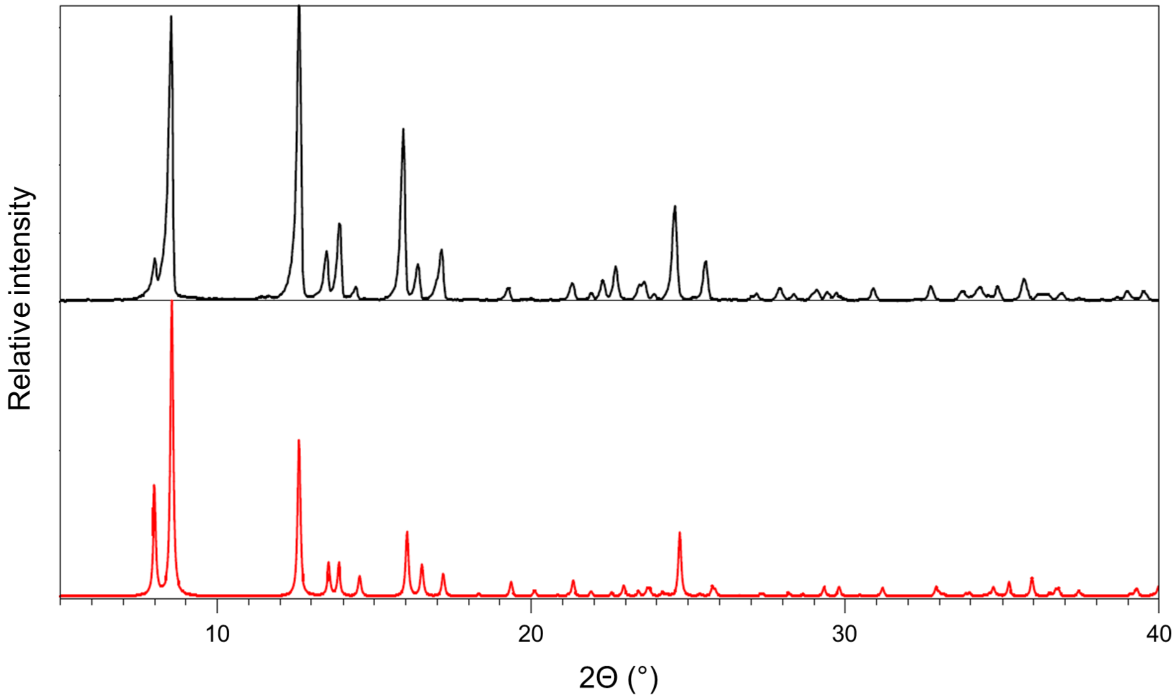

Figure S22 Powder X-ray diffractograms of **ROS-037** (black) and calculated (red) from a crystal structure retrieved from the CSD[10] using Mercury.[12] Refcode: MABJOP.

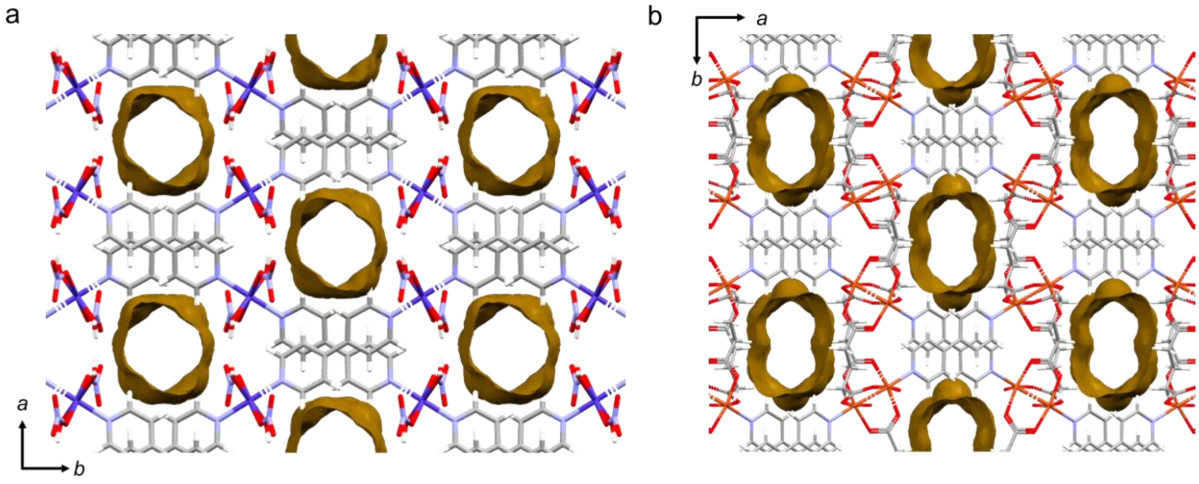

Figure S23 Projections along [001] for (a) **L-chn-1-Co-NO3** and (b) **ROS-037** modelled from SCXRD data measured at −173 °C and calculated from a crystal structure deposited in the CSD[10] (Refcode, MABJOP), respectively. Atom colors: gray, carbon; white, hydrogen; blue, nitrogen; red, oxygen; purple, cobalt; orange, Cu.

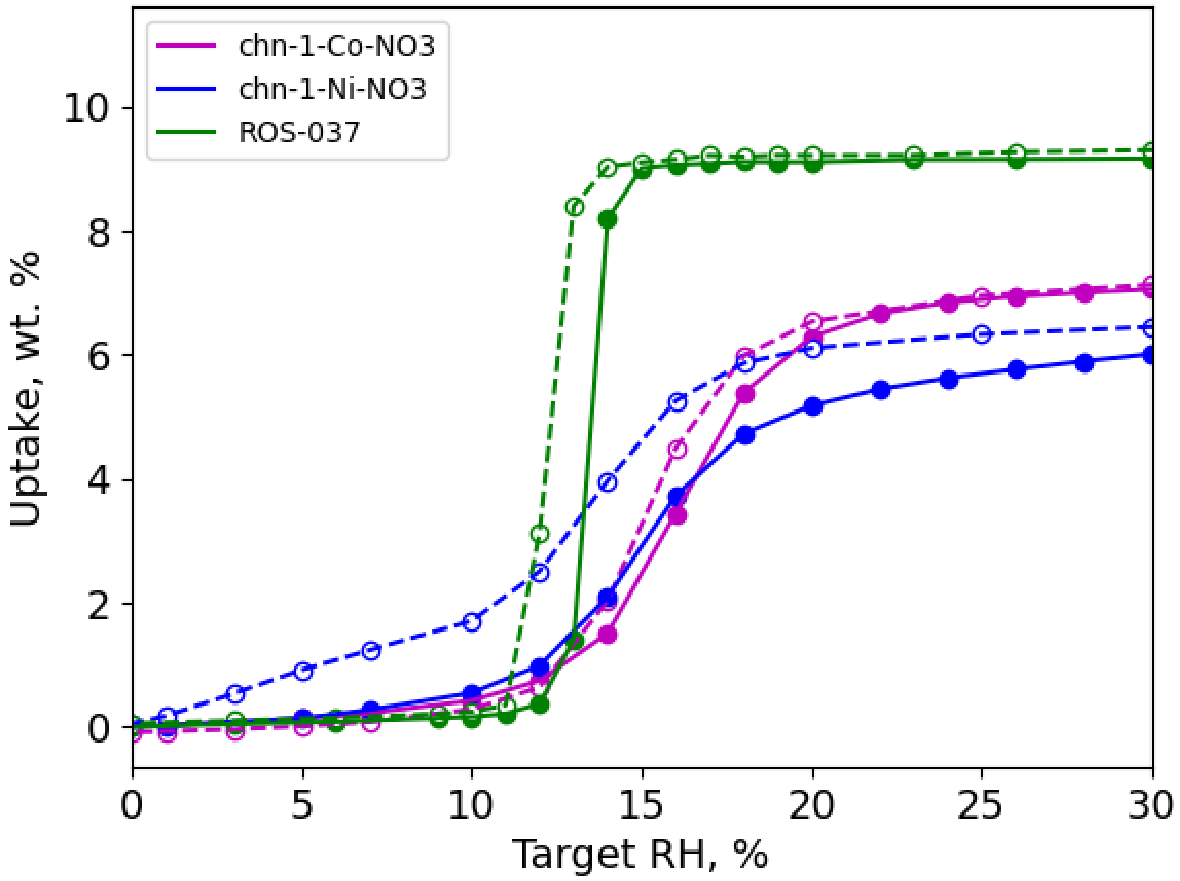

Figure S24 Equilibrium water vapor adsorption (closed circles) and desorption (open circles) isotherms of **L-chn-1-Co-NO3** (purple), **L-chn-1-Ni-NO3** (blue) with particle size 50–100 μm and **ROS-37** (green) isotherm reported in ref [2] measured on a DVS Adventure instrument at 25 °C.

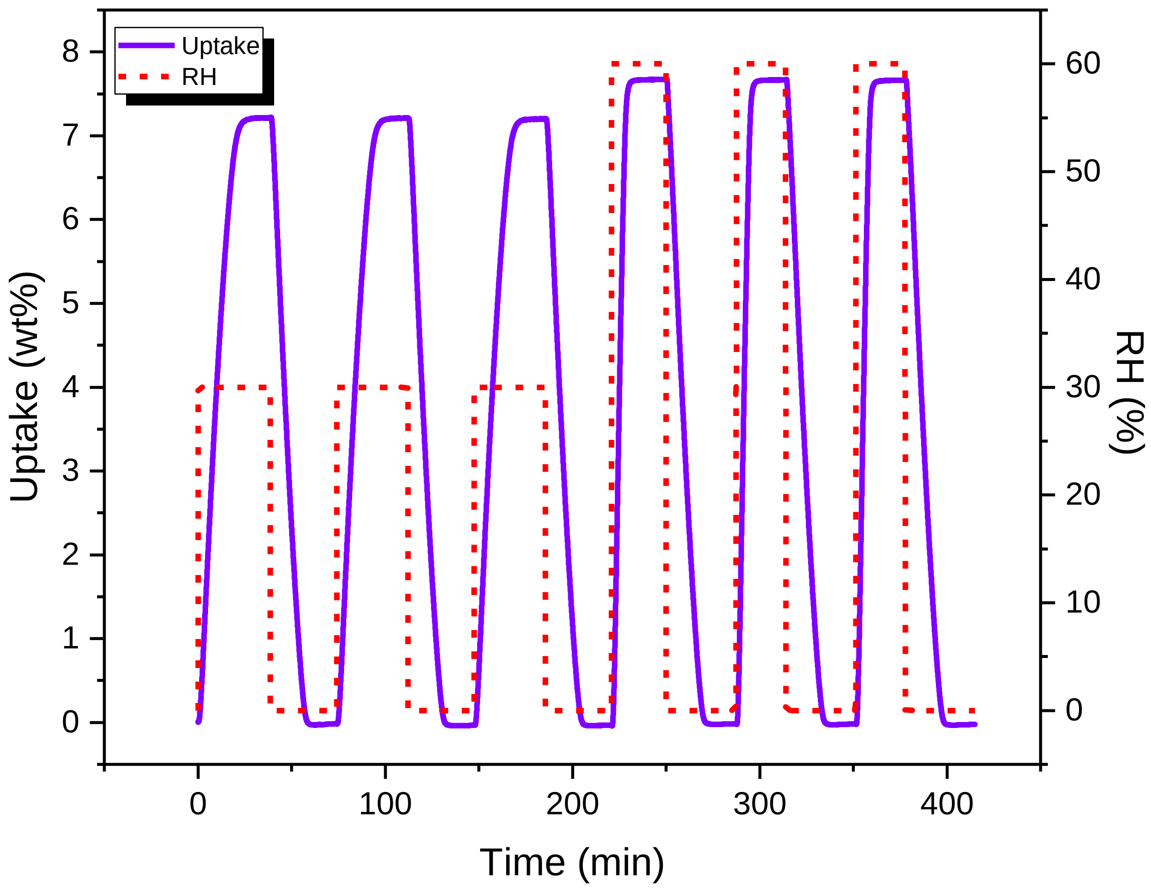

Figure S25 Six water vapor sorption cycles carried out on **L-chn-1-Co-NO3** using an Adventure DVS instrument at 25 °C: 3 consecutive 0–30% RH swing experiments (target RH of 40 min ads, 40 min des) followed by 3 consecutive 0–60% RH swing experiments (target RH of 30 min ads, 40 min des).

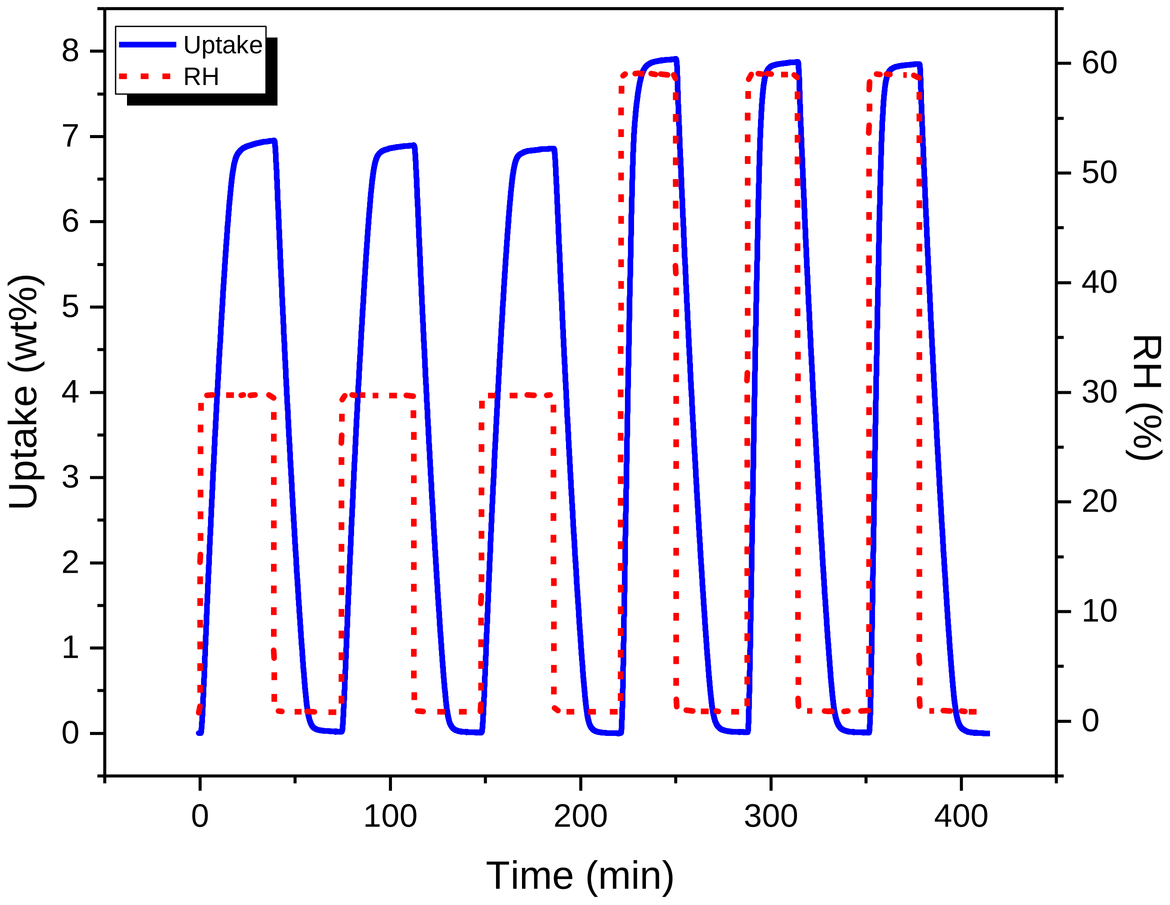

Figure S26 Six water vapor sorption cycles carried out on **L-chn-1-Ni-NO3** using an Adventure DVS instrument at 25 °C: 3 consecutive 0–30% RH swing experiments (target RH of 40 min ads, 40 min des) followed by 3 consecutive 0–60% RH swing experiments (target RH of 30 min ads, 40 min des).

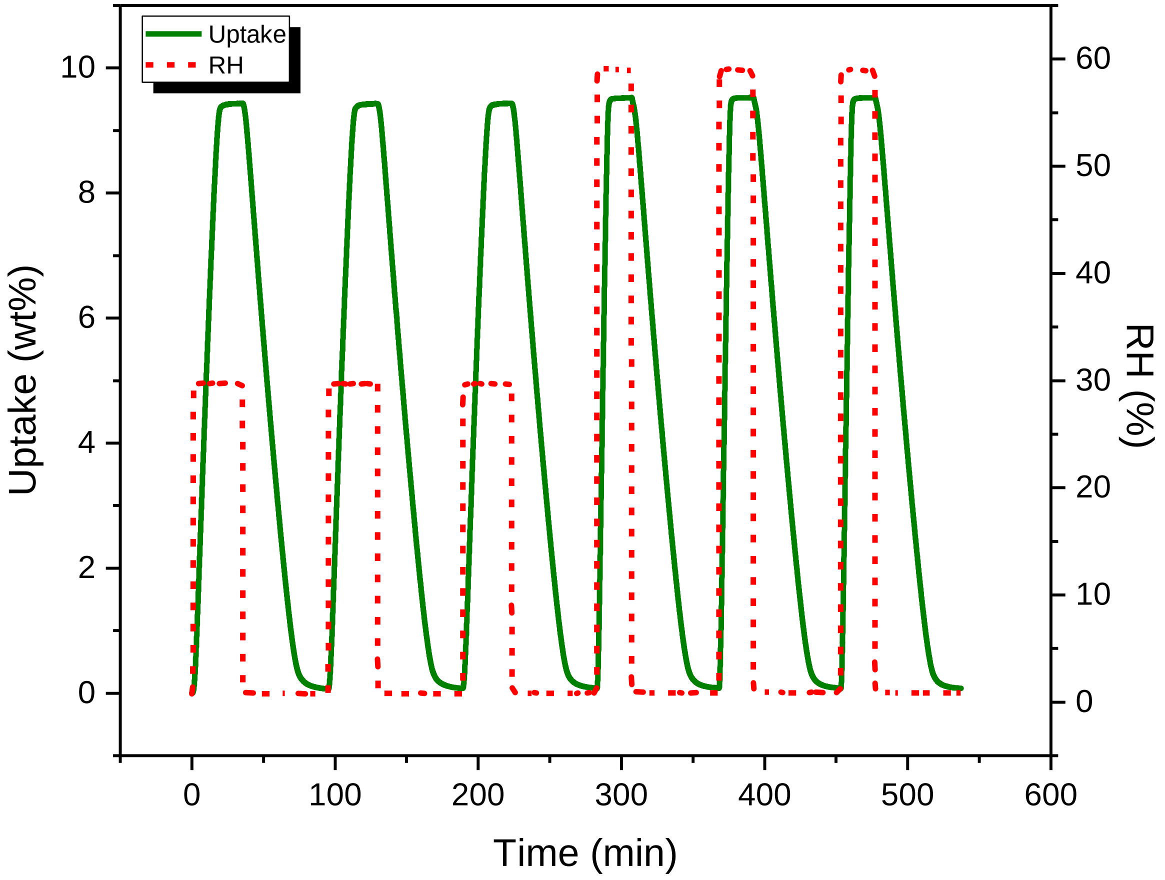

Figure S27 Six water vapor sorption cycles carried out on **ROS-37** using an Adventure DVS instrument at 25 °C: 3 consecutive 0–30% RH swing experiments (target RH of 30 min ads, 60 min des) followed by 3 consecutive 0–60% RH swing experiments (target RH of 20 min ads, 60 min des).

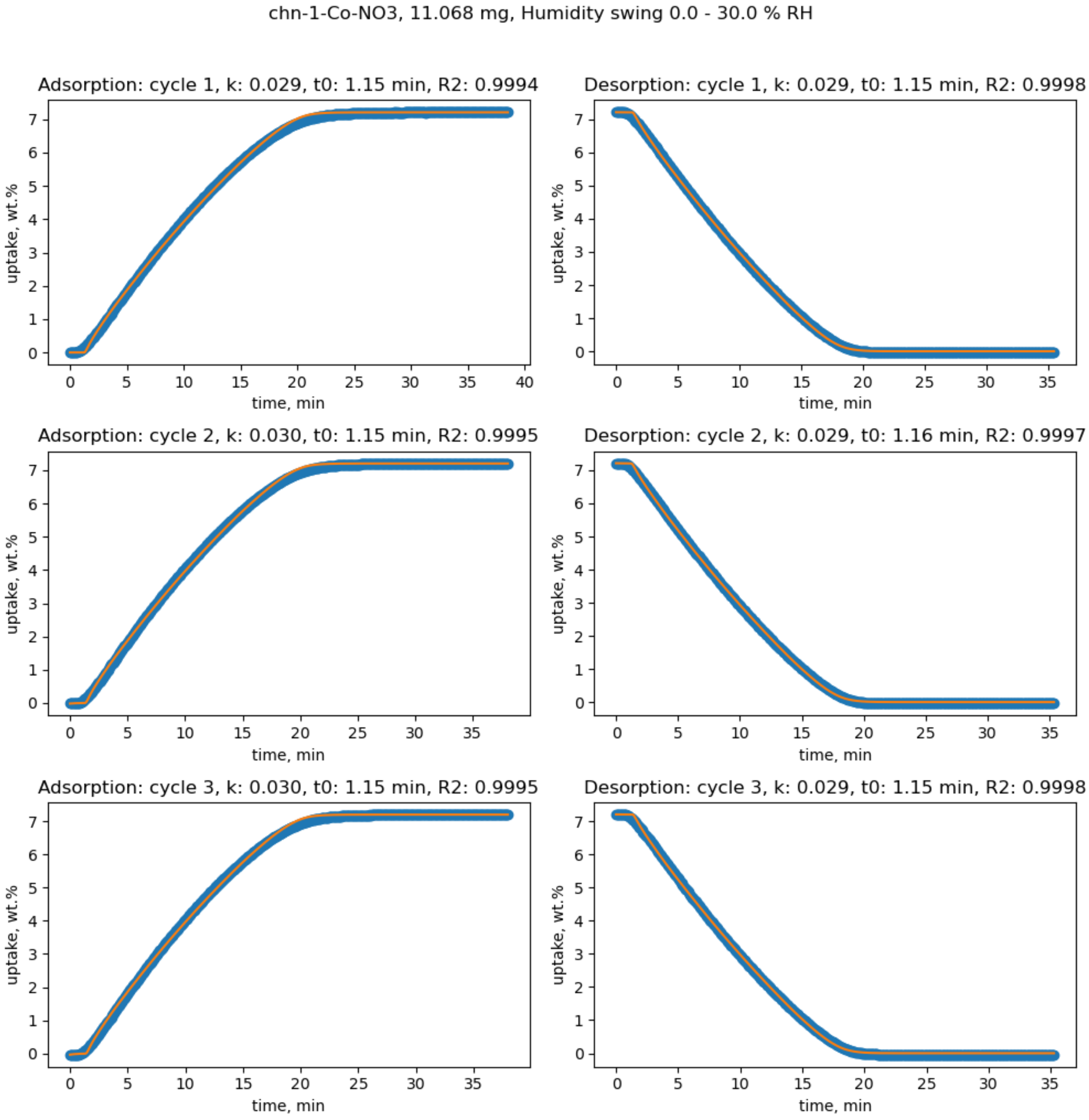

Figure S28 Fit of water vapor sorption kinetics data using isotherm-based model.[3] Adsorption (left) and desorption (right) kinetic curve fits during 3 RH-swing (0–30% RH) cycles for **L-chn-1-Co-NO3** with particle size 50–100 µm using an Adventure DVS instrument at 25 °C.

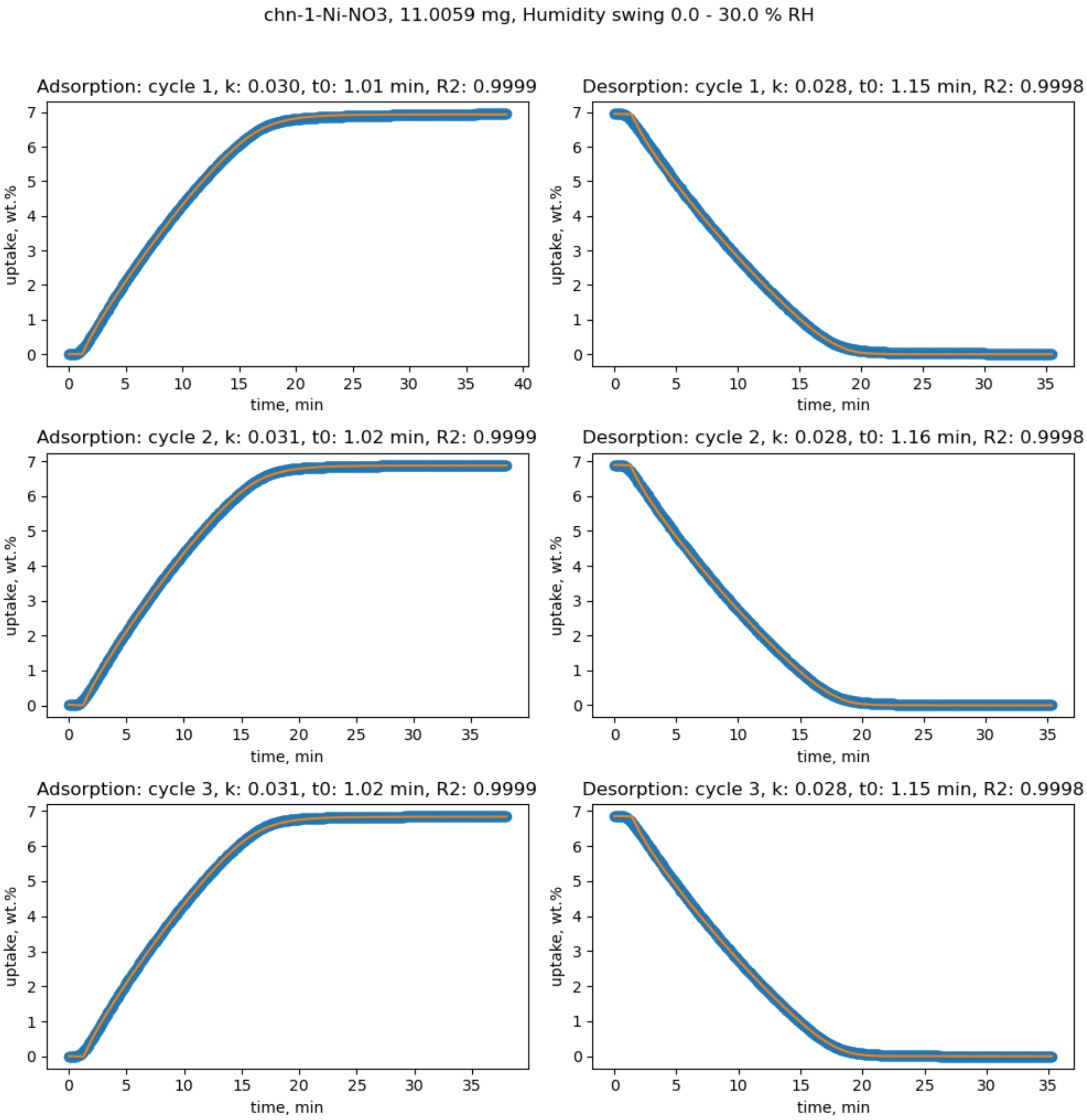

Figure S29 Fit of water vapor sorption kinetics data using isotherm-based model.[3] Adsorption (left) and desorption (right) kinetic curve fits during 3 RH-swing (0–30% RH) cycles for **L-chn-1-Ni-NO3** with particle size 50–100 μm using an Adventure DVS instrument at 25 °C.

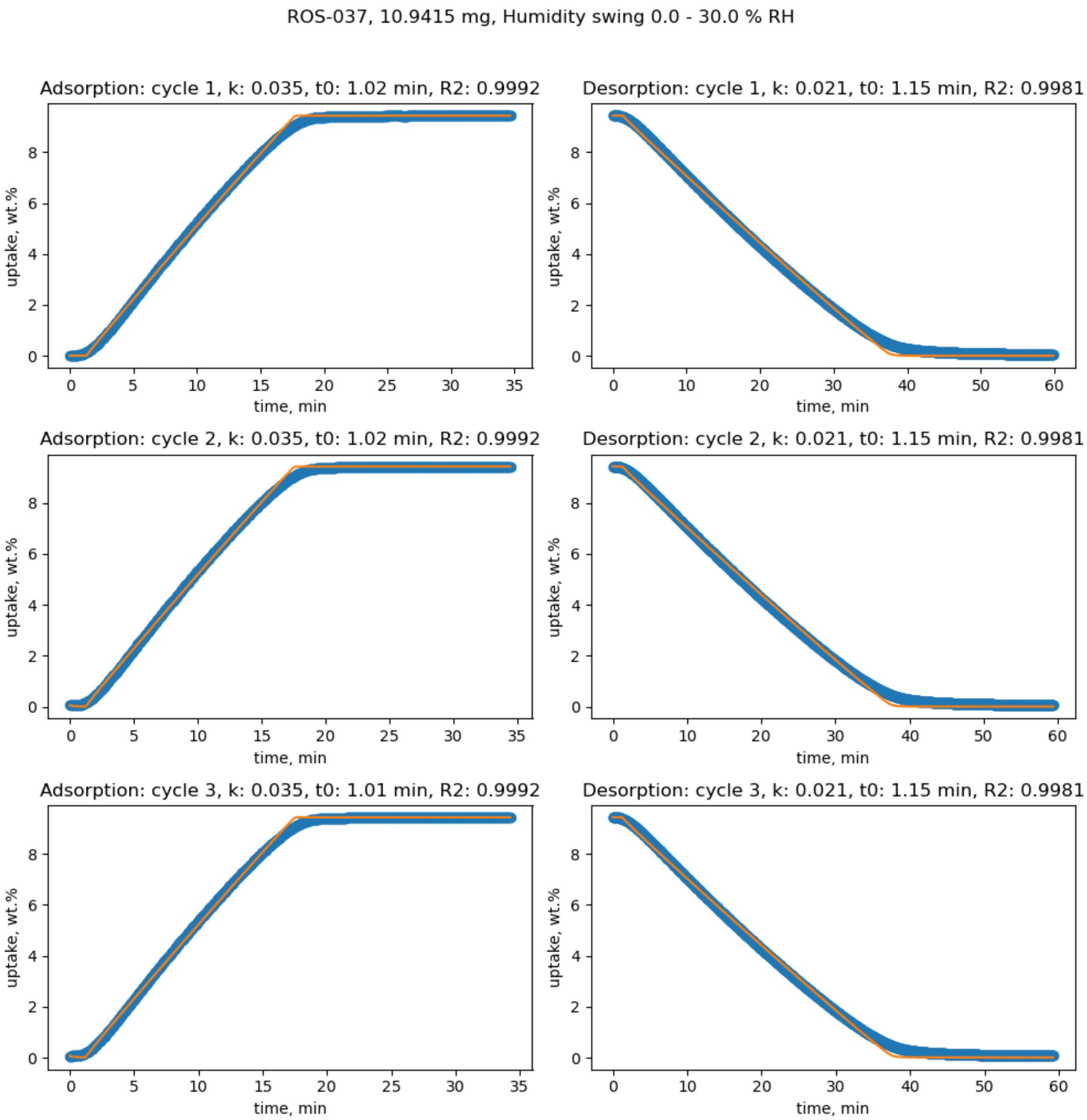

Figure S30 Fit of water vapor sorption kinetics data using isotherm-based model.[3] Adsorption (left) and desorption (right) kinetic curve fits during 3 RH-swing (0–30% RH) cycles for **ROS-037** with particle size 50–100 μm using an Adventure DVS instrument at 25 °C.

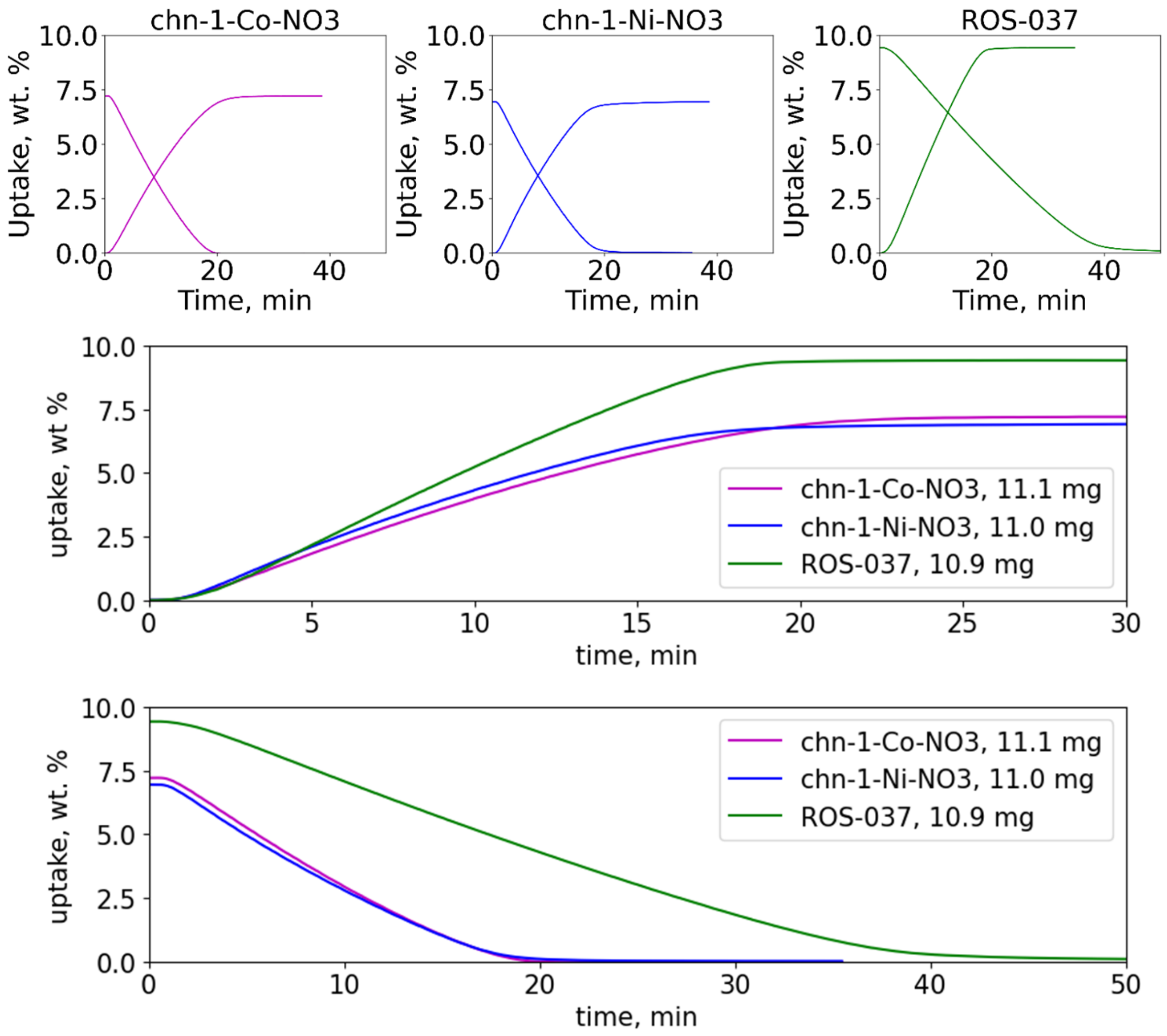

Figure S31 Water adsorption (0–30% RH) and desorption (30–0% RH) kinetic plots measured for **L-chn-1-Co-NO3** (purple), **L-chn-1-Ni-NO3** (blue) and **ROS-037** (green) with particle size 50–100 µm using a DVS Adventure instrument at 25 °C.

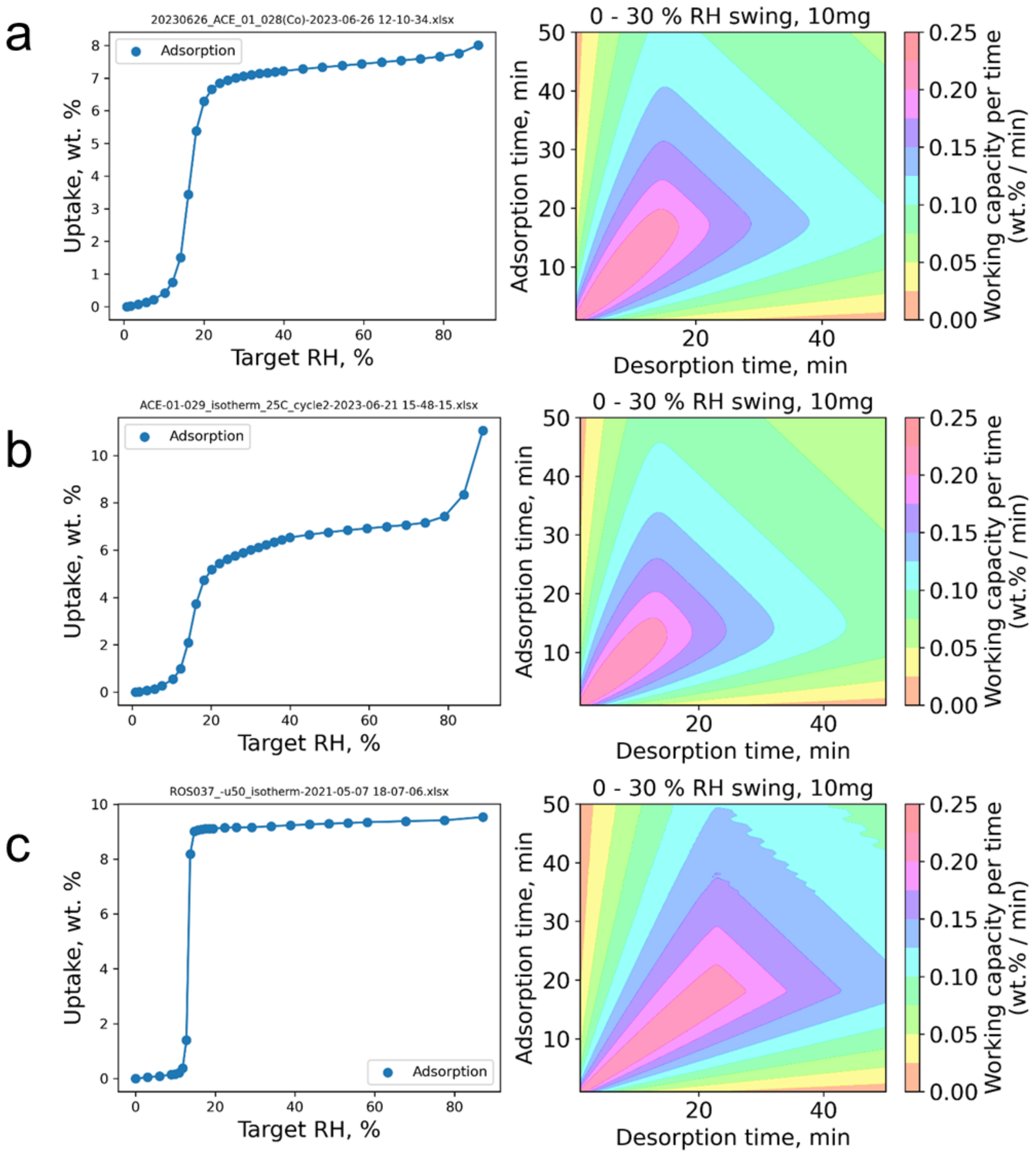

Figure S32 Water vapor sorption productivity 'heatmaps' (right) calculated from the adsorption isotherms (left) for (a), L-**chn-1-Co-NO3** ,(b) L-**chn-1-Ni-NO3** and (c) **ROS-037** measured at 25 °C.

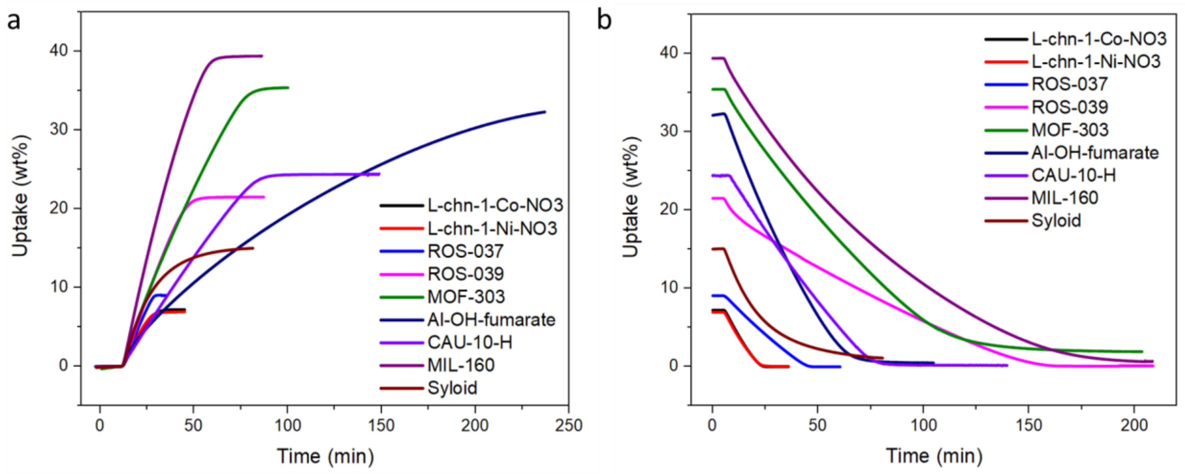

Figure S33 Water vapor a) adsorption and b) desorption humidity-swing kinetic curves ([30% RH, 300 K] – [0% RH, 300 K]) for **L-chn-1-Co-NO3**, **L-chn-1-Ni-NO3** and those reported in [1] showing relative uptake (wt%) as a function of time (min).

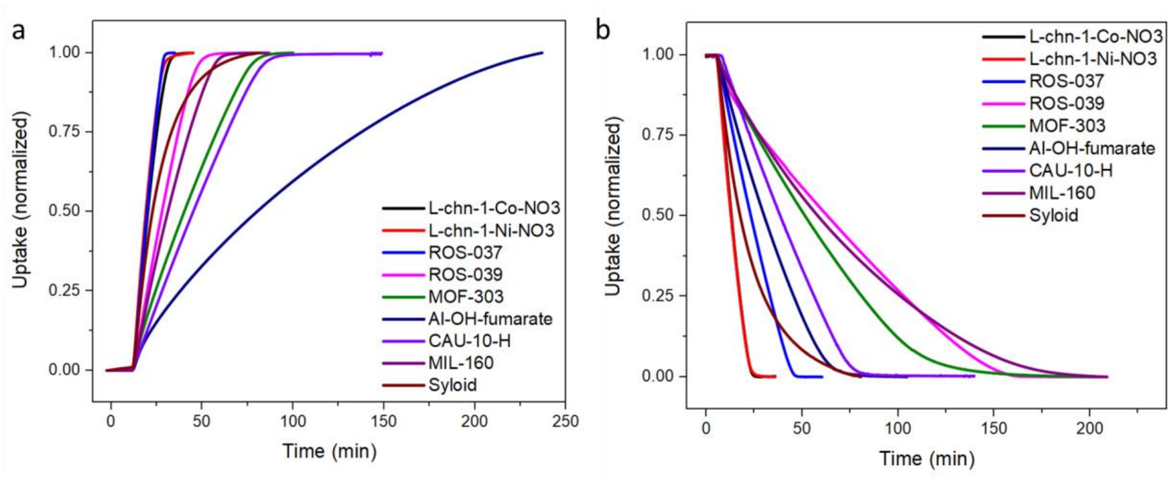

Figure S34 Water vapor a) adsorption and b) desorption humidity-swing kinetic curves ([30% RH, 300 K] – [0% RH, 300 K]) for **L-chn-1-Co-NO3**, **L-chn-1-Ni-NO3** and those reported in [1] showing normalized uptake (wt%) as a function of time (min).

Table S2 Total adsorption and desorption times (min) determined from kinetic humidity-swing measurements ([30% RH, 300 K] – [0% RH, 300 K]) on **L-chn-1-Co-NO3** and **L-chn-1-Ni-NO3** and from [2: Bezrukov *et al. Cell Rep.* **2023**, *4* (2), 101252]

Desiccant	Adsorption time (min)	Desorption time (min)	Reference
**L-chn-1-Co-NO3**	22	21	This work
**L-chn-1-Ni-NO3**	23	21	This work
**ROS-037**	19	43	This work
**ROS-039**	47	157	[2]
**MIL-160**	57	204	[2]
**Syloid**	69	76	[2]
**MOF-303**	82	154	[2]
**CAU-10-H**	88	82	[2]
**AL-OH-fumarate**	225	80	[2]

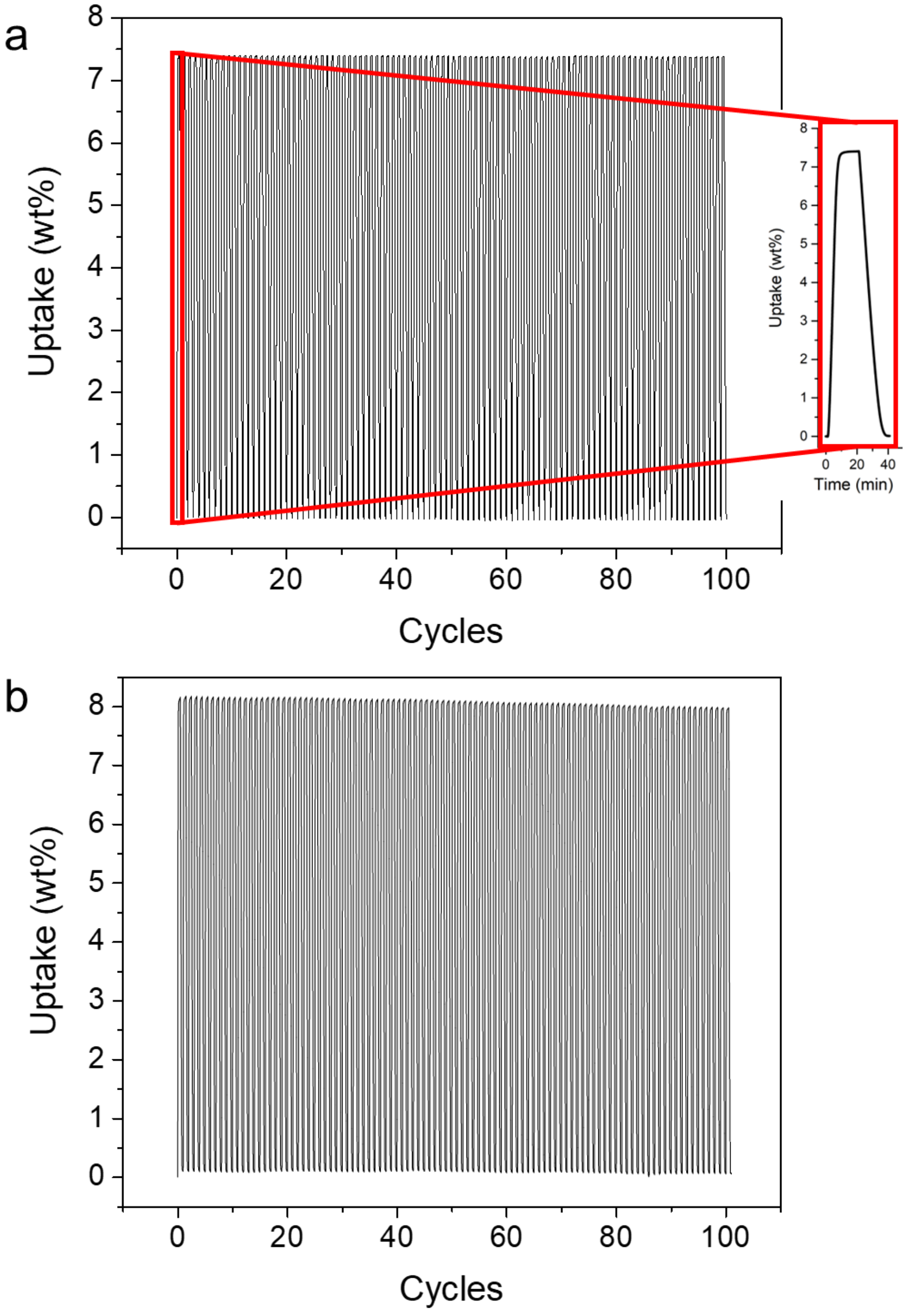

Figure S35 Water vapor sorption cycling experiments carried out on (a) **L-chn-1-Co-NO3** and (b) **L-chn-1-Ni-NO3** using a DVS Intrinsic instrument at 25 °C: 100 consecutive 0–60% RH swing experiments for **L-chn-1-Co-NO3** (target RH of 20 min ads, 20 min des) and **L-chn-1-Ni-NO3** (target RH of 30 min ads, 30 min des). Inset: first ad/desorption cycle.

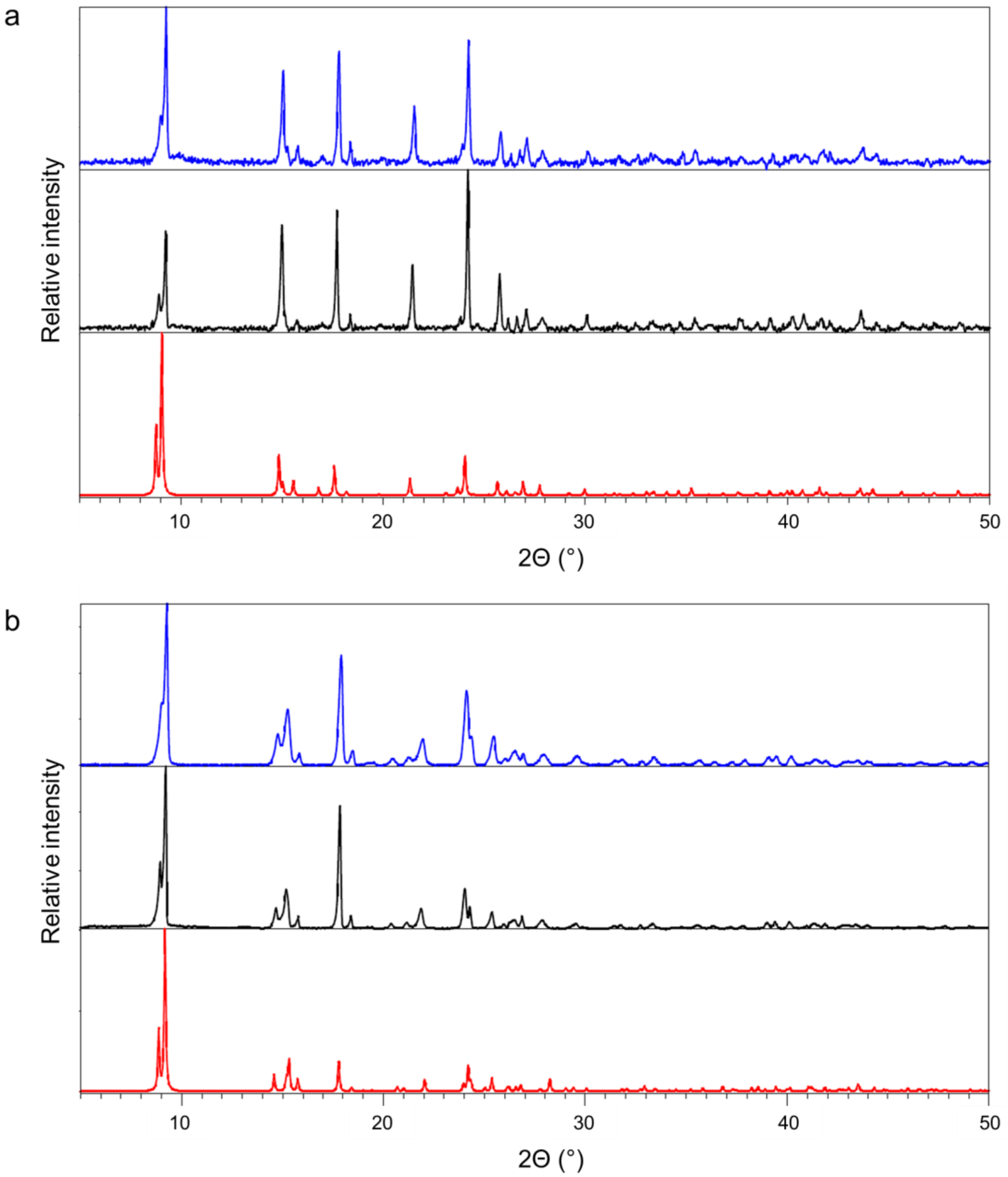

Figure S36 Powder X-ray diffractograms of (a) **L-chn-1-Co-NO3** and (b) **L-chn-1-Ni-NO3** before (black) and after (blue) 100 water vapor ad/desorption cycles (Figure S26). The PXRD patterns calculated from a crystal structure (Refcodes: (a) FOGGIT, (b) FOGGUF) retrieved from the CSD[10] using Mercury[12] is also shown (red).

4 Molecular Modelling

4.1 Density Functional Theory

All DFT simulations were performed using CP2K[36] with the PBE+D3 functional[37] and a mixed Gaussian-plane wave approach,[38] using Cut Off and Rel Cut Off parameters of 900 Ry and 60 Ry respectively unless otherwise mentioned.

4.1.1 Spin State Determination of Co Atoms

To determine the multiplicity that corresponds to the experimental **L-chn-1-Co-NO_3** system, geometry optimization calculations were performed establishing the lowest energy configuration. The four Co centers within the unit cell are each d^7 electron centers, and the competition between the electron pairing energy versus the d-orbital splitting energy is close depending on the chemical environment. As visualized in Figure S37, if pairing energy out-competes d-orbital splitting energy, the **L-chn-1-Co-NO_3** system will be high spin. In high spin with 4 Co centers per unit cell, each metal having three unpaired electrons, Hund's Rules dictate the multiplicity of the system will be 2(3/2×4)+1=13. If d-orbital splitting out-competes electron pairing energy and the system is low spin, there will be one unpaired electron per Co, such that 2 (1/2×4)+1=5.

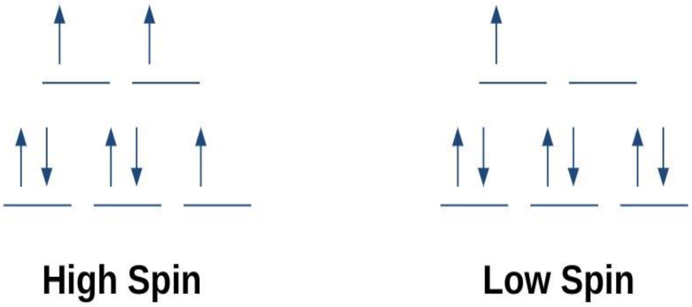

Figure S37 Illustration of the inferred spin states for the d^7 Co^{2+} atoms.

Generally, metals in an octahedral coordination environment are more stable with low spin electron configurations. However, the preference for high versus low spin for cobalt complexes varies.[39] Parallel geometry optimizations can isolate the more stable spin state by only varying the spin on the Co center. Whichever optimization yields the lowest energy is the more stable spin state,[40] where the difference between the spin states must be greater than thermal variation, which for molecular dynamics at 298 K will require approximately 2.48 kJ mol^{-1} difference or greater. Two geometry optimization calculations were run directly from the single unit cell (Refcode: FOGGIT). One geometry optimization was set to the high spin multiplicity (S=13) and the other was set to the low spin multiplicity (S=5).

Geometry optimization calculations on the single unit cell show that the high spin configuration is more stable than the low spin by 106.25 kJ mol^{-1} per unit cell, or 26.56 kJ mol^{-1} per Co metal center. This energetic difference sufficiently exceeds the thermal energy at 298 K (2.48 kJ mol^{-1}), so the high spin state is conclusively preferred for this system.

4.1.2 Cell Optimization

The PBE+D3 functional is known to model the structures of MOFs and similar framework structures well,[41] although it has a tendency for hydrogen bonds that are too short between sorbed water molecules.[42] Nonetheless, the PBE+D3 functional offers speed and has had success generating reasonable accuracy of water structure within metal organic frameworks.[43] The same crystal structure (CSD, Refcode: FOGGIT)[9] single unit cell underwent a cell optimization.

The results of the cell optimization on the **L-chn-1-Co-NO3** single unit cell are shown in Table S3, comparing the percentage difference between the high and low spin states against the experimental crystal structure data. The high spin state captures the experiment within 1.10% difference, while the low spin state differs by as much as 4.93%. This is consistent with the conclusions of the geometry optimization, and hence, molecular dynamics will proceed using the high spin multiplicity of S=13.

Table S3 Percent difference between experimental and calculated unit cell parameters of **L-chn-1-Co-NO3** using the PBE+D3 functional at high spin and low spin.

Unit Cell Parameters	HS	LS
a (Å)	+0.34%	-0.83%
b (Å)	-0.12%	-4.44%
c (Å)	-1.10%	+0.31%
α (˚)	-0.01%	0.00%
β (˚)	0.00%	+0.01%
γ (˚)	+0.01%	+0.01%
Volume (Å^3)	-0.88%	-4.93%

For the **L-chn-1-Ni-NO3** single unit cell, the starting positions from the FOGGIT crystal structure were used and the Co atoms substituted for Ni. The cell optimization used a multiplicity of 9 to be consistent with a d^8 Ni^{2+} electron configuration. The resulting cell parameters were compared with the experiment in Table S4.

Table S4 Percent difference between experimental and calculated unit cell parameters of **L-chn-1-Ni-NO3** using the PBE+D3 functional.

Unit Cell Parameters	% Difference
a (Å)	-0.77%
b (Å)	+1.23%
c (Å)	+0.72%
α (˚)	-0.07%
β (˚)	+2.73%
γ (˚)	-0.01 %
Volume (Å^3)	-0.63%

4.1.3 Molecular Dynamics

Figures and movies were generated using VMD.[44] All molecular dynamics was performed in the NVT ensemble using a Nose-Hoover thermostat set to 298 K, a time step of 1.0 fs, and a time constant of 100 fs. The atomic basis sets for C, H, O, and N were TZVP-MOLOPT-GTH while TZVP-MOLOPT-SR-GTH was used for Co and Ni.[37] Since the water loadings at full capacity (determined by DVS measurements) are non-stoichiometric and range between 6 and 7 H_2O/UC, molecular dynamics was carried out for loadings of 6 and 7 H_2O/UC for both **L-chn-1-Co-NO3** and **L-chn-1-Ni-NO3**. In the discussion that follows, the calculations are designated **M-U-SH2O** where **M** = metal, **U** = unit cell size (**S** = single; **E** = extended) and **S** = number of sorbates.

*4.1.3.1 **L-chn-1-Co-NO3**: Single Unit Cell 7 H_2O (**Co-S-7H2O**)*

The single unit cell simulation used the final coordinates from the cell optimization. To model the average of *ca* 6.4 waters experimentally determined from DVS analysis, 4 waters were manually inserted into one channel and 3 waters in the second channel. A multiplicity of 13 was imposed, as discussed in the Spin State Calculation section. The single unit cell with 7 waters ran for a total of 6971 fs steps, with the system achieving equilibrium after approximately 1500 steps as determined in Figure S38. The remaining 5471 fs were used for analysis and position averaging. See Movies S1 and S2.

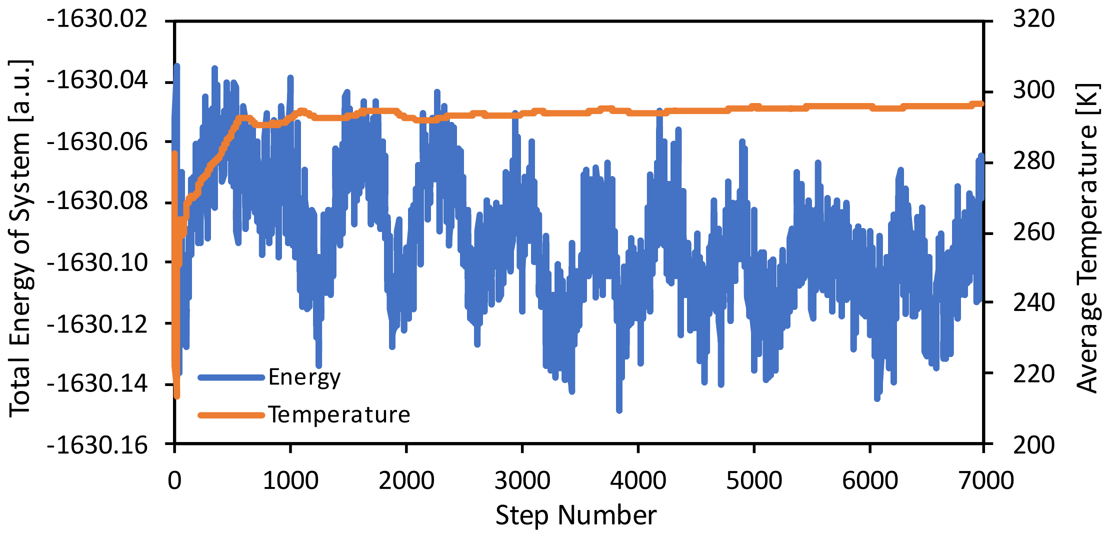

Figure S38 Energetic and temperature statistics of **Co-S-7H2O** as a function of step number.

*4.1.3.2 **L-chn-1-Co-NO3**: Single Unit Cell 6 H_2O (**Co-S-6H2O**)*

To investigate the impact of water concentration in the channels against the 6.4 H2O/uc determined by DVS, the **Co-S-6H2O** cell was generated by using the same atomic coordinates of the **Co-S-7H2O** unit cell, except that one water was deleted from the channel containing 4 waters, resulting in the **Co-S-6H2O** unit cell with 3 waters in each channel. This simulation ran for 4864 fs, equilibrating after 2000 fs as shown in Figure S39, leaving 2864 fs for analysis. See Movies S3 and S4.

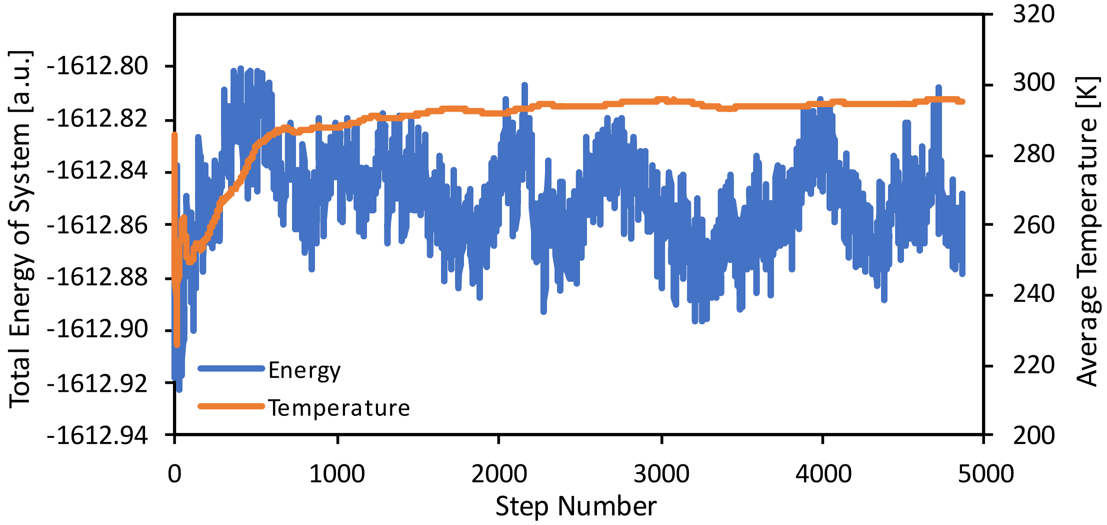

Figure S39 Energetic and temperature statistics of **Co-S-6H2O** as a function of step number.

*4.1.3.3 **L-chn-1-Co-NO3**: Extended Unit Cell 7 H_2O (**Co-E-7H2O**)*

Simulating 4 waters in one channel and 3 in the other is not sure to capture the experimentally observed average of *ca* 7 H_2O per unit cell. We were interested to know if the OPD maps generated by the **Co-S-7H2O** changed significantly if heterogeneity along the z axis were allowed. Hence, an extended unit cell, **Co-E-7H2O**, was created by imposing a multiplicity of 25, using the atomic coordinates of the **Co-S-7H2O** unit cell and repeating it once along the z axis. It ran for 5677 fs, achieving equilibrium around 1000 fs as shown in Figure S40, allowing 4677 fs for RDF and OPD map analysis. See Movies S5 and S6.

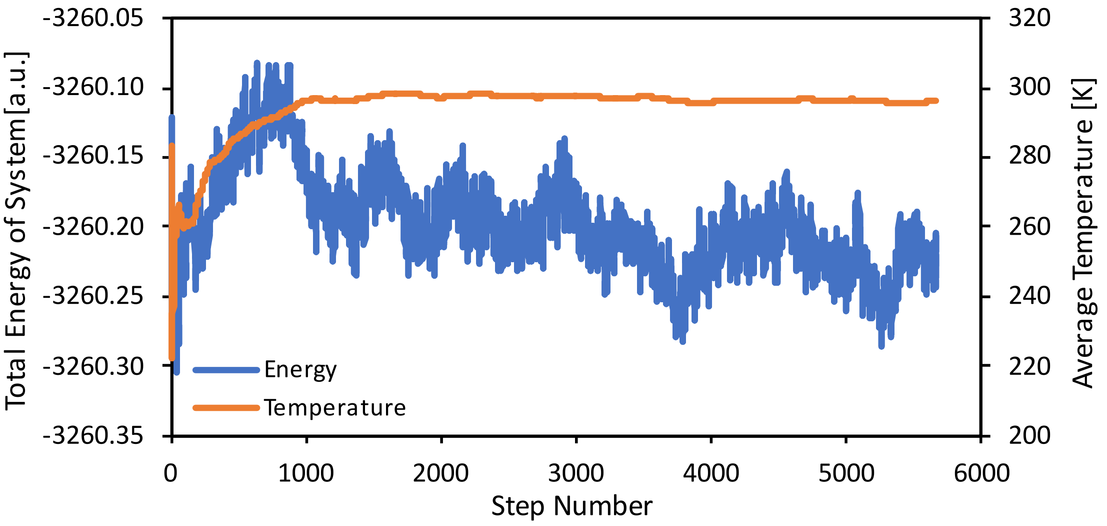

Figure S40 Energetic and temperature statistics of **Co-E-7H2O** as a function of step number.

*4.1.3.4 **L-chn-1-Ni-NO3**: Single Unit Cell 6 H_2O (**Ni-S-6H2O**)*

The final positions from the cell optimizations of **L-chn-1-Ni-NO3** were used for the **L-chn-1-Ni-NO3** framework positions in the following MD simulation. The same starting water positions from **Co-S-6H2O** were used as the initial positions for **Ni-S-6H2O**. Using a multiplicity of 9, the 6 H_2O unit cell ran for 5034 fs and equilibrated after approximated 1000 fs as shown in Figure S41, leaving 4034 fs for analysis. See Movies S7 and S8.

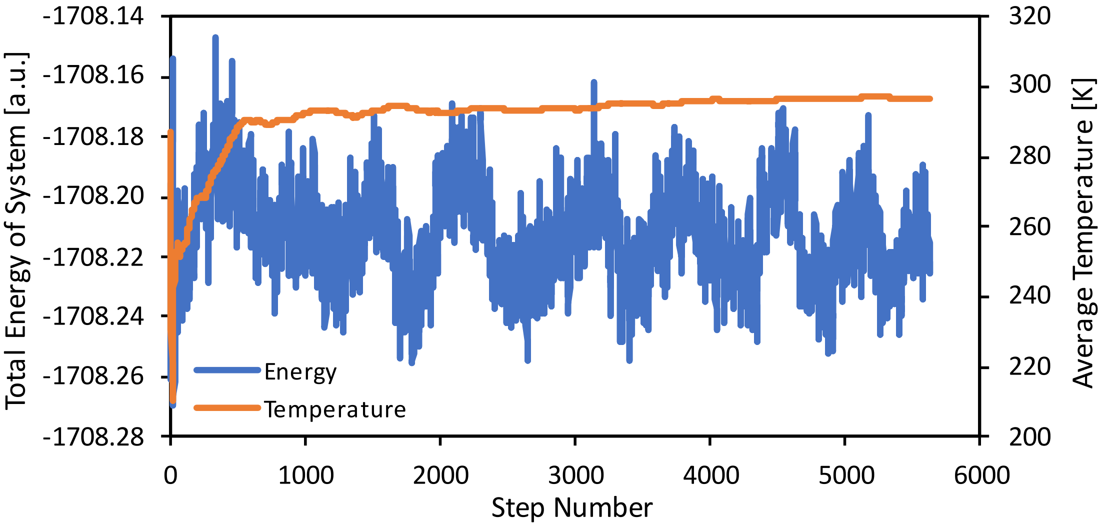

Figure S41 Energetic and temperature statistics of **Ni-S-6H2O** as a function of step number.

*4.1.3.5 **L-chn-1-Ni-NO3**: Single Unit Cell 7 H_2O (**Ni-S-7H2O**)*

The final positions from the cell optimizations of **L-chn-1-Ni-NO3** were used for the **L-chn-1-Ni-NO3** framework position. The same starting water positions from **Co-S-7H2O** were used as the initial positions for **Ni-S-7H2O**. Using a multiplicity of 9, the 7 H_2O unit cell ran for 4057 fs, also equilibrating after 1000 fs as shown in Figure S42, leaving 3057 fs for analysis. See Movies S9 and S10.

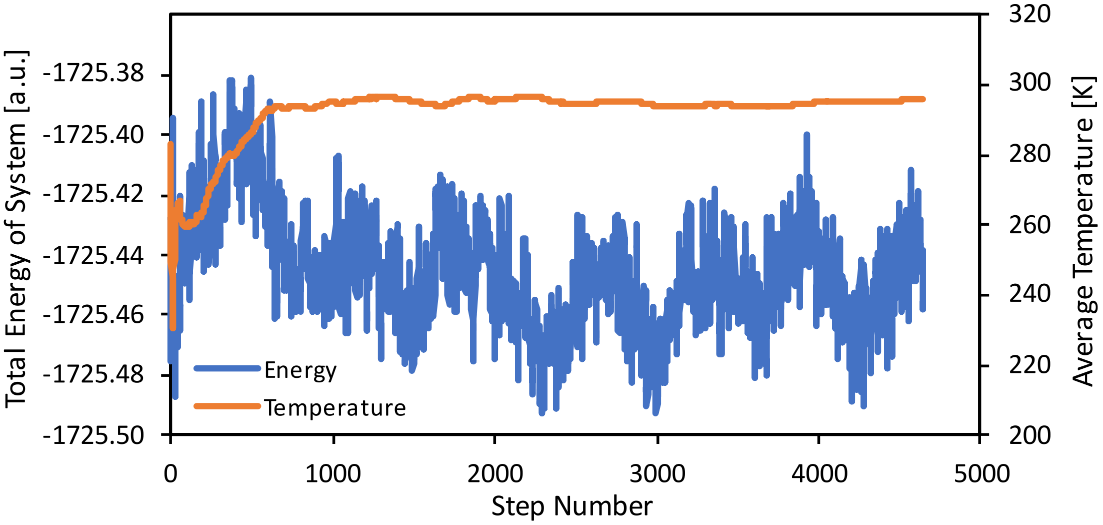

Figure S42 Energetic and temperature statistics of **Ni-S-7H2O** as a function of step number.

4.1.4 Radial Distribution Function Analysis

All RDF plots used coordinates from the equilibrated intervals of the MD simulations (**Co-S-7H2O**, 5471 fs; **Co-S-6H2O**, 2864 fs; **Co-E-7H2O**, 4677 fs; **Ni-S-6H2O**, 4034 fs; **Ni-S-7H2O**, 3057 fs) using MDTraj[45] and its *compute rdf* function with a bin width of 0.04 Å. In this work, in order to compare peak intensities across the unique unit cells with differing number of pairs, each RDF line was normalized to an arbitrarily chosen number of pairs, 56, such that:

$$Intensity = g(r) \times \left(\frac{p}{56}\right)$$

Where $g(r)$ is the intensity calculated by the *compute rdf* function, and p is the number of pairs for the specified pair. Hence, this normalization allows peak intensities to be relatively comparable to one another across all RDF figures.

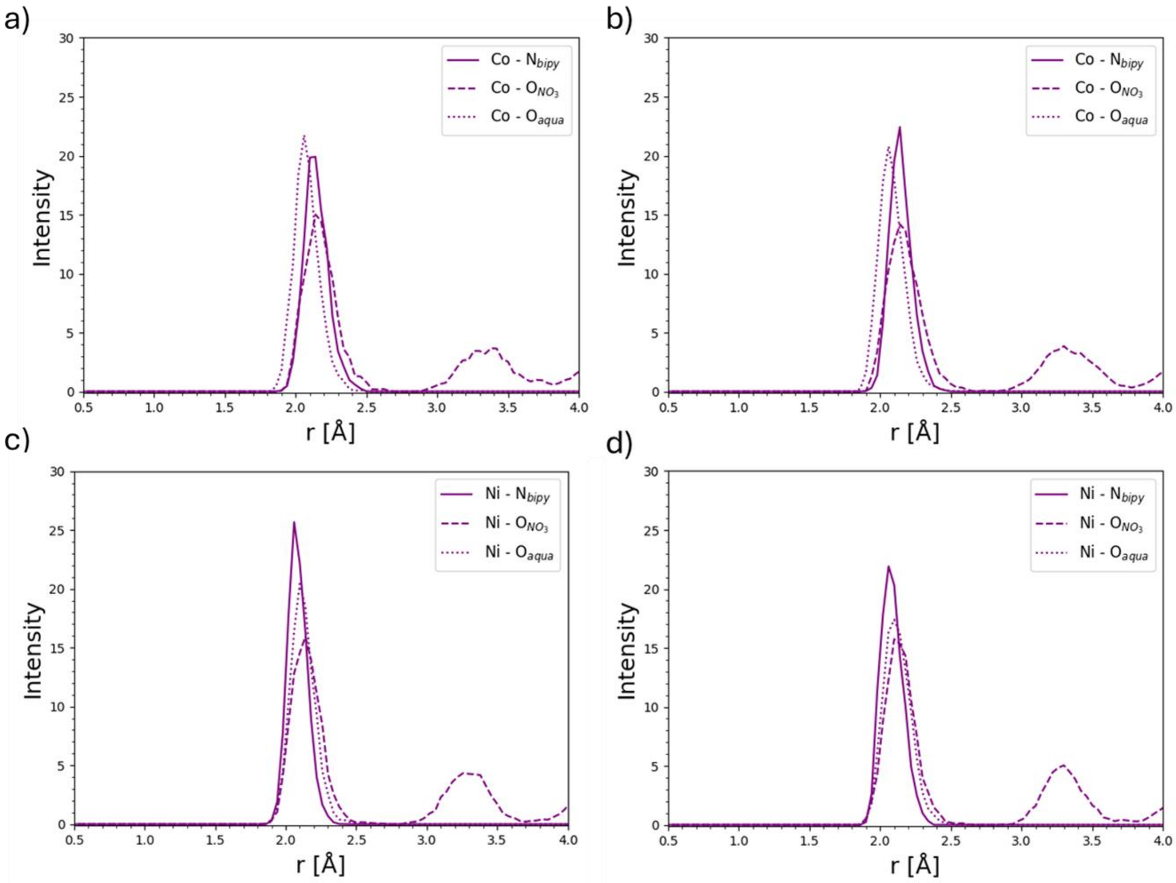

Figure S43 RDFs of the metal-ligand contacts within the single unit cells for a) **Co-S-6H2O**, b) **Co-S-7H2O**, c) **Ni-S-6H2O**, d) **Ni-S-7H2O**. All three oxygen atoms (coordinated and hydrogen-bonded) were included for NO_3 ligand analysis.

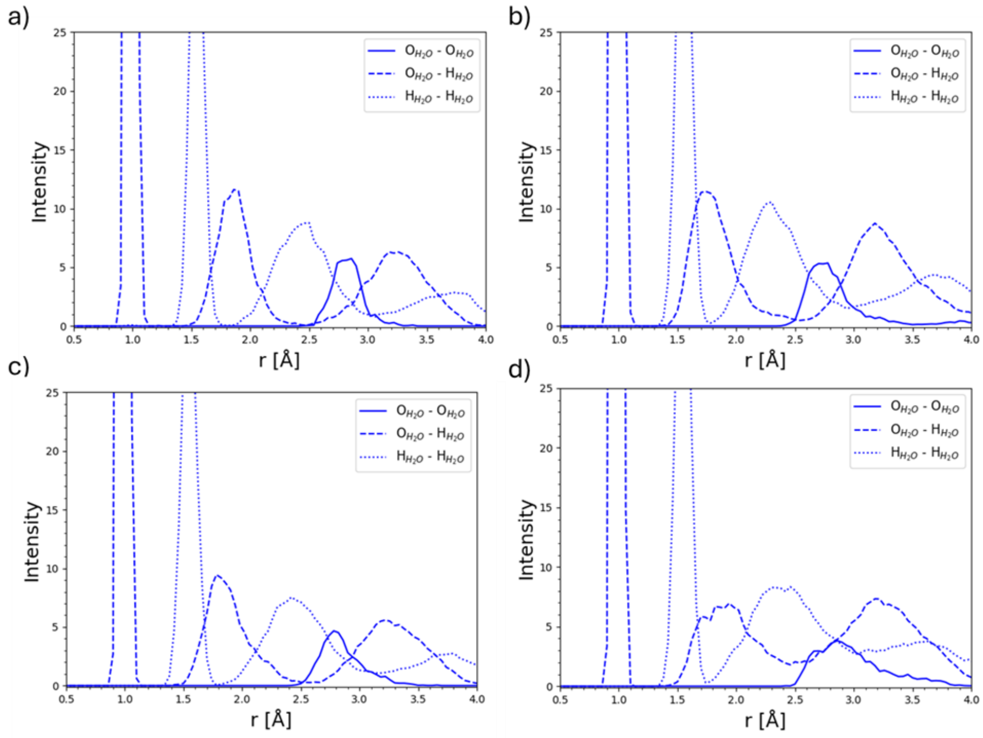

Figure S44 RDFs of the H_2O-H_2O contacts within the single unit cells for a) **Co-S-6H2O**, b) **Co-S-7H2O**, c) **Ni-S-6H2O**, d) **Ni-S-7H2O**.

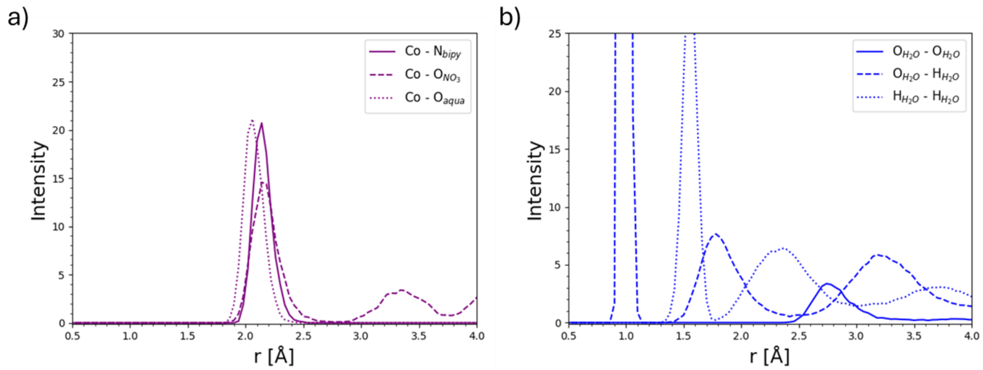

Figure S45. RDFs of the a) metal-ligand contacts and b) H_2O-H_2O contacts determined from **Co-E-7H2O**.

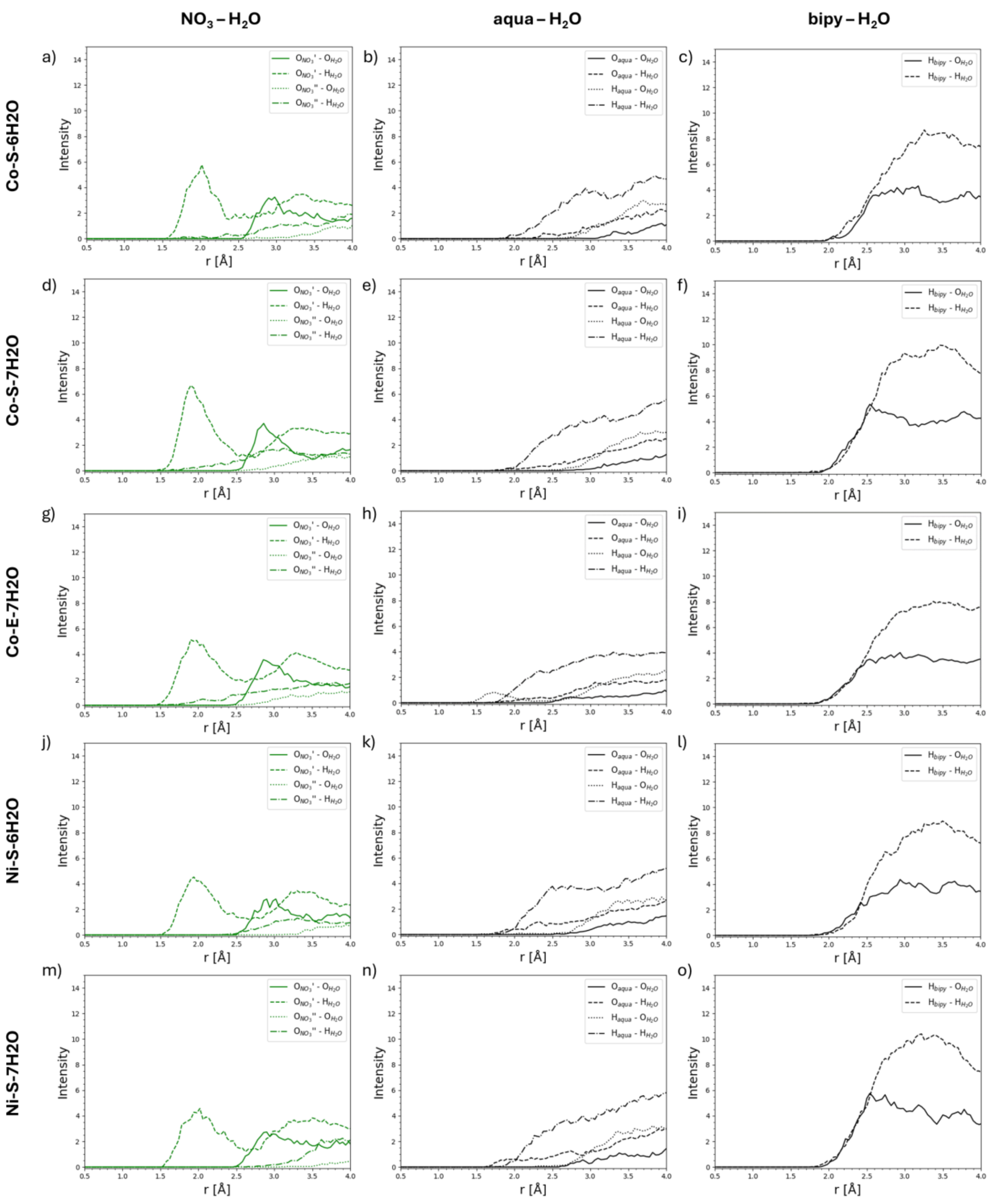

Figure S46 RDFs of key interatomic distances between water and framework atoms exposed to the channel for **Co-S-6H2O** (a – c), **Co-S-7H2O** (d – f), **Co-E-7H2O** (g – i), **Ni-S-6H2O** (j – l) and **Ni-S-6H2O** (m – o). The NO_3-H_2O (a, d, g, j, m), aqua-H_2O (b, e, h, k, n), and bipy-H_2O (c, f, i, l, o) contacts within the single unit cells.

Table S5 Modal interatomic distances (Å) determined by RDF analysis corresponding to the peaks observed in Figures S36-S38.

	**L-chn-1-Co-NO3**		**L-chn-1-Ni-NO3**	
Atom pairs	6 H_2O	7 H_2O	6 H_2O	7 H_2O
$M – N_{bipy}$	2.12	2.14	2.06	2.06
$M – O_{NO3}$	2.14, 3.30	2.14, 3.30	2.14, 3.30	2.10, 3.30
$M – O_{aqua}$	2.06	2.06	2.10	2.10
$O_{NO3}' – H_{H2O}$	2.90	2.86	2.90	2.90
$O_{NO3}' – O_{H2O}$	2.02	1.90	1.94	2.02
$O_{H2O} – H_{H2O}$	1.86, 3.18	1.74, 3.18	1.78, 3.21	1.84, 3.20
$O_{H2O} – O_{H2O}$	2.82	2.74	2.78	2.86

Table S6 Modal interatomic distances (Å) determined by RDF analysis corresponding to the peaks observed in Figure S41.

Atom pairs	Co-E-7H_2O
$M – N_{bipy}$	2.06
$M – O_{NO3}'$	2.15, 3.30
$M – O_{aqua}$	2.14
$O_{NO3}' – H_{H2O}$	2.89
$O_{NO3}' – O_{H2O}$	1.90, 3.28
$O_{H2O} – H_{H2O}$	0.98, 1.77, 3.17
$O_{H2O} – O_{H2O}$	2.76

4.1.5 Occupancy percentage distribution maps

Occupancy percentage distribution (OPD) maps were calculated using the VolMap analysis tool from VMD.[44] In summary of the VolMap documentation, OPD maps are calculated by generating a grid of 1.0 Å bin width for each frame of the trajectory. Each grid point is set to either 0 or 1 depending on whether an atom is present or not. These grids are then averaged together, giving a fractional occupancy of the final grid point. Multiple isosurfaces are then generated using different isovalues, or in other words, different fractional occupancies. These maps correspond to the fractional occupancy of sorbed water positions. The regions shaded in blue correspond to positions occupied by the oxygens of sorbed water molecules 10% of the time throughout the simulation, cyan to 70% of the time, and green to 90% of the time. There is no one configuration that corresponds to each of the regions above 90% occupancy, but the atomic positions of the framework and water shown have been averaged across equilibrium. The OPD maps are generated from the simulated unit cell, and then repeated in all directions to demonstrate surrounding symmetry, resulting in some overlap between the 10% occupancy regions.

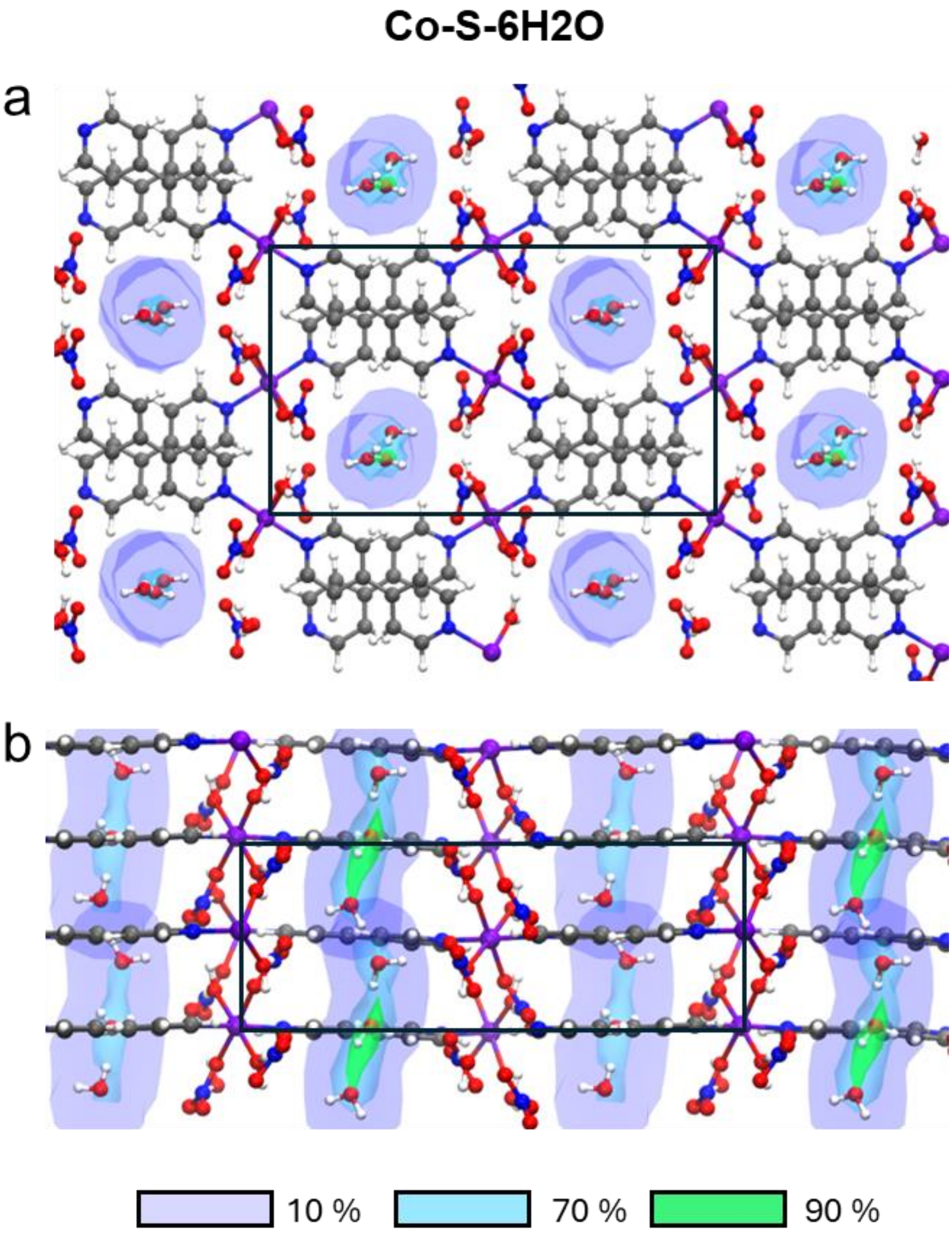

Figure S47. Structure from DFT simulations of the single unit cell with corresponding O_{H2O} occupancy maps of a) **Co-S-6H2O** along the *c* axis, b) **Co-S-6H2O** along the *a* axis. Fractional occupancies (0.1, blue; 0.7, cyan; 0.9, green) of channel water calculated from equilibrated frames. The simulated unit cell is boxed in black, with the structure extended to observe surrounding symmetry. Colors: oxygen, red; hydrogen, white; carbon, gray; nitrogen, purple; cobalt.

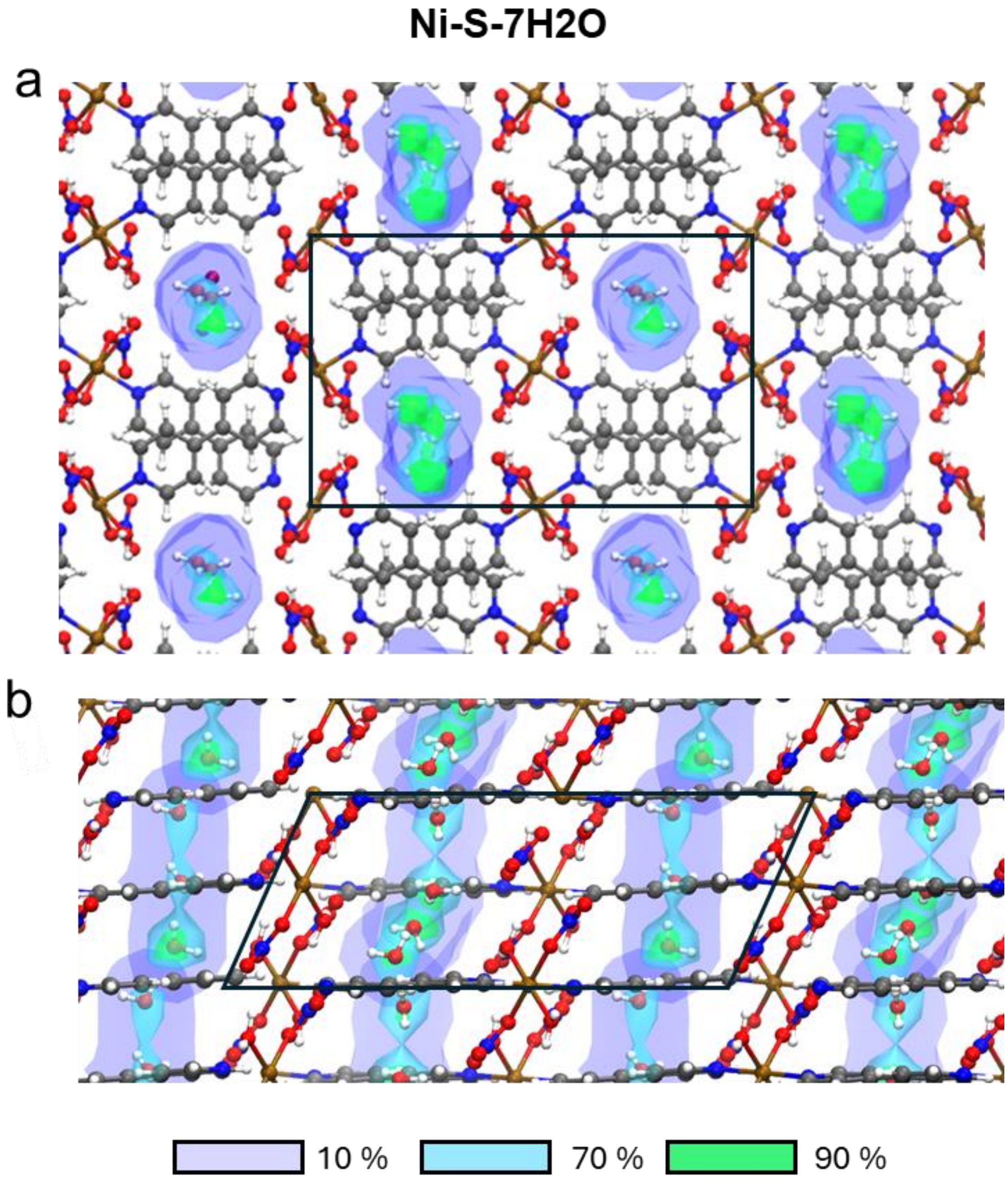

Figure S48. Structure from DFT simulations of the single unit cell with corresponding O_{H2O} occupancy maps of a) **Co-S-7H2O** along the *c* axis, b) **Co-S-7H2O** along the *b* axis. Fractional occupancies (0.1, blue; 0.7, cyan; 0.9, green) of channel water calculated from equilibrated frames. The simulated unit cell is boxed in black, with the structure extended to observe surrounding symmetry. Colors: oxygen, red; hydrogen, white; carbon, gray; nitrogen, brown; nickel.

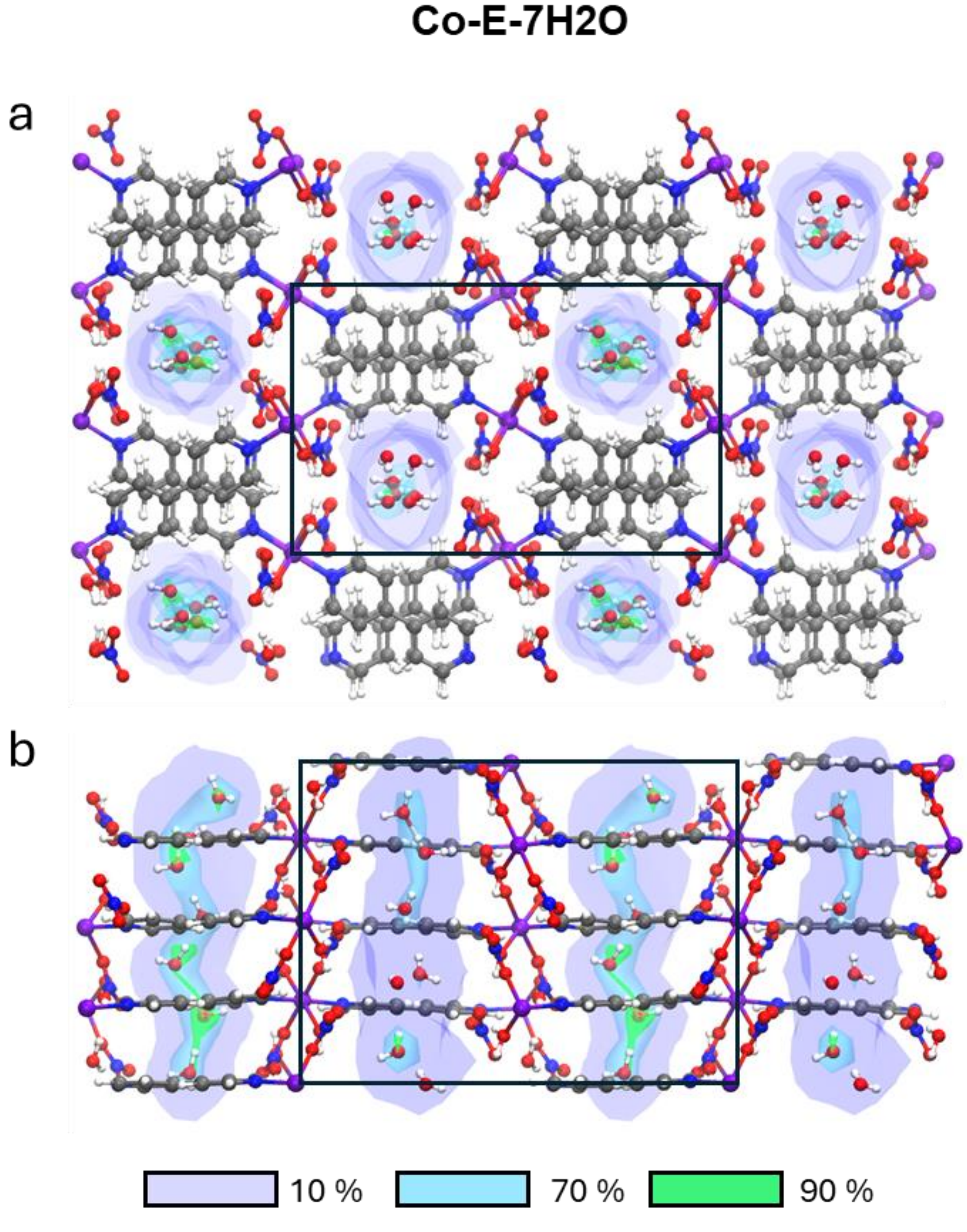

Figure S49. Structure from DFT simulations of the extended unit cell with corresponding O_{H2O} occupancy maps of a) **Co-E-7H2O** along the *c* axis, b) **Co-E-7H2O** along the *a* axis. Fractional occupancies (0.1, blue; 0.7, cyan; 0.9, green) of channel water calculated from equilibrated frames. The simulated unit cell is boxed in black, with the structure extended to observe surrounding symmetry. Colors: oxygen, red; hydrogen, white; carbon, gray; nitrogen, purple; cobalt.

4.2 Monte Carlo

Classical water adsorption experiments were carried out on a rigid 2x,1y,2z supercell of structure of **L-chn-Co-NO$_3$** unit cell to verify that similar results could be obtained with classical methods. The Massively Parallel Monte Carlo Code (MPMC) used in this work can be accessed here: https://github.com/mpmccode/mpmc. Intermolecular forces of the material were parameterized using the Universal Force Field (UFF),[32] RESP charges,[33] and Thole-Applequist type point polarizabilities[34] to model the van de Waals, electrostatic, and induced dipole effects respectively. TIP3P[35] was used as the water model. Canonical ensemble (NVT) simulations with simulated annealing were performed from the μVT equilibrated trajectories that contained 3, 25, and 31 H_2O per supercell or 1, 6, and 8 H_2O per unit cell rounding up. Equilibration was reached with the same parameters as in μVT but with a simulated annealing temperature of 20K. Cluster analysis was performed using MDTraj[10].

Simulated annealing of 3, 25, and 31 H_2O loading environments were analyzed for their clustering trends. Figures 8-10 shows the positional information of the O_{H2O} atom positions within **L-chn-Co-NO$_3$** as a probability distribution map for 25 and 31 H_2O/supercell respectively. Although water molecules could have been shown to be diffuse across the water channels, three different loading environments consistently displayed clustering behavior as consistent with DFT calculations. Simulated annealing showed adsorbed water near the NO_3 ligands. Additionally, the adsorbed waters prefer orientations where one of their waters are pointing in the direction of the NO_3 or aqua ligands, leaving their other hydrogen to point towards the center of the pore. As water loading increases, ordering of waters closer to the center of the channel becomes more complex, but the consistent orientation brought about by the NO_3 ligands never changes.

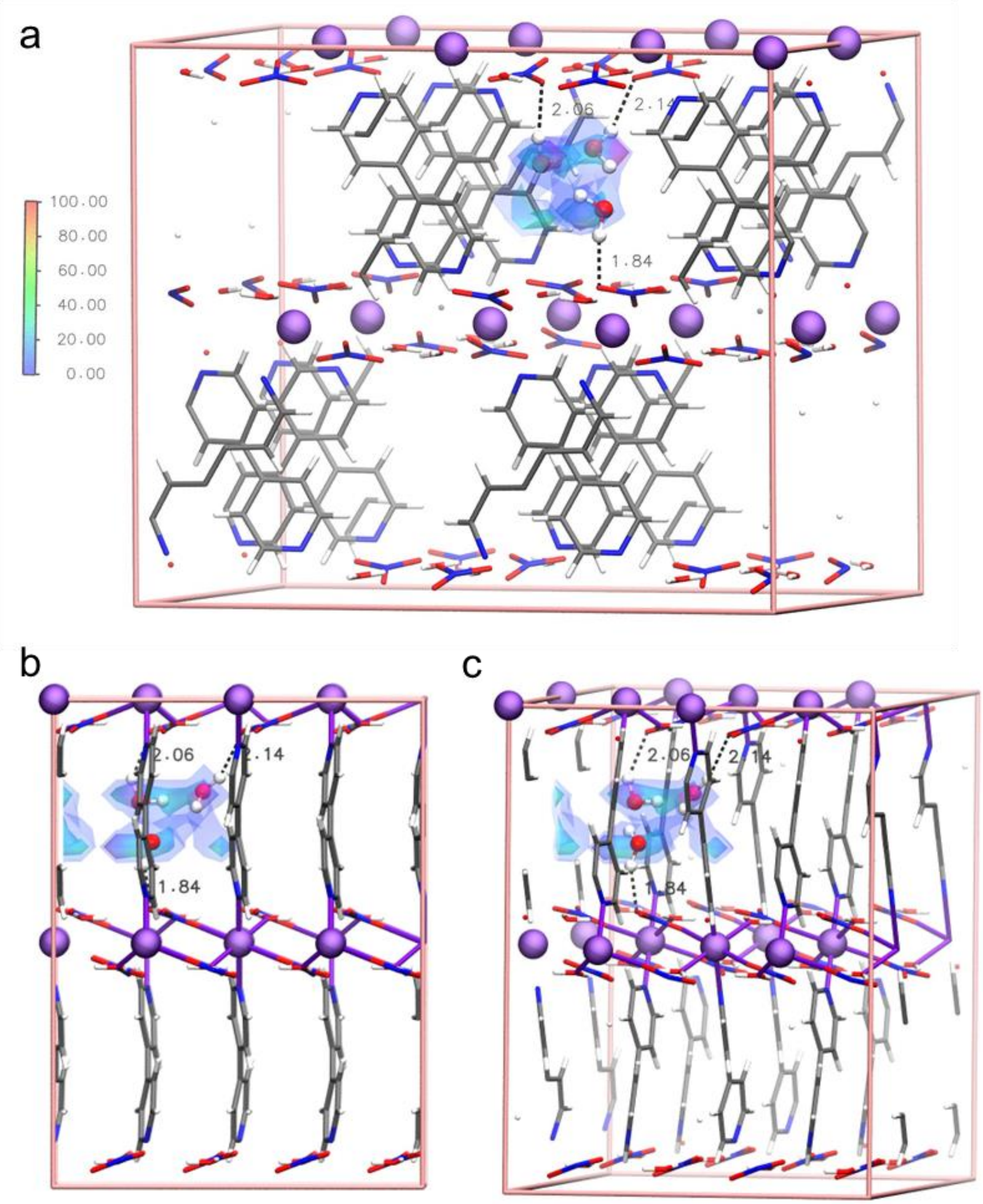

Figure S50. OPD maps of 3 H_2O/super cell shown from three different perspectives. Gradient from blue to red corresponds to increasing levels of occupancy across NVT simulations. Distances shown by black lines correspond to H_{H2O}-O_{NO3} distances.

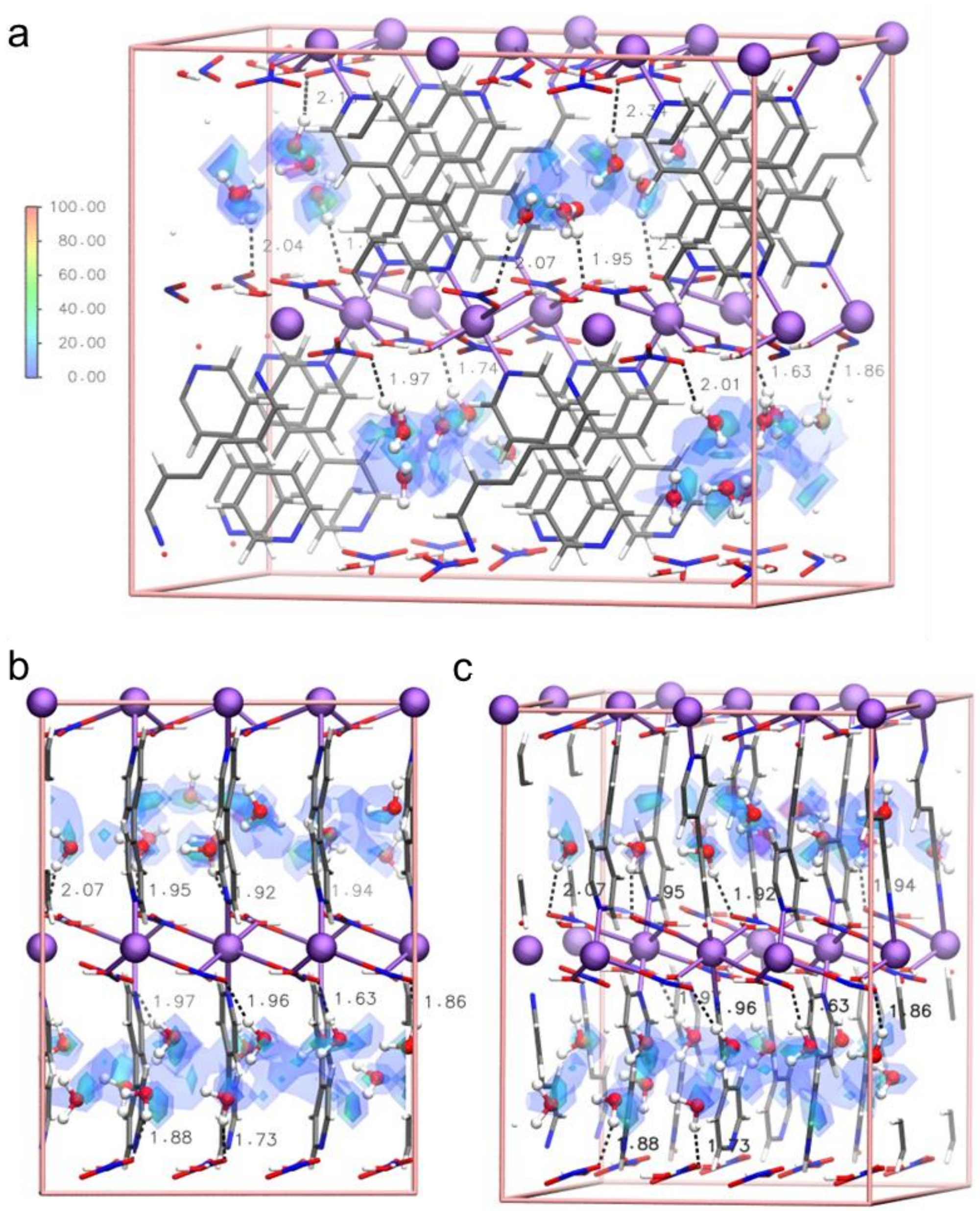

Figure S51. OPD maps of 25 H_2O/super cell shown from three different perspectives. Gradient from blue to red corresponds to increasing levels of occupancy across NVT simulations. Distances shown by black lines correspond to H_{H2O}-O_{NO3} distances.

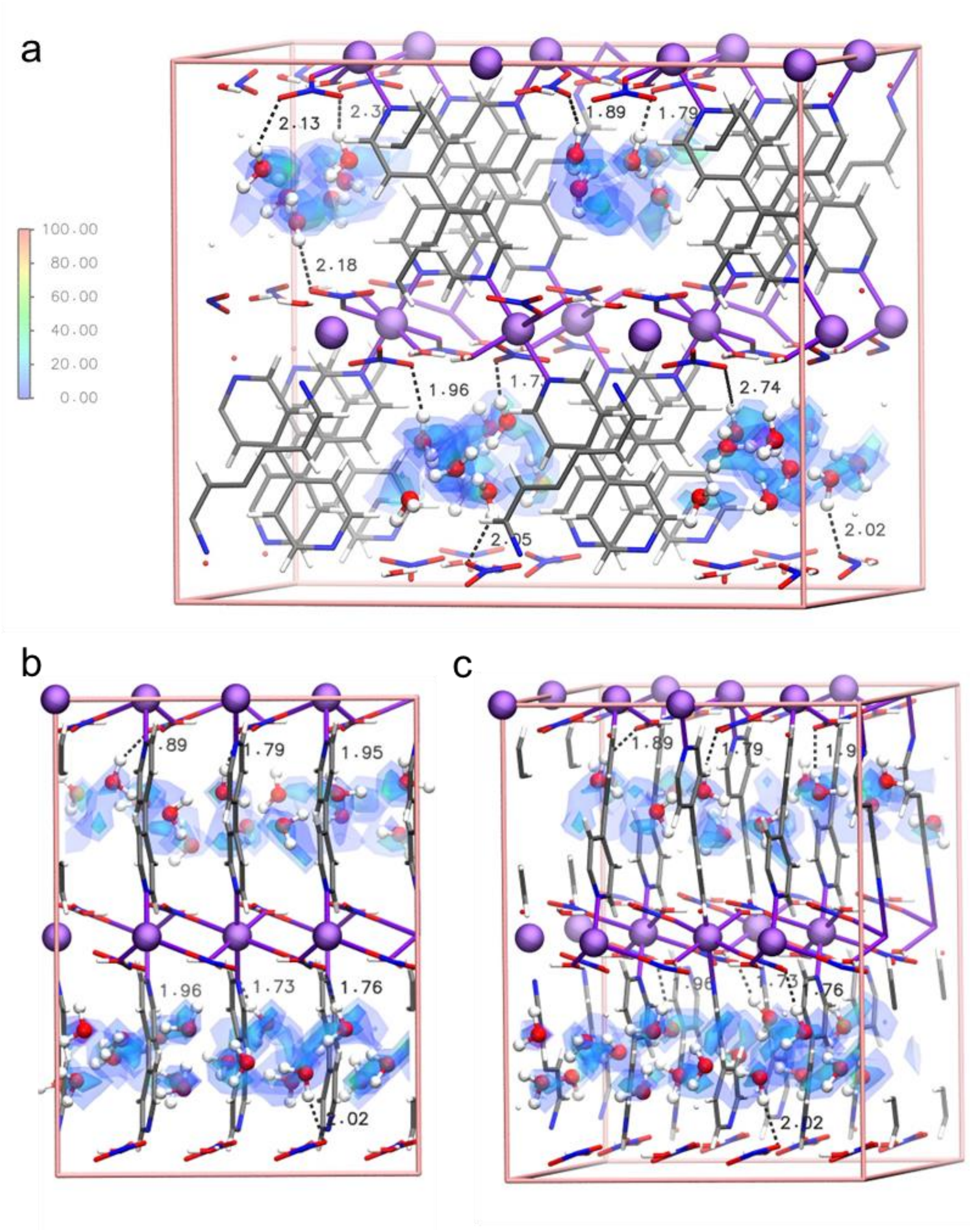

Figure S52. OPD maps of 31 H_2O/super cell shown from three different perspectives. Gradient from blue to red corresponds to increasing levels of occupancy across NVT simulations. Distances shown by black lines correspond to H_{H2O}-O_{NO3} distances.

5 Crystallographic Evaluation of the Favored Water Sorption Sites in **L-chn-1-Co-NO3** and **L-chn-1-Ni-NO3**

Considering that the DFT analysis showed that lattice water forms hydrogen bonds with the O_{NO3}' atoms (Figure S53) instead of the O_{aqua} or O_{NO3}'', and that the highest OPD intensities were in between atoms in the channel, the difference electron density maps of the crystal structures **L-chn-1-Co-NO3** and **L-chn-1-Ni-NO3** were examined further. Centroids c_1, c_2 and c_3 and c_1', c_2' and c_3' were generated between the nearest O_{NO3}' atoms (Figure S54) for **L-chn-1-Co-NO3** and **L-chn-1-Ni-NO3**, respectively. The O_{NO3}'–O_{NO3}' distances d_{c_1}, d_{c_2}, d_{c_3} and $d_{c_1'}$, $d_{c_2'}$, $d_{c_3'}$ were measured such that the line that connects the atoms passes through their respective centroids (e.g. distance d_{c_1} corresponds to the line that is bisected by centroid c_1). The values of $d_{c_1} = d_{c_3} =$ 8.0 Å and $d_{c_2} =$ 7.4 Å for the **L-chn-1-Co-NO3**, whereas $d_{c_1'} = d_{c_2'} = d_{c_3'} =$ 7.4 Å in **L-chn-1-Ni-NO3**.

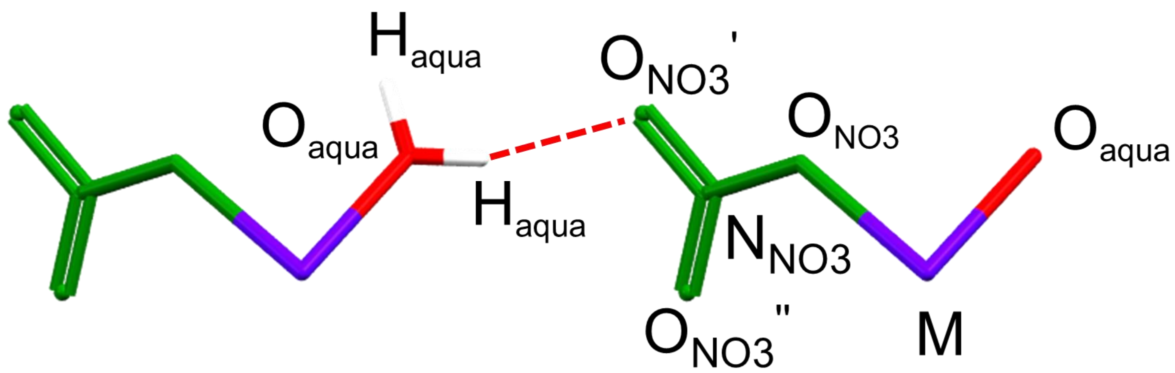

Figure S53 Capped sticks model of the functional groups exposed to the channel generated from SCXRD data showing the assignment of the three chemically distinct oxygen atoms in the NO_3 (green) group. The H_{aqua}-O_{NO3}' hydrogen bond interaction is shown with the O_{aqua} atoms colored red. The metal M (purple) is shown with other atoms omitted for clarity.

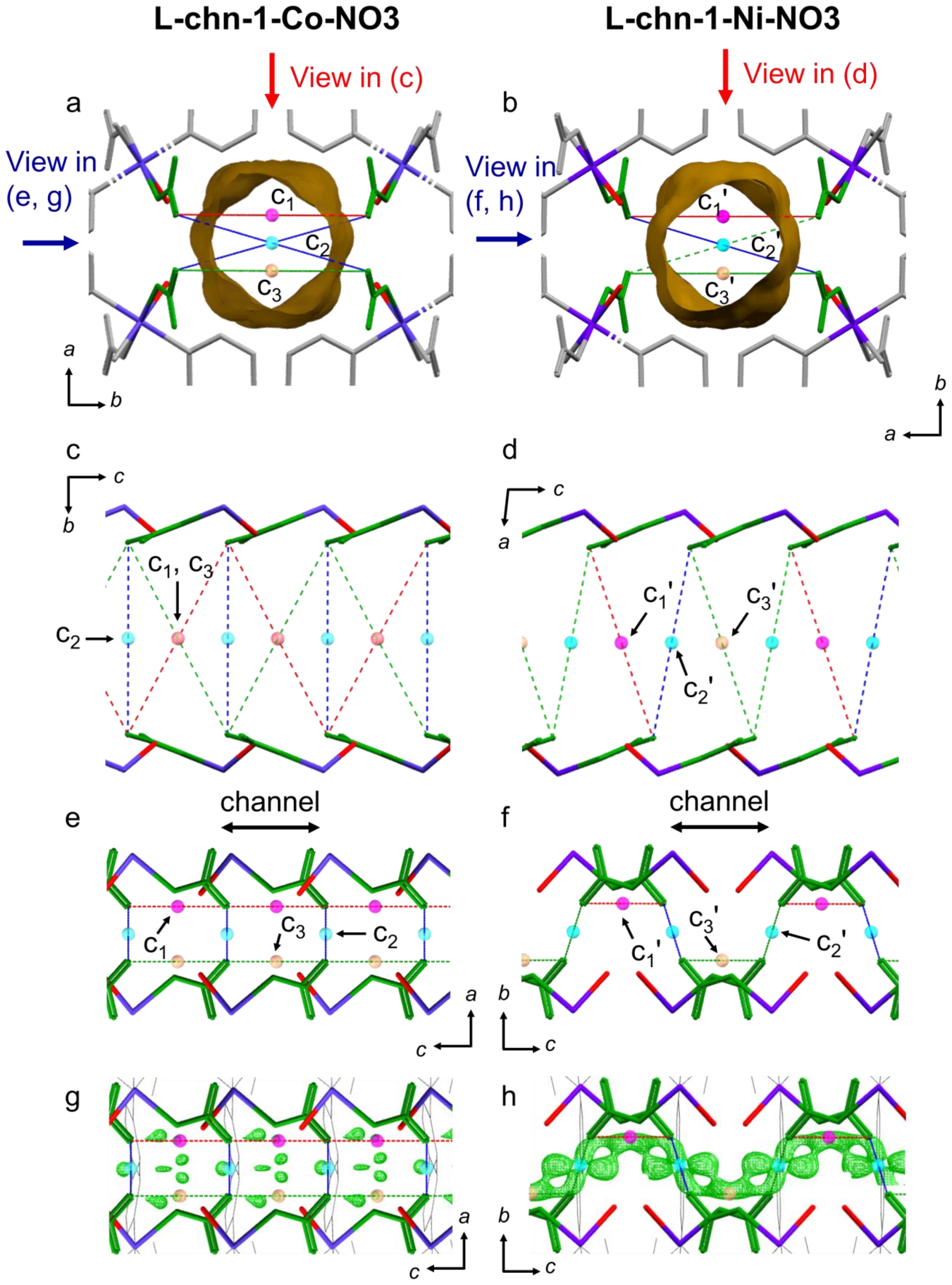
L-chn-1-Co-NO3
L-chn-1-Ni-NO3
View in (c)
View in (d)
View in (e, g)
View in (f, h)
a
b
c
d
e
f
g
h
channel
channel

Figure S54. Inspection of the centroids and distances between O_{NO3}' groups in the refined water-loaded crystal structures of **L-chn-1-Co-NO3** (left) and **L-chn-1-Ni-NO3 (right)** determined from SCXRD analysis at −173 °C. a) Projections of **L-chn-1-Co-NO3** and **L-chn-1-Ni-NO3** along [001] (a,b), **L-chn-1-Co-NO3** along (c) [100], **L-chn-1-Ni-NO3** along (d) [010], **L-chn-1-Co-NO3** along (e,g) [010] and **L-chn-1-Ni-NO3** along (f, h) [100]. Centroids c_1 (pink), c_2 (blue), c_3 (orange), c_1' (pink), c_2' (blue) and c_3' (orange) and O_{NO3}'–O_{NO3}' distances d_{c_1} (red), d_{c_2} (blue), d_{c_3} (green), $d_{c_1'}$ (red), $d_{c_2'}$ (blue) and $d_{c_3'}$ (green) are shown. Difference electron density maps shown in (g) and (h) (1.0 e^- $Å^{-3}$, green) were calculated using OLEX2.[11] Colors: atom colors shown in Figure S43 are used for functional groups exposed to the channels with the remaining atoms colored gray. Some atoms were omitted for clarity.

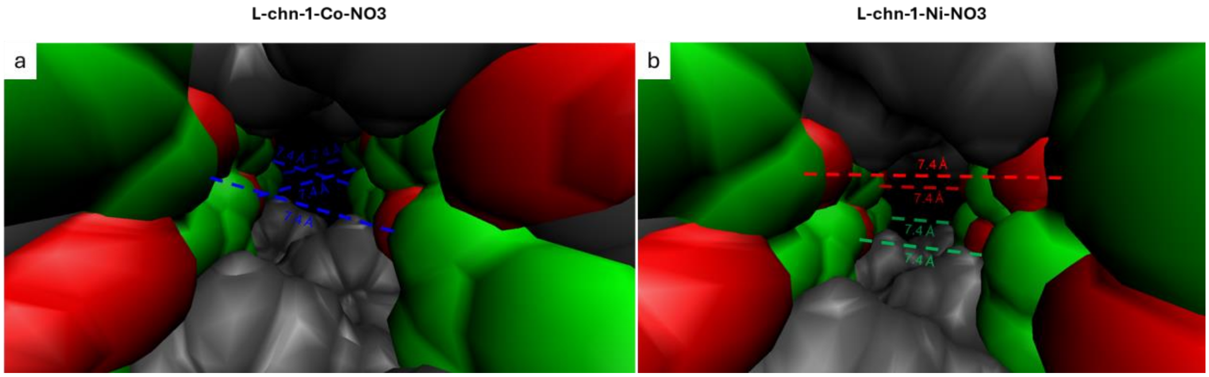

Figure S55 Projections generated in VMD along the a) **L-chn-1-Co-NO3** and b) **L-chn-1-Ni-NO3** channel axes showing distribution of O_{NO3}' atoms. Atom colors shown in Figure S45 are used for functional groups exposed to the channels with the remaining atoms colored gray.

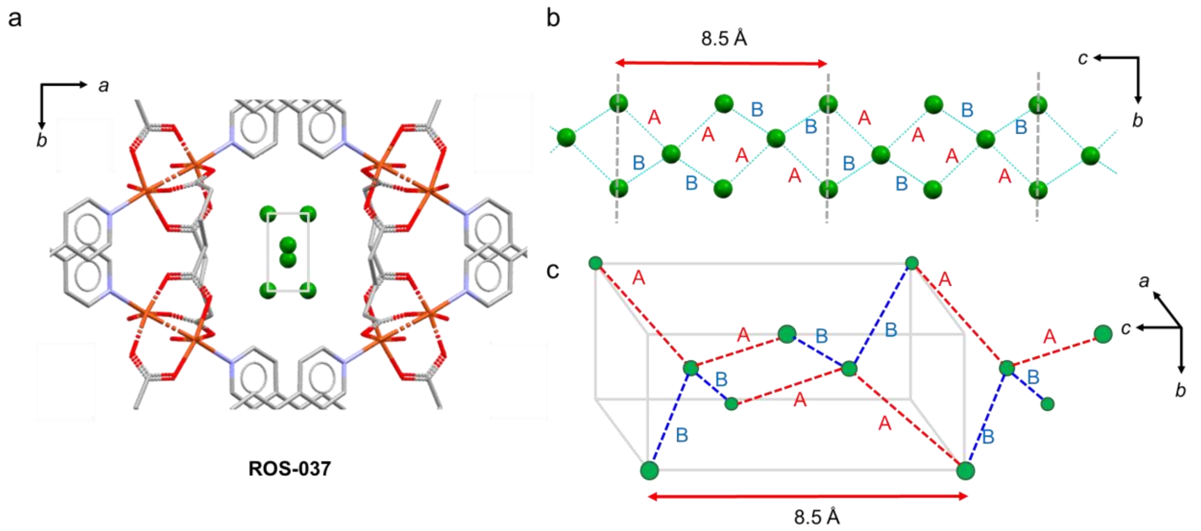

Figure S56 Clustering of water in **ROS-037** determined from a crystal structure deposited in the CSD (Refcode: MABJUV01). (a) Projection of **ROS-037** along [001] showing O_{H2O} atoms (green spheres). A gray rectangle is projected onto the figure such that its vertices are overlayed with the outermost O_{H2O} atoms. (b) Projection along [100] showing the close contact distances between O_{H2O} atoms with two crystallographically distinct distances A = 3.0 Å and B = 2.8 Å. The host molecules have been omitted for clarity and the unit cell boundary is shown with a gray dashed line. (c) Illustration of the water connectively in **ROS-037** where the O_{H2O} atoms (green circles) are positioned in accordance with the crystal structure. The gray rectangle corresponds to that shown in (a) with the longest edge corresponding to the length of the *c* axis.

6 Captions for Supplementary Movies

Movie S1: Projection of **L-chn-1-Co-NO3** along *a* axis taken from equilibrated steps in the **Co-S-7H2O** simulation. Occupancy percentage distribution maps of lattice water are shown as the blue shaded regions (0.1, blue; 0.7, cyan; 0.9, green).

Movie S2: Projection of **L-chn-1-Co-NO3** along *c* axis taken from equilibrated steps in the **Co-S-7H2O** simulation. Occupancy percentage distribution maps of lattice water are shown as the blue shaded regions (0.1, blue; 0.7, cyan; 0.9, green).

Movie S3: Projection of **L-chn-1-Co-NO3** along *a* axis taken from equilibrated steps in the **Co-S-6H2O** simulation. Occupancy percentage distribution maps of lattice water are shown as the blue shaded regions (0.1, blue; 0.7, cyan; 0.9, green).

Movie S4: Projection of **L-chn-1-Co-NO3** along *c* axis taken from equilibrated steps in the **Co-S-6H2O** simulation. Occupancy percentage distribution maps of lattice water are shown as the blue shaded regions (0.1, blue; 0.7, cyan; 0.9, green).

Movie S5: Projection of **L-chn-1-Co-NO3** along *a* axis taken from equilibrated steps in the **Co-E-7H2O** simulation. Occupancy percentage distribution maps of lattice water are shown as the blue shaded regions (0.1, blue; 0.7, cyan; 0.9, green).

Movie S6: Projection of **L-chn-1-Co-NO3** along *c* axis taken from equilibrated steps in the **Co-E-7H2O** simulation. Occupancy percentage distribution maps of lattice water are shown as the blue shaded regions (0.1, blue; 0.7, cyan; 0.9, green).

Movie S7: Projection of **L-chn-1-Ni-NO3** along *a* axis taken from equilibrated steps in the **Ni-S-6H2O** simulation. Occupancy percentage distribution maps of lattice water are shown as the blue shaded regions (0.1, blue; 0.7, cyan; 0.9, green).

Movie S8: Projection of **L-chn-1-Ni-NO3** along *c* axis taken from equilibrated steps in the **Ni-S-6H2O** simulation. Occupancy percentage distribution maps of lattice water are shown as the blue shaded regions (0.1, blue; 0.7, cyan; 0.9, green).

Movie S9: Projection of **L-chn-1-Ni-NO3** along *a* axis taken from equilibrated steps in the **Ni-S-7H2O** simulation. Occupancy percentage distribution maps of lattice water are shown as the blue shaded regions (0.1, blue; 0.7, cyan; 0.9, green).

Movie S10: Projection of **L-chn-1-Ni-NO3** along *c* axis taken from equilibrated steps in the **Ni-S-7H2O** simulation. Occupancy percentage distribution maps of lattice water are shown as the blue shaded regions (0.1, blue; 0.7, cyan; 0.9, green).

References

(1) Mauger-Sonnek, K.; Streicher, L. K.; Lamp, O. P.; Ellern, A.; Weeks, C. L. Structure control and sorption properties of porous coordination polymers prepared from $M(NO_3)_2$ and 4,4 '-bipyridine (M = Co^{2+}, Ni^{2+}). *Inorg. Chim. Acta* **2014**, *418*, 73-83.

(2) Bezrukov, A. A.; O'Hearn, D. J.; Gascon-Perez, V.; Darwish, S.; Kumar, A.; Sanda, S.; Kumar, N.; Francis, K.; Zaworotko, M. J. Metal-organic frameworks as regeneration optimized sorbents for atmospheric water harvesting. *Cell Rep.* **2023**, *4* (2), 101252.

(3) *SAINT*; Bruker AXS Inc.: Madison, Wisconsin, USA, 2012.

(4) *SADABS*; Bruker AXS Inc.: Madison, Wisconsin, USA, 2012.

(5) Sheldrick, G. M. SHELXT - Integrated space-group and crystal-structure determination. *Acta Crystallographica a-Foundation and Advances* **2015**, *71*, 3-8.

(6) (a) Barbour, L. J. X-Seed 4: updates to a program for small-molecule supramolecular crystallography. *J. Appl. Crystallogr.* **2020**, *53*, 1141-1146. (b) Atwood, J. L.; Barbour, L. J. Molecular graphics: From science to art. *Crystal Growth & Design* **2003**, *3* (1), 3-8.

(7) Sheldrick, G. M. Crystal structure refinement with SHELXL. *Acta Crystallographica Section C-Structural Chemistry* **2015**, *71*, 3-8.

(8) Groom, C. R.; Bruno, I. J.; Lightfoot, M. P.; Ward, S. C. The Cambridge Structural Database. *Acta Crystallogr. B: Struct. Sci. Cryst. Eng. Mater.* **2016**, *72*, 171-179.

(9) Bruno, I. J.; Cole, J. C.; Edgington, P. R.; Kessler, M.; Macrae, C. F.; McCabe, P.; Pearson, J.; Taylor, R. New software for searching the Cambridge Structural Database and visualizing crystal structures. *Acta Crystallogr. B: Struct. Sci. Cryst. Eng. Mater.* **2002**, *58*, 389-397.

(10) Macrae, C. F.; Edgington, P. R.; McCabe, P.; Pidcock, E.; Shields, G. P.; Taylor, R.; Towler, M.; van De Streek, J. Mercury: visualization and analysis of crystal structures. *J. Appl. Crystallogr.* **2006**, *39*, 453-457.

(11) Dolomanov, O. V.; Bourhis, L. J.; Gildea, R. J.; Howard, J. A. K.; Puschmann, H. OLEX2: a complete structure solution, refinement and analysis program. *J. Appl. Crystallogr.* **2009**, *42*, 339-341.

(12) Degen, T.; Sadki, M.; Bron, E.; König, U.; Nénert, G. The HighScore suite. *Powder Diffr.* **2014**, *29*, S13-S18.

(13) Brunauer, S.; Emmett, P. H.; Teller, E. Adsorption of Gases in Multimolecular Layers. *J. Am. Chem. Soc.* **1938**, *60* (2), 309-319.

(14) Llewellyn, P. L.; Rodriguez-Reinoso, F.; Rouquerol, J. Is the BET equation applicable to microporous adsorbents? In *Characterization of Porous Solids VII*, Llewellyn, P. L., Rodriguez-Reinoso, F., Rouquerol, J., Seaton, N. Eds.; Vol. 160; Elsevier, 2007; pp 49-56.

(15) Wang, X. L.; Qin, C.; Wang, E. B.; Xu, L. Supramolecular self-assembly of zigzag coordination polymer chains: A fascinating three-dimensional polycatenated network featuring an uneven "density of catenations" and a three-dimensional porous network. *Crystal Growth & Design* **2006**, *6* (9), 2061-2065.

(16) Sen, S.; Neogi, S.; Aijaz, A.; Xu, Q.; Bharadwaj, P. K. Structural variation in Zn(II) coordination polymers built with a semi-rigid tetracarboxylate and different pyridine linkers: synthesis and selective CO adsorption studies. *Dalton Trans.* **2014**, *43* (16), 6100-6107.

(17) Suckert, S.; Rams, M.; Rams, M. M.; Näther, C. Reversible and Topotactic Solvent Removal in a Magnetic Ni(NCS)
Coordination Polymer. *Inorg. Chem.* **2017**, *56* (14), 8007-8017.

(18) Lee, E. Y.; Suh, M. P. A robust porous material constructed of linear coordination polymer chains: Reversible single-crystal to single-crystal transformations upon dehydration and rehydration. *Angew. Chem. Int. Ed. Engl.* **2004**, *43* (21), 2798-2801.

(19) Wu, C. D.; Lin, W. B. Highly porous, homochiral metal-organic frameworks: Solvent-exchange-induced single-crystal to single-crystal transformations. *Angew. Chem. Int. Ed. Engl.* **2005**, *44* (13), 1958-1961.

(20) Wu, C. D.; Lin, W. B. Homochiral porous solids based on 1D coordination polymers built from 46-membered macrocycles. *Dalton Trans.* **2006**, (38), 4563-4569.
(21) Cheng, A. L.; Liu, N.; Yue, Y. F.; Jiang, Y. W.; Gao, E. Q.; Yan, C. H.; He, M. Y. Unprecedented 3D entanglement of 1D zigzag coordination polymers leading to a robust microporous framework. *Chem. Commun.* **2007**, (4), 407-409.
(22) Hu, S.; He, K. H.; Zeng, M. H.; Zou, H. H.; Jiang, Y. M. Crystalline-state guest-exchange and gas-adsorption phenomenon for a "Soft" supramolecular porous framework stacking by a rigid linear coordination polymer. *Inorg. Chem.* **2008**, *47* (12), 5218-5224.
(23) Kole, G. K.; Cairns, A. J.; Eddaoudi, M.; Vittal, J. J. Solvent-free porous framework resulted from 3D entanglement of 1D zigzag coordination polymer. *New J. Chem.* **2010**, *34* (11), 2392-2395.
(24) Xie, C. Z.; Su, Q. J.; Li, S. H.; Xu, J. Y.; Wang, L. Y. A novel helical chainlike nickel(II) coordination polymer containing 1D trigonal channels with water molecule guests. *Inorg. Chem. Commun.* **2010**, *13* (12), 1476-1479.
(25) Wang, D. X.; He, H. Y.; Chen, X. H.; Feng, S. Y.; Niu, Y. Z.; Sun, D. F. A 3D porous metal-organic framework constructed of 1D zigzag and helical chains exhibiting selective anion exchange. *Crystengcomm* **2010**, *12* (4), 1041-1043.
(26) Huang, S. L.; Zhang, L.; Lin, Y. J.; Jin, G. X. Discrepant gas adsorption in isostructural heterometallic coordination polymers: strong dependence of metal identity. *Crystengcomm* **2013**, *15* (1), 78-85.
(27) Jiang, X.; Tao, B.; Yu, X. L.; Wang, Y. H.; Xia, H. Syntheses, crystal structures and properties of three cyano-bridged one-dimensional coordination polymers based on macrocyclic metallic tectons. *RSC Adv.* **2015**, *5* (25), 19034-19040.
(28) Qu, L. Y.; Iguchi, H.; Takaishi, S.; Habib, F.; Leong, C. F.; D'Alessandro, D. M.; Yoshida, T.; Abe, H.; Nishibori, E.; Yamashita, M. Porous Molecular Conductor: Electrochemical Fabrication of Through-Space Conduction Pathways among Linear Coordination Polymers. *J. Am. Chem. Soc.* **2019**, *141* (17), 6802-6806.
(29) Leong, W. L.; Vittal, J. J. One-Dimensional Coordination Polymers: Complexity and Diversity in Structures, Properties, and Applications. *Chem. Rev.* **2011**, *111* (2), 688-764.
(30) (a) Spek, A. L. Single-crystal structure validation with the program. *J. Appl. Crystallogr.* **2003**, *36*, 7-13. (b) Spek, A. L. Structure validation in chemical crystallography. *Acta Crystallogr. D: Struct. Biol.* **2009**, *65*, 148-155.
(31) Spek, A. L. SQUEEZE: a tool for the calculation of the disordered solvent contribution to the calculated structure factors. *Acta Crystallographica Section C-Structural Chemistry* **2015**, *71*, 9-18.
(32) Gatta, G. D. Direct Determination of Adsorption Heats. *Thermochim. Acta* **1985**, *96* (2), 349-363.
(33) Kim, H.; Cho, H. J.; Narayanan, S.; Yang, S.; Furukawa, H.; Schiffres, S.; Li, X. S.; Zhang, Y. B.; Jiang, J. C.; Yaghi, O. M.; Wang, E. N. Characterization of Adsorption Enthalpy of Novel Water-Stable Zeolites and Metal-Organic Frameworks. *Sci Rep-Uk* **2016**, *6*.
(34) Campbell, C. T.; Sellers, J. R. V. Enthalpies and Entropies of Adsorption on Well-Defined Oxide Surfaces: Experimental Measurements. *Chem. Rev.* **2013**, *113* (6), 4106-4135.
(35) Zenodo. Metal-organic frameworks as regeneration optimized sorbents for atmospheric water harvesting. Zenodo, 2023.
(36) Kühne, T. D.; Iannuzzi, M.; Del Ben, M.; Rybkin, V. V.; Seewald, P.; Stein, F.; Laino, T.; Khaliullin, R. Z.; Schütt, O.; Schiffmann, F.; Golze, D.; Wilhelm, J.; Chulkov, S.; Bani-Hashemian, M. H.; Weber, V.; Borstnik, U.; Taillefumier, M.; Jakobovits, A. S.; Lazzaro, A.; Pabst, H.; Müller, T.; Schade, R.; Guidon, M.; Andermatt, S.; Holmberg, N.; Schenter, G. K.; Hehn, A.; Bussy, A.; Belleflamme, F.; Tabacchi, G.; Glöss, A.; Lass, M.; Bethune, I.; Mundy, C. J.; Plessl, C.; Watkins, M.; VandeVondele, J.; Krack, M.; Hutter, J. CP2K: An electronic structure and molecular dynamics software package - Quickstep: Efficient and accurate electronic structure calculations. *J. Chem. Phys.* **2020**, *152* (19).
(37) (a) Perdew, J. P.; Burke, K.; Ernzerhof, M. Generalized gradient approximation made simple (vol 77, pg 3865, 1996). *Phys. Rev. Lett.* **1997**, *78* (7), 1396-1396. (b) Perdew, J. P.; Burke, K.; Ernzerhof, M. Generalized gradient approximation made simple. *Phys. Rev. Lett.* **1996**, *77* (18), 3865-3868. (c) Grimme, S.; Ehrlich, S.; Goerigk, L. Effect of the Damping Function in Dispersion Corrected Density Functional

Theory. *J. Comput. Chem.* **2011**, *32* (7), 1456-1465. (d) Grimme, S.; Antony, J.; Ehrlich, S.; Krieg, H. A consistent and accurate ab initio parametrization of density functional dispersion correction (DFT-D) for the 94 elements H-Pu. *J. Chem. Phys.* **2010**, *132* (15).
(38) VandeVondele, J.; Krack, M.; Mohamed, F.; Parrinello, M.; Chassaing, T.; Hutter, J. QUICKSTEP: Fast and accurate density functional calculations using a mixed Gaussian and plane waves approach. *Comput. Phys. Commun.* **2005**, *167* (2), 103-128.
(39) Schmiedekamp, A. M.; Ryan, M. D.; Deeth, R. J. Six-coordinate Co^{2+} with H_2O and NH_3 ligands: Which spin state is more stable? *Inorg. Chem.* **2002**, *41* (22), 5733-5743.
(40) Henson, N. J.; Hay, P. J.; Redondo, A. Computational studies of cobalt-substituted aluminophosphates. *J. Phys. Chem. A* **2000**, *104* (11), 2423-2431.
(41) Nazarian, D.; Ganesh, P.; Sholl, D. S. Benchmarking density functional theory predictions of framework structures and properties in a chemically diverse test set of metal-organic frameworks. *J. Mater. Chem. A* **2015**, *3* (44), 22432-22440.
(42) Cockayne, E. Density functional theory meta GGA study of water adsorption in MIL-53(Cr). *Powder Diffr.* **2019**, *34* (3), 227-232.
(43) (a) Lenzen, D.; Zhao, J. J.; Ernst, S. J.; Wahiduzzaman, M.; Inge, A. K.; Fröhlich, D.; Xu, H. Y.; Bart, H. J.; Janiak, C.; Henninger, S.; Maurin, G.; Zou, X. D.; Stock, N. A metal-organic framework for efficient water-based ultra-low-temperature-driven cooling. *Nat. Commun.* **2019**, *10*, 3025. (b) You, W. Q.; Liu, Y.; Howe, J. D.; Sholl, D. S. Competitive Binding of Ethylene, Water, and Carbon Monoxide in Metal-Organic Framework Materials with Open Cu Sites. *Journal of Physical Chemistry C* **2018**, *122* (16), 8960-8966.
(44) Humphrey, W.; Dalke, A.; Schulten, K. VMD: Visual molecular dynamics. *Journal of Molecular Graphics & Modelling* **1996**, *14* (1), 33-38.
(45) McGibbon, R. T.; Beauchamp, K. A.; Harrigan, M. P.; Klein, C.; Swails, J. M.; Hernández, C. X.; Schwantes, C. R.; Wang, L. P.; Lane, T. J.; Pande, V. S. MDTraj: A Modern Open Library for the Analysis of Molecular Dynamics Trajectories. *Biophys. J.* **2015**, *109* (8), 1528-1532.
